# Cross‐sex shifts in two brain imaging phenotypes and their relation to polygenic scores for same‐sex sexual behavior: A study of 18,645 individuals from the UK Biobank

**DOI:** 10.1002/hbm.25370

**Published:** 2021-02-26

**Authors:** Christoph Abé, Alexander Lebedev, Ruyue Zhang, Lina Jonsson, Sarah E. Bergen, Martin Ingvar, Mikael Landén, Qazi Rahman

**Affiliations:** ^1^ Department of Clinical Neuroscience Karolinska Institutet Stockholm Sweden; ^2^ Department of Medical Epidemiology and Biostatistics Karolinska Institutet Stockholm Sweden; ^3^ Department of Psychiatry and Neurochemistry Institute of Neuroscience and Physiology, Sahlgrenska Academy, University of Gothenburg Gothenburg Sweden; ^4^ Department of Pharmacology Institute of Neuroscience and Physiology, Sahlgrenska Academy, University of Gothenburg Gothenburg Sweden; ^5^ Department of Psychology Institute of Psychiatry, Psychology, and Neuroscience, King's College London London UK

**Keywords:** classification, cortical volume, magnetic resonance imaging, polygenic scores, same‐sex sexual behavior, sexual orientation, UK Biobank

## Abstract

Genetic and hormonal factors have been suggested to influence human sexual orientation. Previous studied proposed brain differences related to sexual orientation and that these follow cross‐sex shifted patterns. However, the neurobiological correlates of sexual orientation and how genetic factors relate to brain structural variation remains largely unexplored. Using the largest neuroimaging‐genetics dataset available on same‐sex sexual behavior (SSB) (*n* = 18,645), we employed a data‐driven multivariate classification algorithm (PLS) on magnetic resonance imaging data from two imaging modalities to extract brain covariance patterns related to sex. Through analyses of latent variables, we tested for SSB‐related cross‐sex shifts in such patterns. Using genotype data, polygenic scores reflecting the genetic predisposition for SSB were computed and tested for associations with neuroimaging outcomes. Patterns important for classifying between males and females were less pronounced in non‐heterosexuals. Predominantly in non‐heterosexual females, multivariate brain patterns as represented by latent variables were shifted toward the opposite sex. Complementary univariate analyses revealed region specific SSB‐related differences in both males and females. Polygenic scores for SSB were associated with volume of lateral occipital and temporo‐occipital cortices. The present large‐scale study demonstrates multivariate neuroanatomical correlates of SSB, and tentatively suggests that genetic factors related to SSB may contribute to structural variation in certain brain structures. These findings support a neurobiological basis to the differences in human sexuality.

## Graphical abstract

The present study is the largest neuroimaging investigation and the first imaging‐genetics study on human sexual behavior to date. We demonstrate an association between brain structure and same‐sex sexual behavior (SSB), and suggest that genetic factors related to SSB contribute to brain structural variation. Our findings support a neurobiological basis to differences in human sexuality.

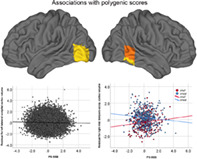

## 1 INTRODUCTION

The neurobiological basis of human sexual orientation is not well understood. Neuroimaging studies in this field are scarce and hindered by small sample sizes. Further research in larger samples may enhance our understanding of the origins and development of sexuality‐related variation in areas as diverse as behavior, cognition, and psychopathology (Bailey et al., 2016; Bailey & Zucker, 1995; Frisell, Lichtenstein, Rahman, & Langstrom, 2010; Li, Kung, & Hines, 2017; Rieger, Linsenmeier, Gygax, & Bailey, 2008; Sandfort, Graaf, Have, Ransome, & Schnabel, 2014; Xu, Norton, & Rahman, 2017). Whereas the evidence for psychosocial influences (such as upbringing) is weak, a large body of evidence supports the role of biological and non‐social environmental mechanisms in the development of sexual orientation (Bailey et al., 2016; Xu, Norton, & Rahman, 2019). Genetic factors and prenatal sex steroids appear to influence both sex differences in the brain and sexual orientation. It is hypothesized that these factors may shape brain development in such a manner as to organize sexuality and its behavioral correlates (Bailey, Dunne, & Martin, 2000; Bailey et al., 2016; Ganna & Verweij, 2019; Hines, 2011; Langstrom, Rahman, Carlstrom, & Lichtenstein, 2010; Rahman, 2005; Swaab & Garcia‐Falgueras, 2009).

Evidence for neural correlates to sexual orientation has also been accumulating. Early work using post mortem data suggested that the third interstitial nucleus of the anterior hypothalamus (INAH‐3) was smaller in homosexual men than in heterosexual men, and no different from heterosexual women (LeVay, 1991). Small‐scale neuroimaging studies have reported sexual orientation‐related differences in midline brain structures, including visual areas (Abé et al., 2018; Abé, Johansson, Allzen, & Savic, 2014; Manzouri & Savic, 2018b), volumetric patterns of hemispheric asymmetry (Savic & Lindström, 2008), gray matter volumes of the perirhinal cortex (Ponseti et al., 2007), and thickness of the orbitofrontal cortex (Abé et al., 2014). The pattern of these differences is such that in some brain areas homosexual males tend to be similar to heterosexual women (more female‐typical), and homosexual women tend to be similar to heterosexual men (more male‐typical). This is known as a cross‐sex shift and is also found in behavioral traits such as sex‐differentiated cognitive functions, personality, and gendered behavior (Allen & Robson, 2020; Bailey & Zucker, 1995; Xu et al., 2017). It is, therefore, not farfetched to assume that cognitive and behavioral traits related to sexual orientation may be reflected in brain differences. Recent investigations using other imaging modalities, such as those quantifying structural connectivity measured by fractional anisotropy (FA), did not report any sexual orientation‐related differences (Burke, Manzouri, & Savic, 2017; Manzouri & Savic, 2018a), but suggested that homosexuality may be associated with a less pronounced sexual differentiation in white matter tracts (Manzouri & Savic, 2018a).

However, the few previous imaging studies were limited by small sample sizes (often due to the difficulty in recruiting sufficient numbers of people with minority sexual orientation), were exploratory, and have not been replicated but produced conflicting results. The absence of female comparison groups in many studies also means we cannot interpret the neuroanatomical findings as cross‐sex shifts. Moreover, these studies also indicate that there may be complex multi‐modal brain endophenotypes related to sexual orientation. Previous studies analyzed univariate or average differences, which may mask more complex covariance patterns in the brain data, and could therefore not detect if sexual orientation manifests in multivariate neuroanatomical patterns. Data‐driven approaches to the analysis of brain data related to sexual orientation, employing methods of pattern recognition, may allow researchers to better quantify variation among many brain phenotypes simultaneously (Anderson, Harenski, & Harenski, 2019; Kurth, Gaser, & Luders, 2020).

Genetic factors explain about one third of the variation in sexual orientation (Bailey et al., 2000; Burri, Spector, & Rahman, 2015; Kendler, Thornton, Gilman, & Kessler, 2000; Langstrom et al., 2010). The twin concordance rate for sharing the same sexual orientation is estimated as 24% for both men and women (Bailey et al., 2016). The largest genome‐wide association study (GWAS) to date, including over 450,000 individuals, found that, in aggregate, all tested genetic variants accounted for 8–25% of variation in male and female same‐sex sexual behavior (SSB) and emphasizes that genetic influences on SSB are highly polygenic (Ganna & Verweij, 2019). The use of polygenic scores has become widely used in genetic research and brain imaging‐genetics when linking genetic and brain imaging data (Choi & Mak, 2020; Neilson et al., 2019). In the present context, an individual's polygenic score for SSB can be interpreted as the aggregated genetic predisposition to engage in SSB. Despite the strong evidence for genetic factors involved in sexual orientation, and that genetic mechanisms were hypothesized to influence the brain and thereby sexual orientation development (Rahman, 2005), no studies have empirically tested the relationship between polygenic factors and brain correlates of sexual orientation.

In the largest study on SSB to date, we applied multivariate pattern recognition tools on neuroimaging data from the UK Biobank to identify sexual orientation‐related cross‐sex shifts in brain imaging phenotypes extracted from two imaging modalities. In line with previous studies using large population‐based cohorts (Ganna & Verweij, 2019), we used self‐reported SSB as an indicator of sexual orientation (Bailey et al., 2016). We trained a multivariate classifier to separate males and females using structural volumetric and diffusion tensor imaging (DTI) data. After validation in an independent sample, we applied the resulting model on heterosexual and on non‐heterosexual males and females. Based on the cross‐sex shift theory, sex differences between non‐heterosexual men and women were expected to be less pronounced than between heterosexual men and women. Further, we used this method to predict the male‐/female‐likeness of participants' multivariate brain profiles and tested for a cross‐sex shift in the resulting covariance patterns. In secondary analyses, this was also performed for individual brain phenotypes. Finally, given the evidence for modest genetic influences on sexual orientation, we used genotype data to compute polygenic scores for SSB and correlated these with the investigated brain imaging phenotypes.

## 2 MATERIALS AND METHODS

### 2.1 Participants

UK Biobank (http://www.ukbiobank.ac.uk/) is a population‐based cohort study of over 500,000 individuals from the United Kingdom. Participants aged 40–69 were invited to one of 22 centers across the United Kingdom. Blood, DNA, urine, and saliva samples were collected, physical measurements taken, and each individual responded to health and lifestyle questionnaires (Allen et al., 2012; Miller et al., 2016). After the initial assessment (instance 0; 2006–2010) and first repeat visit (instance 1; 2012–2013), a subset of participants was re‐invited for magnetic resonance imaging (MRI) of the brain (instance 2; imaging visit 2014+). In total, 21,407 participants completed an MRI scan in Manchester, UK. Of those, 20,703 responded to the sexual behavior questionnaire (see below). Here, we excluded individuals with an ICD‐10 diagnosis of Gender Identity Disorder (F64, *n* = 2). Due to elevated psychiatric morbidity in non‐heterosexual individuals (Abé et al., 2018; Branstrom, 2017; Frisell et al., 2010; Plöderl & Tremblay, 2015; Sandfort et al., 2014; Sandfort, de Graaf, Bijl, & Schnabel, 2001), participants with psychiatric diagnoses were retained in the main analysis, but these were statistically controlled for in additional tests (see below).

UK Biobank has Research Tissue Bank approval from its governing Research Ethics Committee (REC), as recommended by the National Research Ethics Service (reference 11/NW/0382). All participants provided informed consent. The present study was approved by UK Biobank before data access was granted (proposal Nr. 41330). Further information on the recruitment and consent procedures can be found at: https://www.ukbiobank.ac.uk/key-documents/.

### 2.2 Brain image acquisition and processing

Details on image acquisition and processing can be found in the Supporting Information.

In brief, T1‐weighted structural and DTI images were acquired on a single scanner (Siemens Skyra 3 T) equipped with a standard Siemens 32‐channel head coil.

Our study made use of pre‐processed data and imaging‐derived phenotypes generated by an image processing pipeline developed and run on behalf of UK Biobank (Alfaro‐Almagro et al., 2018). In brief, T1 weighted 3D MPRAGE images (resolution: 1 × 1 × 1 mm^3^, matrix size: 208 × 256 × 256) were processed using fMRIB Software Library (FSL) tools (http://www.fmrib.ox.ac.uk/fsl). DTI data (2 × 2 × 2 mm^3^, 104 × 104 × 72, 2 × 50 directions) were processed with BEDPOSTx, followed by probabilistic tractography using PROBTRACKx. The main outcome measures were volumes of subcortical and cortical regions defined in subjects' native space segmented using the Harvard‐Oxford atlas, and DTI‐based FA values averaged over predefined major white matter tracts. These data were available for 18,757 out of 20,701 individuals.

### 2.3 Same‐sex sexual behavior

Self‐reported SSB was assessed through a computerized touchscreen questionnaire. Participants were asked: “Have you ever had sexual intercourse with someone of the same sex?” with sexual intercourse defined as vaginal, oral, or anal intercourse, making the measure unambiguous (UK Biobank Data‐Field 2159). Answering options were: “Yes,”, “No,” and “Prefer not to answer”. The questionnaire was administered at three visits (instances 0–2). Thus, data were available for at least one, but for some participants for two or three occasions. Participants who replied “Prefer not to answer” (“non‐responders”; 14 males, 12 females) were excluded and this analysis focused on those that congruently answered with “Yes” or “No.” Since any level of same‐sex sexuality captured through attraction, identity, or behavior may confer membership as non‐heterosexual (Norris, Marcus, & Green, 2015), males (M) and females (F) who congruently answered “Yes,” meaning at each available time point or at the latest available one, hence reporting non‐heterosexual (nHe) sexual behavior were termed as nHeM and nHeF. Those who congruently answered “No,” indicating exclusive heterosexual sexual behavior, were termed HeM and HeF. The final analysis included 18,645 participants who had SSB, DTI, and structural MRI data (8,432 HeM, 9,488 HeF, 393 nHEM, and 332 nHeF).

### 2.4 Psychiatric morbidity

UK Biobank provided hospital records obtained through linkage to external medical providers. Given the elevated rate of psychiatric disorders in non‐heterosexual populations (Abé et al., 2018; Branstrom, 2017; Frisell et al., 2010; Plöderl & Tremblay, 2015; Sandfort et al., 2014; Sandfort et al., 2001), we tested the influence of common mental disorders on our main results. Diagnoses according to the International Classification of Diseases (ICD‐10) were binarily coded as present or not. In addition to separate diagnoses, we created a combined binary variable coding for the presence of any psychiatric disorder. More details are provided in the Supporting Information and Table S1.

### 2.5 Demographic variables and participants' characteristics

Ethnicity was collapsed into “white” and “non‐white” categories given the very large number of participants reporting being from a “white” ethnic background. Fluid intelligence was measured using 13 logical reasoning questions administered via a computer‐touchscreen interface with a 2‐min time limit for each. The maximum score was 13. Victimization was measured via self‐report on a questionnaire asking participants whether they had been a victim of physical violence or sexual assault. Response options were: “Never,” “Yes, but not in the last 12 months”, “Yes, within the last 12 months,” and “Prefer not to answer”. These were coded with “0” for “Never” and “1” for any other response. Handedness was measured via a simple self‐report with options “Right‐handed”, “Left‐handed”, “Use both right and left hand equally”, and “Prefer not to answer”. In all cases, “Do not know” and “Prefer not to answer” were treated as missing. Body mass index (BMI) was constructed from height and weight. Additional variables included number of brothers and sisters, number of older siblings, and birth weight (family factors).

### 2.6 Multivariate pattern analysis (PLS)

In the main analysis, we used a “data‐driven” approach to investigate whether SSB relates to cross‐sex shifts in multivariate patterns of two‐modal brain imaging phenotypes, including both structural and DTI MRI data. We used the partial least squares (PLS) algorithm (Wold, Ruhe, Wold, & Dunn, 1984) as a multivariate tool to extract brain‐trait covariance patterns. Unlike traditional mass‐univariate approaches, this technique can identify internal relationships between a large number of variables useful for capturing complex covariance patterns of brain organization and their relation to a trait of interest, such as sex and SSB. PLS is an effective algorithm that has been shown to perform well on a wide variety of classification and regression problems applied on brain imaging data (Koutsouleris et al., 2012; Krishnan, Williams, McIntosh, & Abdi, 2011; Lebedev et al., 2013). Due to its projective nature, this method can reduce high‐dimensional data into a small set of latent variables (LVs; Wold et al., 1984), where each LV represents a statistically distinct multivariate pattern of brain‐trait associations. Further, LV scores are typically normally distributed, which allows for parametric follow‐up analyses, estimation of feature importance, and corresponding confidence intervals using a jack‐knifing technique (Martens & Martens, 2000). Another important feature of PLS is the interpretability of the outcomes. Here, the analysis was designed so that these scores reflect a participant's male‐ or female‐likeness of his/her independent multivariate brain profiles.

**FIGURE 1:**
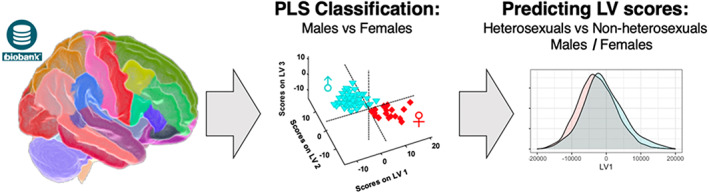
Workflow. Parcellated anatomical (cortical and subcortical volumes) and diffusion MRI data (tract FA) were first used to train a PLS classifier to differentiate males from females. Latent variable scores associated with biological sex were then extracted and further investigated for cross‐sex shift patterns. FA, fractional anisotropy; MRI, magnetic resonance imaging; PLS, partial least squares

The analysis was implemented in three steps: (1) training a classifier to separate males and females using cortical and subcortical volumetric and DTI neuroimaging data, (2) identifying relevant LVs that are important for the classification, and (3) assessing the relationships of the identified LVs with self‐reported SSB. According to the cross‐sex shift theory explained above, we expected (a) that a classifier would perform worse on non‐heterosexual individuals due to less pronounced sex‐related differences in morphometry, and (b) that individual LVs (capturing independent sex‐related multivariate brain patterns) should follow an SSB‐related cross‐sex shift pattern, that is, we expected sex‐by‐SSB interactions on LVs (main outcomes of interest). Figure 1 illustrates our workflow.

For PLS classification, we first randomly split the sample into a training (95% subjects, *n* = 17,713, 8,404 males, 9,309 females, 685 [4%] of those were non‐heterosexual) and a testing set (5% subjects, *n* = 932, 421 males, 511 females, 40 [4%] of those were non‐heterosexual). The latter was done to create a validation sample which had a sample size equivalent to the full set of non‐heterosexual individuals (*n* = 725; 393 males, 332 females) and to rule out data‐related generalization errors. The classifier was trained utilizing 10‐fold cross‐validation. We then performed post hoc tests assessing accuracy in heterosexuals only (*n* = 17,920) and finally in non‐heterosexuals only (*n* = 725). The number of LVs was determined based on cross‐validated classification accuracy (Figure S2). Assessment of sex‐by‐SSB interaction effects on LVs adhering to the main data analysis protocol was conducted (see section 2.7).

The PLS model was benchmarked against two alternative algorithms: logistic regression and an ensemble machine‐learning algorithm Random Forest (Figure S1). A reverse hypothesis was also tested by training PLS to predict SSB.

### 2.7 Cross‐sex shifts in LV (main analysis)

The effect of sex‐by‐SSB interaction on LV scores was tested using three 2 × 2 ANCOVAs crossing sex and SSB, including the main effects of age, sex, and SSB. We also performed post hoc group comparisons in LV scores, and tested for potential confounding effects by demographic and clinical variables. For the latter, we used the same model as above, but using additional covariates: BMI, ethnicity, fluid intelligence, smoking status (previous and current), self‐reported victimization, and each type of psychiatric diagnosis, including “any psychiatric diagnosis.” This was done for each variable at a time to increase sample size and power in confounder testing. We did not adjust for intracranial volume (ICV) because it relates to the phenotype of interest; adjusting the results for ICV would remove important variance related to the phenotype of interest (see Supporting Information for detailed explanation). To demonstrate this, we also performed an additional analysis adjusting for ICV for completeness. Effects of psychiatric diagnoses were also tested when including all psychiatric diagnosis variables in the model at the same time, and by repeating the main analysis when excluding all cases with any psychiatric diagnosis. Analysis was also repeated in right‐handed individuals only.

### 2.8 Cross‐sex shifts in DTI‐based FA‐values and volumetric measures (secondary analyses)

For completeness and to test for reproducibility of previous studies, a hypothesis driven region‐of‐interest (ROI) approach using mass‐univariate analyses of covariance testing for sex‐by‐SSB interactions on individual volumetric brain measures was conducted. This, an exploratory whole brain analysis, and an equivalent analysis on DTI‐based FA‐values are presented in the supplement. We also provide descriptives for each investigated measure and each group as well as effect sizes of group comparisons for the ROI approach (Figure 6) and for FA and volumetric whole brain analyses (Supporting Information and Data S2).

### 2.9 Participant characteristics

Differences in participant characteristics (Table 1), sociodemographic, health, cognitive measures and other variables were tested with univariate analyses of variance (four‐level group factor), *t*‐tests, or χ^2^ tests.

**TABLE 1:** Participants' characteristics

Group	HeM	HeF	nHeM	nHeF	p
*N* (18,645)	8,432	9,488	393	332	<.001
Age, M ± *SD*	63.41 ± 7.53	61.99 ± 7.25	60.01 ± 7.66	58.27 ± 7.02	<.001
BMI, M ± *SD*	27.06 ± 3.93	26.12 ± 4.64	27.43 ± 4.42	26.59 ± 4.37	<.001
Handedness % R/L/M	87.6/10.5/1.9	90.4/8.4/1.2	88.5/9.9/1.5	88.6/9.4/1.5	<.001
“White” ethnic background % (*n*)	97.4 (8193)	97.8 (9270)	96.2 (376)	95.8 (317)	.010
Fluid intelligence, M ± *SD*	7.23 ± 2.11	6.89 ± 1.97	7.06 ± 2.15	7.38 ± 2.05	<.001
Intracranial volume l, M ± *SD*	1.27 ± 0.10	1.14 ± 0.09	1.27 ± 0.10	1.16 ± 0.12	<.001
PS‐SSB	−0.013 ± 0.997	0.002 ± 0.998	0.135 ± 1.071	0.11 ± 1.002	.007
*Smoking*					
Previous smokers % (n)	37.1 (3097)	30.6 (2869)	39.7 (154)	43.3 (143)	<.001
Current smokers % (n)	4.1 (340)	3.2 (297)	9.3 (36)	7.0 (23)	<.001
*Family factors*					
Number of brothers, M ± *SD*	1.08 ± 1.20	1.13 ± 1.19	1.14 ± 1.28	1.04 ± 1.11	ns
Number of sisters, M ± *SD*	0.97 ± 1.09	1.03 ± 1.16	0.95 ± 1.07	1.03 ± 1.13	.003
Number of older siblings, M ± *SD*	1.04 ± 1.28	1.09 ± 1.27	1.18 ± 1.55	1.08 ± 1.31	.028
Birth weight kg, M ± *SD*	3.46 ± 0.63	3.28 ± 0.59	3.50 ± 0.61	3.35 ± 0.61	<.001
*Social stressors*					
Victim of physical violence % (*n*)	23.6 (1411)	12.9 (921)	37.4 (111)	22.6 (60)	<.001
Victim of sexual abuse % (*n*)	7.1 (423)	21.3 (1490)	20.1 (59)	41.9 (111)	<.001
*ICD‐10 diagnoses*					
Presence of any psychiatric disorder % (n)	3.6 (307)	4.1 (386)	8.1 (32)	6.6 (22)	<.001

*Note*: Means (M) and standard deviation (*SD)* of participants' characteristics or number of participants (n) are given for each group. BMI: body mass index. Statistical results (*p*‐value) for χ^2^‐tests or the four‐level group factor as predictor in univariate analyses of variance, respectively, are provided here to indicate the presence of overall group differences. A more detailed break down of individual psychiatric diagnoses is provided in Table S1.

### 2.10 Genome wide association study (GWAS) and generation of polygenic scores for SSB

Based on the findings by Ganna and Verweij (2019) indicating the polygenic nature of SSB, we used polygenic scoring to investigate genetic relationships between SSB and brain imaging outcomes. We first performed a GWAS on the SSB trait, similar to that conducted by Ganna and Verweij (2019), using genotype data from 393,973 individuals from the UK Biobank while excluding those subjects that were included in the present imaging study. Then, we generated polygenic scores for SSB (PS‐SSB) for each individual with imaging and genotype data (20,002 individuals). This individual score is a continuous measure that reflects the genetic predisposition to report SSB; the higher PS‐SSB, the higher the genetic predisposition. Analyses were restricted to individuals with European ancestry and genetic substructure was further controlled for using genetic principal components in the SSB GWAS. See Supporting Information for methodological details.

### 2.11 Associations between polygenic scores and brain imaging outcomes

We investigated the relationships between LVs and PS‐SSB using multiple linear regression analyses in SPSS 26. LVs were dependent variables. Age, sex, and PS‐SSB were independent variables, where PS‐SSB was the predictor variable of interest. We intentionally did not adjust for SSB, as this would disguise effects of interest by removing parts of the variance in brain imaging measures that we expect to be explained by PS‐SSB. For example, a detected association between brain structure and PS‐SSB driven by SSB‐related group differences in cortical volume can still reflect an influence of genetic factors on brain variation. However, in secondary tests, we tested the independent effects of PS‐SSB and SSB using both as regressors at the same time (see Supporting Information).

We tested two independent questions by performing the above‐mentioned linear regressions in the combined cohort. First, according to the cross‐sex shift theory, brain phenotypes of nHeM are shifted toward females, and those of nHeF and toward males. Therefore, the associations between PS‐SSB and brain outcomes should be of different directionality in males and females. For example, we expected that higher PS‐SSB relates to smaller brain volumes in males and to larger volumes in females. Hence, we investigated whether PS‐SSB has different associations with brain imaging outcomes in males and females by adding a sex‐by‐PS‐SSB interaction term to the model described above. Prior to this, to rule out that correlations were driven by the larger group of heterosexual participants and to ensure that the sex‐by‐PS‐SSB interaction is similar in hetero‐ and non‐heterosexual groups, we added a three‐way interaction term (SSB‐by‐sex‐by‐PS‐SSB; and corresponding two‐way terms) to the model and tested for the effect of SSB‐by‐sex‐by‐PS‐SSB. Second, since the present and some previous studies indicate a main effect of sexual orientation/SSB (sexual orientation‐related brain difference irrespective of sex), we tested for the main effect of PS‐SSB on brain imaging outcomes (without interaction terms in the model).

In addition to investigating PS‐SSB relationships to multivariate patterns (LVs), we explored the relationships between PS‐SSB and each individual brain imaging phenotype (individual cortical and subcortical volumes and FA‐values) in the same way as described above. We adjusted for multiple testing within each imaging modality using Bonferroni's Dubey Armitage‐Parmar/Sidak's adjustment of α‐level considering the number of tests and the inter‐correlation between the dependent variables (Sankoh, Huque, & Dubey, 1997).

To interpret significant interactions and main effects (after multiple comparison correction), we performed post hoc tests by quantifying the correlations between PS‐SSB and brain phenotypes within each group (HeM, HeF, nHeM, and nHeF). We further tested for potential confounding effects of demographic and psychiatric diagnosis variables (listed in Table 1) by adding these variables (one at a time) as covariates to the model.

In a complementary genetic analysis, we calculated genetic correlations linking previously published GWAS on SSB (Ganna & Verweij, 2019) and brain structure in the UK Biobank (Elliott et al., 2018) using genetic linkage disequilibrium score regression (LDSC; see Supporting Information).

## 3 RESULTS

### 3.1 Participants

In PLS analyses, we included individuals who provided both structural MRI and DTI data (*n* = 18,645). Sample characteristics are shown in Table 1. Four percent (*n* = 725) of the sample was classified as “non‐heterosexual,” which is consistent with population estimates (Gates, 2014). There were group differences in BMI, birth weight, and ICV, which were mainly driven by differences between males and females. Most participants reported a white ethnic background (ranging from 96 to 98% across groups). There were group differences in experiences of victimization, prevalence of psychiatric disorders, and fluid intelligence scores (Table 1). These were adjusted for in sensitivity analyses.

### 3.2 PLS classification

The PLS model outperformed the alternative logistic regression and ensemble machine‐learning algorithm Random Forest (Figure S1). This, together with the reasons outlined above, motivated us to continue subsequent analyses using PLS as intended.

**FIGURE 2:**
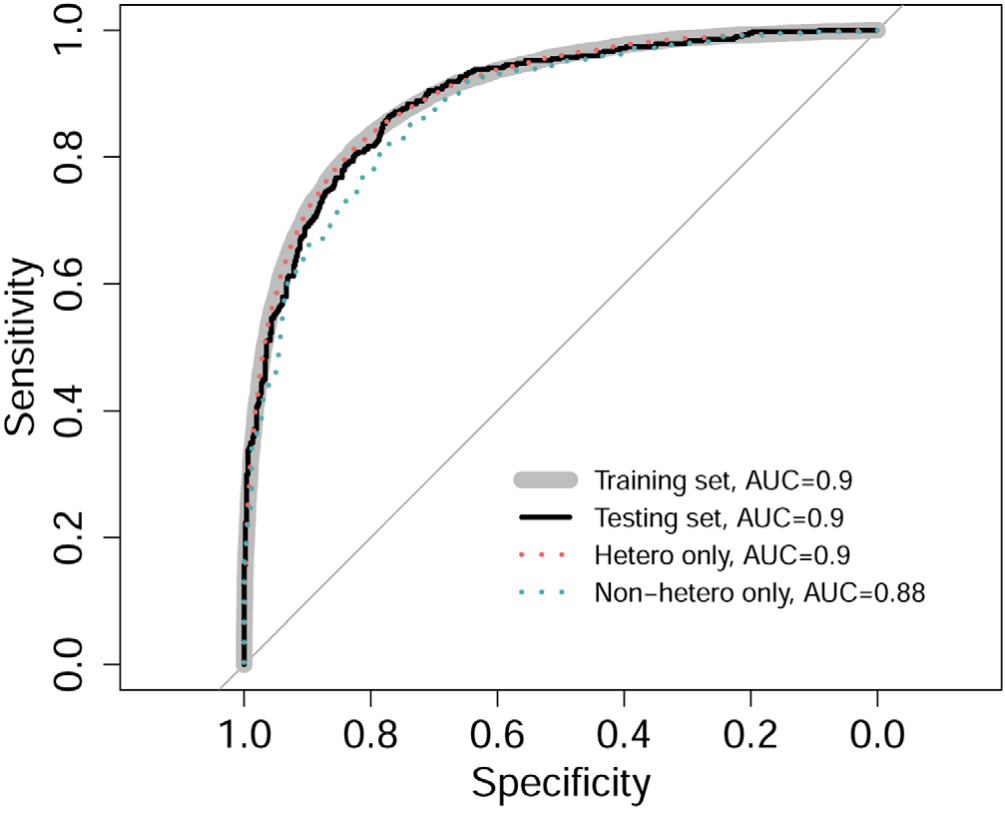
Male/female PLS classification model performance on different sets. Specificity is plotted against sensitivity. AUC, area under the ROC curve. The classifier performed equally well in training and testing sets, as well as in heterosexual individuals only. The performance significantly dropped in non‐heterosexual individuals. Figure 3 shows the magnitude of loadings on LV1 of cortical regions mapped into the brain space (see Supporting Information for loading maps for LV2 and LV3). PLS, partial least squares; LV, latent variable

The PLS model performed equivalently well on the training set (AUC = 0.899) and the smaller testing set (AUC = 0.895; DeLong's test for two ROC curves: D_1026_ = 0.41, *p* = .68; Figure 2) suggesting very good generalization. It also performed similarly in heterosexuals only (AUC = 0.8994; DeLong's tests for two ROC curves D_35624_ = −0.162, *p* = 0.87). As expected, compared with heterosexuals, we observed a significant performance drop (DeLong's test for two ROC curves: D_771.87_ = 1.73, *p* = .041) when testing the model on only non‐heterosexual subjects (AUC = 0.8779; Figure 2). This indicates less pronounced sex‐related brain features in non‐heterosexual individuals. Three LVs were identified as optimal to distinguish between males and females.

Testing a reverse hypothesis using PLS to predict SSB yielded converging results. Although we identified one latent component, which was similar to LV1 from the main analysis with the same sex‐by‐SSB effect (*F*_18,641_ = 8.464, *p* = .0036) and regional loadings profile, the classifier performance predicting SSB based on imaging data was weak (AUC = 0.57).

### 3.3 Analysis of cross‐sex shifts in LVs

**FIGURE 3:**
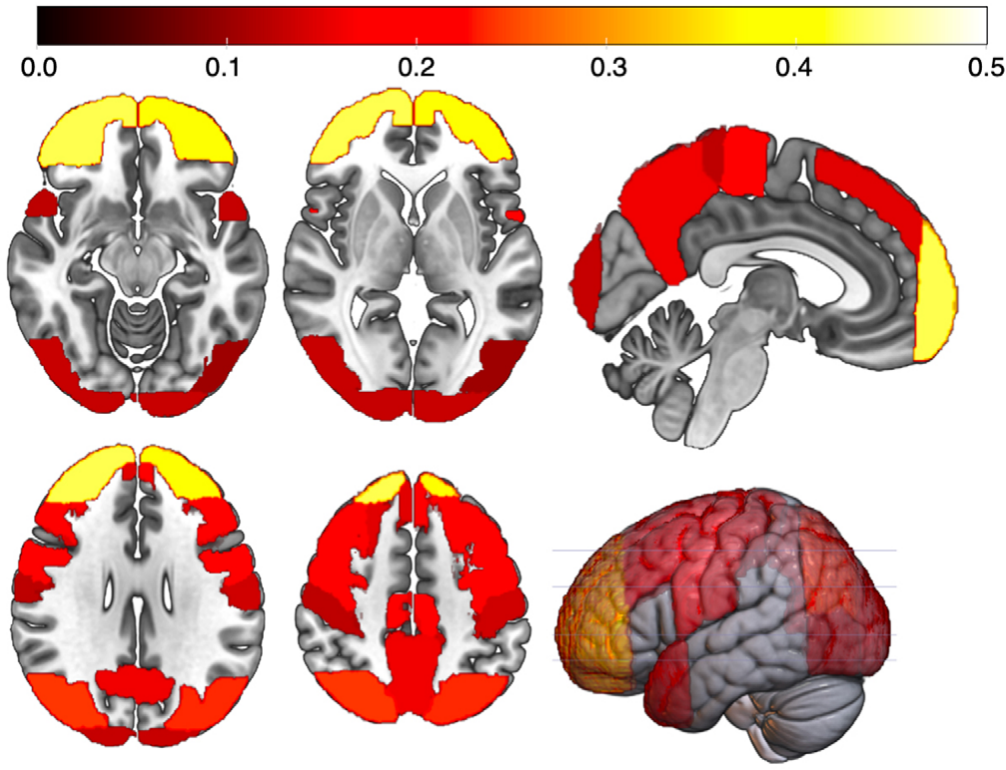
LV1 loadings mapped into the MNI brain space. Color bar represents magnitude of loadings. Near zero loadings (<0.1) are not displayed. Loadings of all subcortical volumes and tract FA‐values were close to zero. FA, fractional anisotropy; LV, latent variable

After Bonferroni correction, a significant sex‐by‐SSB interaction was found for LV1 (Table 2). Corresponding regional loadings are illustrated in Figure 3. This component was also significantly associated with SSB and sex (Table 2). For LV2, there were main effects of sex and SSB, but no sex‐by‐SSB interaction was observed (Table 2). LV3 solely related to sex.

**TABLE 2:** Latent variable (LV) findings

Predictor	LV1	LV2	LV3
Sex	***F*(1, 18,641) = 4,523.78, *p* < .001**	***F*(1, 18,641) = 4,617.14, *p* < .001**	***F*(1, 18,641) = 971.18, *p* < .001**
SSB	***F*(1, 18,641) = 28.74, *p* < .001**	***F*(1, 18,641) = 5.55, p = .018**	*F*(1, 18,641) = 0.04, *p* = .850
Sex‐by‐SSB	***F*(1, 18,641) = 8.57, *p* = .003**	*F*(1, 18,641) = 0.438, *p* = .510	*F*(1,18,641) = 0.2, *p* = .655

*Note*: Statistical results of regression analyses testing the effects of sex, SSB, and sex‐by‐SSB on LV1, LV2, and LV3. Significant findings (p < .05) are shown in bold. For sex‐by‐SSB (variable of interest), p < .017 was considered significant after adjusting for multiple testing (Data S1).

**FIGURE 4:**
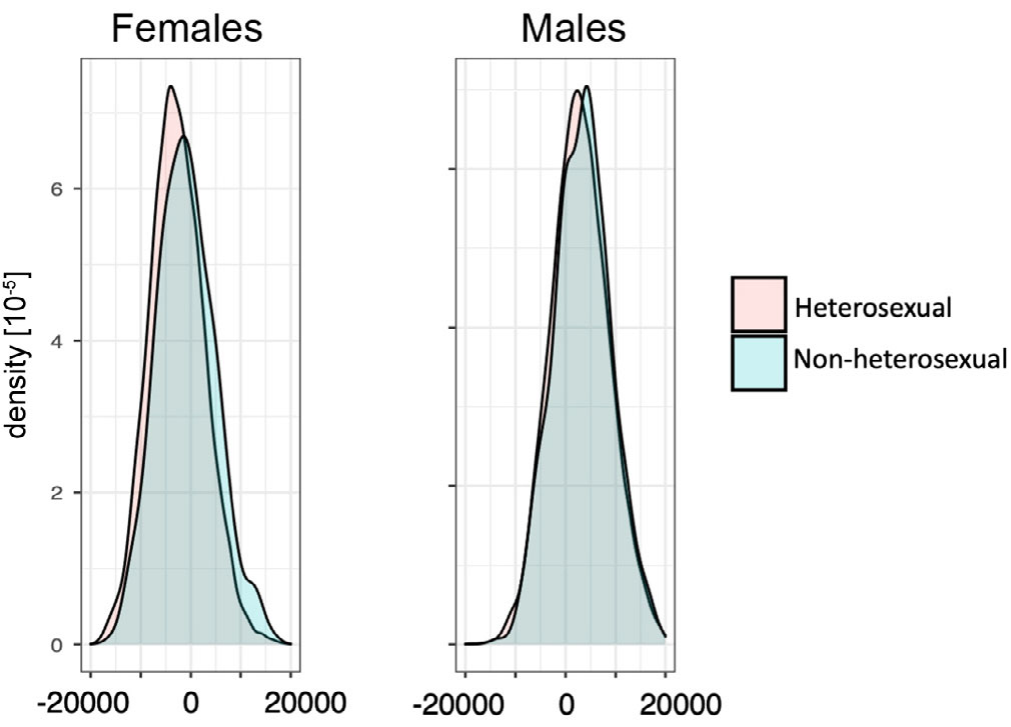
Distributions of LV1‐scores that showed a significant sex‐by‐SSB interaction. Distributions are shown in relation to SSB (heterosexual vs. non‐heterosexual) in females (left) and males (right). LV, latent variable; SSB, same‐sex sexual behavior

Post hoc tests on LVs revealed that males showed positive scores and females showed negative scores (Table 3). Hence, larger values may reflect more male‐like brain patterns. In LV1, heterosexual and non‐heterosexual females differed significantly, with non‐heterosexual females showing larger scores (shifted toward that of males) (Table 3). Heterosexual and non‐heterosexual males did not differ significantly in LV1. Thus, a clearer cross‐sex shift was only observed in females. Figure 4 shows LV1 score distributions for each group.

**TABLE 3:** Latent variable (LV) group means

	Females			Males		
	HeF	nHeF	HeF versus nHeF, *p*	HeM	nHeM	HeM versus nHeM, *p*
**LV1**	**−2,726 ± 5,517**	**−883 ± 5,778**	**<.001**	**2,936 ± 5,895**	**3,510 ± 5,812**	**.057**
LV2	−651 ± 1,348	−812 ± 1,281	.025	730 ± 1,430	638 ± 1,426	.214
LV3	−277 ± 1,247	−291 ± 1,288	.840	308 ± 1,323	338 ± 1,307	.664

*Note*: Means ± *SD* of LVs in each group and results (*p*‐values) of respective post hoc group comparisons. Results for LV1, which displayed the sex‐by‐SSB interaction, are highlighted in bold.

Given the bimodal LV1 distribution in nHeM, we performed an exploratory analysis for interpretational purpose to test the role of potential sub‐groups in nHeM (see Supporting Information for details).

### 3.4 Adjusting for demographic and other potential confounding variables

The significant interaction effect on LV1 was still present after adjusting for any potential confounding variable mentioned above (see Table 1). The interaction effect on LV1 was not significant when adjusting for ICV. This was expected as ICV strongly relates to sex and the phenotype under investigation (see Supporting Information), also indicated by the hypothesized cross‐sex shift in ICV (sex‐by‐SSB effect on ICV: *p* = .001). Hence, correcting for it removes important variance related to the effects of interest, as demonstrated here, and the result when correcting for ICV should be interpreted with caution. Note, when first scaling brain measure to ICV, we observed only minor influences on PLS classification accuracies (AUC_training/testing_ = 0.85/0.86).

### 3.5 Correlations between polygenic scores for SSB and brain imaging outcomes

Regression analyses revealed no association between PS‐SSB scores and LVs. However, in region‐specific analyses, after correcting for multiple testing, we found a significant three‐way (SSB‐by‐sex‐by‐PS‐SSB) interaction in the right middle temporo‐occipital cortex (*p* < .001, *t* = −3.599, *β* = −.040, *F*[8, 18,130] = 300.3, *R* ^2^ = .117). Post hoc analyses for this region revealed a significant sex‐by‐PS‐SSB interaction in non‐heterosexuals (*p* = .001, *t* = −3.219, *β* = −.178; *F*[4, 695] = 21, *R* ^2^ = .108) but no sex‐by‐PS‐SSB interaction in heterosexual individuals (*p* = .342, *t* = 0.950; *β* = .009; *F*[4, 17,434] = 578.3, *R* ^2^ = .117). The correlation between right middle temporo‐occipital cortical volume and PS‐SSB was negative in nHeM (*p* = .030, *t* = −2.181, *β* = −.110) and positive in nHeF (*p* = .018, *t* = 2.369, *β* = .131). There was no correlation in HeM (*p* = .930, *t* = 0.088, *β* = .001) or HeF (*p* = .179, *t* = −1.345, *β* = −.014).

**FIGURE 5:**
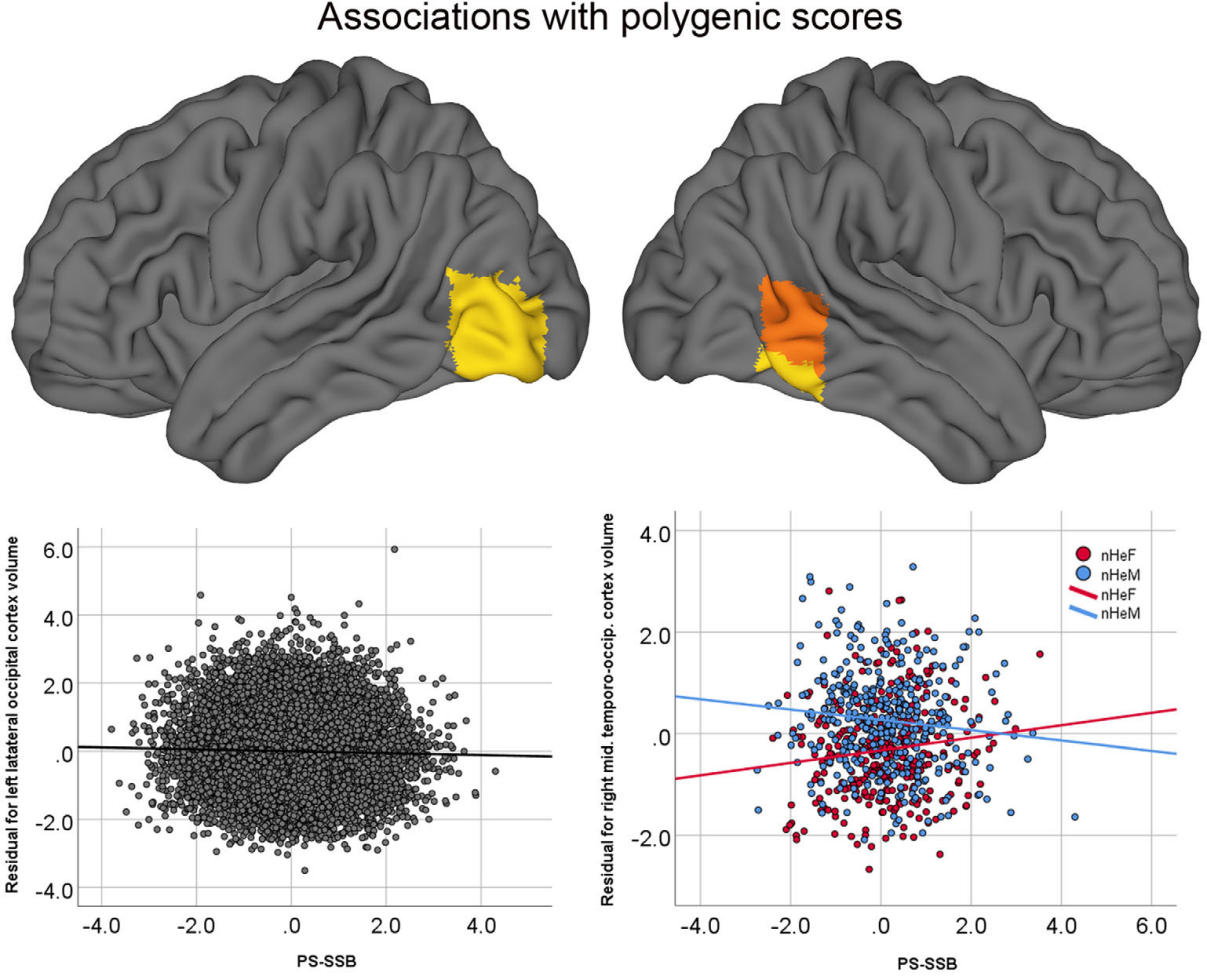
Associations between PS‐SSB and regional cortical volumes. Top panel: Regions for which significant associations with PS‐SSB were observed in the combined cohort (heterosexual and non‐heterosexual individuals together) are shown in yellow. Regions for which significant sex‐by‐PS‐SSB interactions (driven by non‐heterosexual individuals) were found are shown in orange. Bottom panel: Scatter plot showing the association with PS‐SSB and left lateral occipital cortex volume (top left; yellow) in the combined cohort. The association between PS‐SSB and right middle temporo‐occipital cortex (top right; orange) is shown on the bottom right displaying the sex‐by‐PS‐SSB interaction observed in nHeM and nHeF. SSB, same‐sex sexual behavior; PS‐SSB, polygenic score for SSB

In addition, testing for a main effect of PS‐SSB on cortical volume, we found a negative association between PS‐SSB and both volume of left lateral inferior occipital cortex (main effect of PS‐SSB: *p* < .001, *t* = −3.716, *β* = −.026; *F*[3, 18,135] = 592.625, *R* ^2^ = .089) and volume of the right inferior temporo‐occipital cortex (*p* = .0017, *t* = −3.132, *β* = −.022; *F*[3, 18,135] = 1,017, *R* ^2^ = 0.144) independent of sex; relationships were similar in males (occipital: *p* = .009, *t* = −2.604, *β* = −.028; temporal: *p* = .018, *t* = −2.365, *β* = −.025) and females (occipital: *p* = .008, *t* = −2.640, *β* = −.026; temporal: *p* = .040, *t* = −2.1, *β* = −.021). Figure 5 displays these regions in brain space and corresponding scatter plots. Results did not change when adjusting for any potential confounder variables listed in Table 1.

No significant relationships between PS‐SSB and subcortical volumes or FA‐values were observed, with the exception of one significant three‐way interaction for FA in the left corticospinal tract (*p* = .005, *t* = −2.835, *β* = −.033, *F*[8, 18,130] = 66.695, *R* ^2^ = .029). Follow‐up analyses revealed an association between PS‐SSB and FA in nHeM only (*p* = .015, *t* = −2.368, *β* = −.121). See Data S1 for results in other brain areas.

### 3.6 Cross‐sex shifts in DTI‐based FA‐values and volumetric measures (secondary analyses)

**FIGURE 6:**
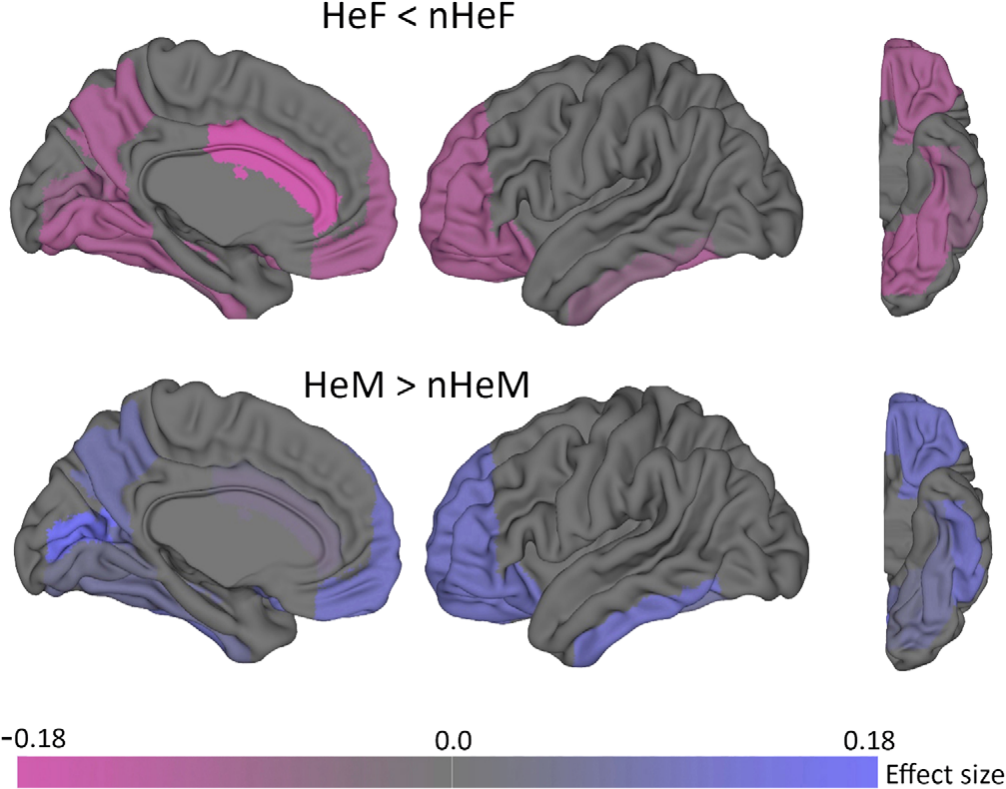
Effect size of same‐sex sexual behavior (SSB)‐related cross‐sex shifts in brain structure. Color shades correspond to the observed effect size (Cohen's *d*) obtained from pairwise group comparisons (heterosexual vs. non‐heterosexual) in secondary post hoc analyses (univariate region of interest approach; see Supporting Information for details). Corresponding numerical values are given in Table S3. Whole brain data are provided in Data S2. Top: heterosexual females (HeF) versus non‐heterosexual females (nHeF). Bottom: heterosexual males (HeM) vs. non‐heterosexual males (nHeM). Negative effect sizes (magenta) reflect HeF < nHeF patterns (nHeF shifted toward HeM). Positive effect sizes (blue) reflect HeM > nHeM patterns (nHeM shifted toward HeF). Overall, a HeF < nHeF<nHeM<HeM pattern was observed. In males, the largest effect was found in calcarine sulcus, whereas in females the largest effect was observed in the anterior cingulate cortex (ACC). The scheme reflects bilateral findings

Secondary ROI analyses revealed significant sex‐by‐SSB interactions in the calcarine, prefrontal cortex, precuneus, inferior temporal cortex, and thalamus (Table S2). SSB‐related effect sizes are shown in Figure 6. No sex‐by‐SSB interactions were observed for DTI measures. See Supporting Information for details.

## 4 DISCUSSION

In this large‐scale study on SSB, we used brain imaging phenotypes from two imaging modalities and a multivariate classification algorithm to extract independent brain covariance patterns related to sex. We then tested for SSB related cross‐sex shifts in such patterns. For the first time, we also examined whether polygenic scores for SSB relate to brain imaging phenotypes.

Our results showed that the PLS classifier was effective in classifying males and females, and that patterns important for classification were less pronounced in non‐heterosexual individuals, indicative of a cross‐sex shift. The analysis of LVs demonstrated that one (LV1) displayed a sex‐by‐SSB interaction. This interaction remained following adjustment for potential confounding variables, including psychiatric diagnoses and victimization experiences, and was driven by the fact that nHeF showed larger LV1 scores than HeF. Since males showed the largest LV scores, this indicates an SSB‐related cross‐sex shift in multivariate brain patterns predominantly in females. This shift in LV1 was not observed in males, which could potentially arise because SSB‐related differences in males might have less of a covarying nature, regionally differ, be more focal, or less pronounced (smaller effect size) compared to females, as indicated by secondary univariate analyses (Figure 6). However, these differences could also be explained by the fact that the SSB measure does not capture all aspects of sexual orientation. While SSB correlates highly with other components of sexual orientation, nHeF and nHeM in our sample may differ in other components such as sexual attractions, sexual identity labels, or romantic attractions (J. M. Bailey et al., 2016). Hence, we cannot exclude the presence of sub‐groups among non‐heterosexual individuals. In line with that notion, in an explorative analysis excluding individuals with one or two reported lifetime same‐sex partners (see Supporting Information), the peak of the LV1 distribution in nHeM was shifted toward smaller values (the mean of females), indicating that a sub‐group of nHeM (e.g., those with more same‐sex partners) may show a more female‐like multivariate brain pattern. However, this effect requires further investigation. Nevertheless, our findings suggest sexuality‐related variation in multivariate brain data, supporting the utility of data‐driven classification and that multivariate pattern analyses are effective at identifying such associations on group level, at least in females.

Our neuroanatomical findings support a number of previous small‐scale reports of sexual orientation‐related differences (Abé et al., 2014; Abé et al., 2018; Manzouri & Savic, 2018a, 2018b; Ponseti et al., 2007; Savic & Lindström, 2008) in that they indicate SSB‐related cross‐sex shifts in brain imaging phenotypes. Intriguingly, the calcarine sulcus (part of the visual cortex) appears to be the most consistently reported structure showing sexual orientation‐related differences (Abé et al., 2014; Abé et al., 2018; Manzouri & Savic, 2018b), which is consistent with results from our secondary univariate analyses (ROI approach: Figure 6, and whole brain analysis: Data S2). We did not replicate sexual orientation differences in the anterior cingulate cortex (Manzouri & Savic, 2018a, 2018b) and hippocampus (Abé et al., 2014) in males. Cross‐sex shifts in brain data are also consistent with a large body of empirical findings demonstrating cross‐sex shifted patterns of gender‐related behavior, cognitive ability (in tasks that typically differ between the sexes), and certain personality traits (Allen & Robson, 2020; Bailey et al., 2016; Li et al., 2017; Rieger et al., 2008; Xu et al., 2017). However, there is considerable overlap in the distribution of LV‐scores between the groups, and the magnitude of the effects for SSB‐related brain differences seem smaller than those reported for the aforementioned behavioral traits. Notably, effect sizes for SSB‐related differences in cortical volumes were also smaller than those of sex differences (Table S3, Data S2).

The imaging variables that loaded most strongly on LV1 (displaying the sex‐by‐SSB interaction) were measures of regional volumes in prefrontal, parietal, and occipital (including visual) cortices. In the context of SSB, the visual cortex is involved in visual perception and processing of sexual stimuli (Georgiadis & Kringelbach, 2012). Prefrontal areas are involved in the integration of sensory information and reward‐value representation of sexual stimuli (Georgiadis & Kringelbach, 2012). Together with the precuneus, involved in self‐referential processes (Cavanna & Trimble, 2006), these areas are also recruited during visuo‐spatial processing and selective visual attention (Cavanna & Trimble, 2006; Georgiadis & Kringelbach, 2012; Paneri & Gregoriou, 2017; Posner & Gilbert, 1999). However, this study does not allow conclusions about causality or the brain regions' functional involvement. It requires further testing how differences in brain structure relate to SSB. Note that although volumes of those brain regions that tended to successfully predict group membership largely overlap with those previously reported in other studies, in contrast to direct group comparisons in univariate analyses, PLS results should not necessarily be interpreted as evidence of structural differences between the groups, but rather as generalized covariance patterns in the brain data that discriminate between them. Another important finding is that while LV1 appeared to capture the hypothesized cross‐sex shift, LV2 appeared to capture a main effect of SSB. This may indicate that SSB‐related multivariate brain patterns may exist that do not follow a cross‐sex shift and are similar in both nHeM and nHeF (regardless of sex). It is also noteworthy that cortical volumetric measures showed the highest loadings, whereas those of subcortical structures and DTI‐based FA values were close to zero, indicating that sex‐related brain phenotype variation may be more pronounced in gray matter than white matter or subcortical measures.

The causes of sexual orientation‐related differences in brain structure are as yet unknown. Both genetic and non‐genetic factors have been proposed to play a role, with the most prominent hypothesis involving prenatal androgen influences (Bailey et al., 2016; Kevin, Khytam, & David, 2018). Genetic influences are modest based on existing twin models and molecular genetic studies (Bailey et al., 2000; Bailey et al., 2016; Ganna & Verweij, 2019; Langstrom et al., 2010) and are almost certainly polygenic in nature (Ganna & Verweij, 2019). Here, we investigated genetic influences on brain phenotypes by testing the associations between polygenic scores for SSB (PS‐SSB) and brain imaging phenotypes. Whereas PS‐SSB did not seem to predict multivariate brain patterns (LVs), we found that PS‐SSB was associated with cortical volumes in individual brain regions. These associations were observed mainly in lateral occipital and temporo‐occipital cortex. In lateral occipital cortex, higher PS‐SSB was associated with lower volumes in both males and females. In temporo‐occipital cortex, higher PS‐SSB was associated with lower cortical volumes in nHeM and larger volumes nHeF. These findings tentatively indicate that genetic factors related to SSB are associated with variation in some cortical structures and that a higher genetic predisposition to SSB has the opposite effect on cortical volume in males and females who reported SSB. These associations were small and PS‐SSB explained little of the variance in brain structure. Notably, we did not find significant genetic correlations in complementary analyses linking previously published SSB and brain phenotype GWASs (Elliott et al., 2018; Ganna & Verweij, 2019). Therefore, these genetic associations should be treated with caution, and additional factors are likely to explain brain variation associated with human sexuality. Mechanisms responsible for how genetic factors influence brain structure, function, and in turn behavior are complex and multi‐factorial. Given the general limitations of the applied methodology (see below), these cannot be derived from this study. We also want to note that, given the wide and overlapping range of LVs and PS‐SSB, as well as the weak classification performance when solely predicting SSB (AUC = 0.57), the present results cannot be used to predict an individual's sexual orientation based on genetic or neuroimaging data.

## 5 CONCLUSION

The present study is the largest neuroimaging investigation and the first imaging‐genetics study on SSB to date. Our study demonstrates a structural neurobiological association with SSB, albeit with small effect sizes, and indicates a possible genetic influence on brain structure.The neural correlates of SBB were unrelated to mental health disparities and experience of victimization. The observed SSB‐related mental health disparities, however, highlight the importance of improving health‐care provision aimed at reducing the burden of mental health problems faced by sexual minority groups.

### 5.1 Strengths and limitations

The present study on SSB had several strengths including the largest neuroimaging dataset studied to date, comprising volumetric and DTI data, the well‐characterized biomedical cohort, measurement of same‐sex behavior at several time points, and assessment of potential confounding variables. Our study is also the first to address a significant gap in scientific research by linking the known genetic influences on SSB to brain phenotypes. However, there are several important limitations to note.

Although the main methodology applied here (PLS classification) enables the investigation of multivariate patterns, it does not provide information on group differences in individual brain regions. We have reported results from secondary tests to provide such complementary information.

We observed cross‐sex shifts in both ICV and individual brain structures. However, results from both multivariate and mass‐univariate analyses indicated that the effects were not global, and changes in ICV were unlikely driving the regional cross‐sex shifts. Future studies testing whether cross‐sex shifts in ICV and regional brain structure underlie shared or different biological processes are warranted.

Although nHe showed elevated numbers of psychiatric disorders, which is in line with previous studies (Abé et al., 2018; Branstrom, 2017; Frisell et al., 2010; Sandfort et al., 2001), we were able to control for it and our findings were not influenced by such mental health disparities, i.e., brain alterations associated with psychiatric disorders. Likewise, controlling for self‐reported experiences of victimization did not change our results.

Some of the analyses performed here (e.g., those adjusting for confounders) were performed on subsamples as not all participants provided complete data for the follow‐up test variable. Furthermore, the UK Biobank sample comprises a volunteer sample and the sample is older than the general population, thus, may not be fully representative. Self‐reported ethnic background was mainly “white” and future studies on different populations are warranted.

Moreover, the UK Biobank does not assess other important indicators of sexual orientation, such as sexual attraction or identity labels. However, sexual behavior measures are commonly used in large cohort studies because of their ease of administration, their unambiguous nature, and their tendency to be free from social identity labeling. SSB correlates highly with attraction and other components of sexual orientation (Abé et al., 2018; Bailey et al., 2016). The prevalence of SSB in this sample was similar to the population prevalence of non‐heterosexuality in Western samples (1–6%) when based on identity (Gates, 2011). However, future studies should quantify sexual orientation using multiple domains.

We investigated cortical volume, which is a function of cortical thickness and surface area. Future investigations of these measures together with functional and other DTI‐based phenotypes (e.g., diffusivity) can provide additional insights into the neural characteristics of SSB. In addition, polygenic scores are cumulative measures reflecting an individual's genetic predisposition to SSB. However, it was not the aim of this study to identify detailed genetic factors associated with brain structural variation.

Finally, polygenic scores were computed based on a GWAS including both males and females, and the scores reflect SSB‐related alleles that were shared between sexes. Although our findings suggest that such common genetic factors are associated with brain structure, whether SSB‐related genetic factors that are specific for each sex have additional effects on brain structural variation remains to be investigated.

## CONFLICT OF INTERESTS

The authors declare no conflict of interest.

## AUTHOR CONTRIBUTIONS

Christoph Abé was principal investigator. Christoph Abé and Qazi Rahman were responsible for the study concept and design. Alexander Lebedev and Christoph Abé performed the majority of analyses, and created figures and tables. Lina Jonsson performed LD score regressions and Ruyue Zhang performed GWAS and polygenic risk scoring, which was guided by Sarah E. Bergen, and created corresponding tables and figures. Christoph Abé wrote the first draft of the manuscript and Supporting Information. Alexander Lebedev, Qazi Rahman, and Ruyue Zhang, contributed substantially to the writing of the manuscript. Sarah E. Bergen, Martin Ingvar, and Mikael Landén provided important input for main text and supplements. All authors were involved in the interpretation of data, provided critical revision of the manuscript for important intellectual content, and approved the manuscript for submission.

## ETHICS STATEMENT

UK Biobank has Research Tissue Bank (RTB) approval from its governing Research Ethics Committee (REC), as recommended by the National Research Ethics Service (NRES; reference 11/NW/0382). The authors assert that all procedures contributing to this work comply with the ethical standards of the relevant national and institutional committees on human experimentation and with the Helsinki Declaration of 1975, as revised in 2008.

## Supporting information


**Appendix** **S1**: Supporting information

**Appendix** **S2**: Supporting information

**Appendix** **S3**: Supporting information

## Data Availability

Access to individual data used in the analysis can be obtained through formal application with UK Biobank (www.ukbiobank.ac.uk).
