# Supplementary material for: Cross‐sex shifts in two brain imaging phenotypes and their relation to polygenic scores for same‐sex sexual behavior: A study of 18,645 individuals from the UK Biobank
Source: Hum Brain Mapp. 2021 Feb 26;42(7):2292–304. doi: 10.1002/hbm.25370 (PMC8046142; doi:10.1002/hbm.25370)
Supplement: Supplementary file 1 — Appendix S1: Supporting information [file HBM-42-2292-s002.docx]

**Supporting Information for**

**Cross-sex shifts in two brain imaging phenotypes and their relation to polygenic scores for same-sex sexual behavior: A study of 18,645 individuals from the UK Biobank.**

Christoph Abé^1^*, Alexander Lebedev^1^, Ruyue Zhang^2^, Lina Jonsson^3,4^, Sarah E. Bergen^2^, Martin Ingvar^1^, Mikael Landén^2,3^, Qazi Rahman^5^

^1^Department of Clinical Neuroscience, Karolinska Institutet, Stockholm, Sweden.

^2^Department of Medical Epidemiology and Biostatistics, Karolinska Institutet, Stockholm, Sweden.

^3^Department of Psychiatry and Neurochemistry, Institute of Neuroscience and Physiology, Sahlgrenska Academy, University of Gothenburg, Gothenburg, Sweden.

^4^Department of Pharmacology, Institute of Neuroscience and Physiology, Sahlgrenska

^5^Department of Psychology, Institute of Psychiatry, Psychology, and Neuroscience, King’s College London, London, SE1 8AF, UK.

*Correspondence: Christoph Abé, PhD, Assistant Professor, Department of Clinical Neuroscience, Karolinska Institutet, Nobels väg 9, 17177 Stockholm, Sweden, Phone: +468 52483265, Email: christoph.abe@ki.se.

**Brain image acquisition and processing**

Structural and diffusion tensor imaging (DTI) MRI data were acquired on a single scanner (Siemens Skyra 3T) equipped with a standard Siemens 32-channel head coil, see ([Miller et al., 2016](#_ENREF_15)) and URLs:

<http://biobank.ctsu.ox.ac.uk/crystal/refer.cgi?id=2367>

http://biobank.ctsu.ox.ac.uk/crystal/refer.cgi?id=1977.

During the period of MRI data acquisition, no significant changes were made to scanner hard- or software. Details on protocol phases, upgrades, image acquisition and processing are described in the UK Biobank Brain Imaging Documentation available at

http://biobank.ndph.ox.ac.uk/showcase/showcase/docs/brain_mri.pdf ([Alfaro-Almagro et al., 2018](#_ENREF_3); [Miller et al., 2016](#_ENREF_15)). Our study made use of pre-processed image data and imaging-derived phenotypes (IDPs) generated by an image-processing pipeline developed and run on behalf of UK Biobank ([Alfaro-Almagro et al., 2018](#_ENREF_3)). In brief, T1 weighted 3D MPRAGE images (resolution: 1x1x1 mm^3^, matrix size: 208x256x256) were acquired and processed using fMRIB Software Library (FSL) tools (http://www.fmrib.ox.ac.uk/fsl) by the UK Biobank brain imaging team. Processing included defacing, skull stripping, bias field correction, and cerebrospinal fluid (CSF), grey and white matter tissue segmentation using the functions BET, FLIRT, FNIRT, FIRST, and FAST. Due to the very large sample size, a systematic visual quality control was not feasible and therefore not performed by the UK Biobank team ([Alfaro-Almagro et al., 2018](#_ENREF_3)). However, if artefacts, e.g. due to head motion, were identified during MRI scanning, image acquisition was repeated. The FAST segmentation allowed the automated parcellation of cortical regions of interest (ROIs) using the Harvard-Oxford cortical atlas

(https://fsl.fmrib.ox.ac.uk/fsl/fslwiki/Atlases). Subcortical segmentation was performed using FIRST. The main outcome measures (IDPs) investigated were volumes of cortical and subcortical regions defined in subjects’ native space. Processing of DTI data (resolution: 2x2x2 mm, matrix size: 104x104x72, 50xb=1000, 50xb=2000, 2x50 directions) included gradient distortion correction, motion and eddy current correction. BEDPOSTx was used to model within-voxel multi-fiber tract orientation, followed by probabilistic tractography (modelling crossing fibers) using PROBTRACKx. FSL was used to map 27 major white matter tracts from which average fractional anisotropy (FA) was extracted.

**Psychiatric morbidity**

UK Biobank provided hospital records obtained through linkage to external medical providers, providing diagnoses a participant has had recorded across all their hospital inpatient records (Data-Field 41202). Given the elevated rate of psychiatric disorders in non-heterosexual populations ([Abé et al., 2018](#_ENREF_2); [Branstrom, 2017](#_ENREF_4); [Frisell, Lichtenstein, Rahman, & Langstrom, 2010](#_ENREF_7); [Plöderl & Tremblay, 2015](#_ENREF_17); [T. Sandfort, Graaf, Have, Ransome, & Schnabel, 2014](#_ENREF_19); [T. G. Sandfort, de Graaf, Bijl, & Schnabel, 2001](#_ENREF_20)), we tested the influence of the following common mental disorders on our main results (diagnoses were coded according to the International Classification of Diseases, ICD-10): Bipolar Disorder (BD, category F31), Manic episode (ME, F30), Major depressive disorder, recurrent (MDD, F33), Major depressive disorder, single episode (MDDs, F32), Attention Deficit Hyperactivity Disorder (ADHD, F90), substance use-related disorders (SUD, F10-19), alcohol use-related disorder (AUD, F10), phobic anxiety disorders (ANX, F40), other anxiety disorder including panic and general anxiety disorders (PAN, F41), obsessive-compulsive disorder (OCD, F42), stress and adjustment disorders (STR, F43), schizophrenia, schizotypal, delusional, and other non-mood psychotic disorders (SCZ, F20-29), personality disorders (PD, F60), intellectual disabilities (ID, F70-79), eating disorders (ED, F50), and pervasive developmental disorders including autistic disorder (ATD, F84). Each of these were coded as present or not (binary). In addition to separate diagnoses, we created a combined binary discrete variable coding for the presence of any psychiatric disorder.

**Table S1**. ICD-10 psychiatric diagnoses breakdown.

| **ICD-10 diagnoses % (n)** | **HeM** | **HeF** | **nHeM** | **nHeF** | **p** |
| --- | --- | --- | --- | --- | --- |
| BD | 0.1 (7) | 0.1 (13) | 0.3 (1) | 0 (0) | ns |
| ME | 0.0 (3) | 0.1 (5) | 0.0 (0) | 0.0 (0) | ns |
| MDDs | 1.2 (104) | 2.0 (189) | 2.3 (9) | 3.3 (11) | < 0.001 |
| MDD | 0.1 (5) | 0.1 (11) | 0 (0) | 0.3 (1) | ns |
| ADHD | 0 (0) | 0 (0) | 0 (0) | 0 (0) | - |
| SUD | 2.0 (171) | 1.2 (112) | 5.1 (20) | 1.2 (4) | < 0.001 |
| AUD | 0.9 (72) | 0.3 (24) | 2.3 (9) | 0 (0) | < 0.001 |
| ANX | 0.0 (2) | 0.1 (11) | 0.3 (1) | 0.0 (0) | ns |
| PAN | 0.6 (48) | 1.0 (99) | 2.0 (8) | 1.8 (6) | < 0.001 |
| OCD | 0.0 (1) | 0.0 (2) | 0.0 (0) | 0.0 (0) | ns |
| STR | 0.0 (4) | 0.1 (12) | 0.0 (0) | 0.6 (2) | 0.007 |
| SCZ | 0.1 (8) | 0.1 (5) | 0.5 (2) | 0.6 (2) | < 0.001 |
| PD | 0.0 (3) | 0.0 (3) | 0.8 (3) | 0.3 (1) | < 0.001 |
| ID | 0.0 (0) | 0.0 (0) | 0.0 (0) | 0.0 (0) | - |
| ATD | 0.0 (1) | 0.0 (1) | 0.0 (0) | 0.0 (2) | ns |
| ED | 0.0 (0) | 0.0 (1) | 0.0 (0) | 0.0 (0) | ns |

Prevalence for specific psychiatric diagnoses in % and number of individuals (in parenthesis) are listed. Results of χ^2^-tests are given.

**Figure S1. Three-model-comparison (classification).**


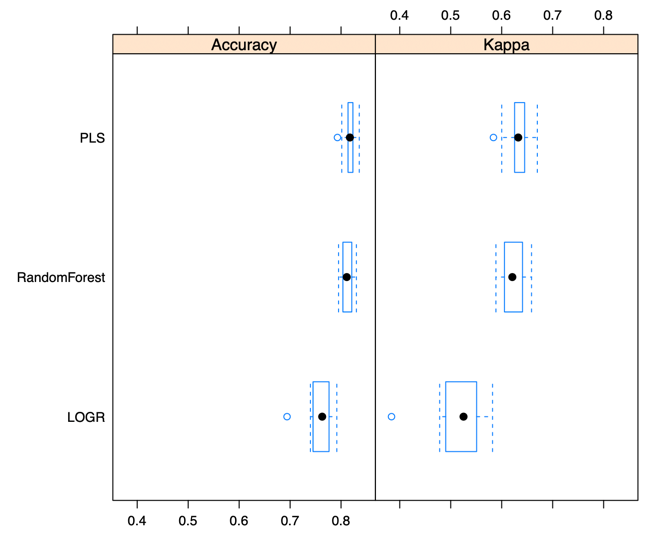


PLS model diagnostics. None of the alternative algorithms outperformed the main (PLS) model in terms of accuracy and kappa values, reflecting performance of a classifier against chance-level predictions Kappa = [total accuracy – random accuracy)]/[1- random accuracy]). Therefore, we continued subsequent analyses using PLS, as intended.

**Figure S2. Determination of number of components (LVs)**


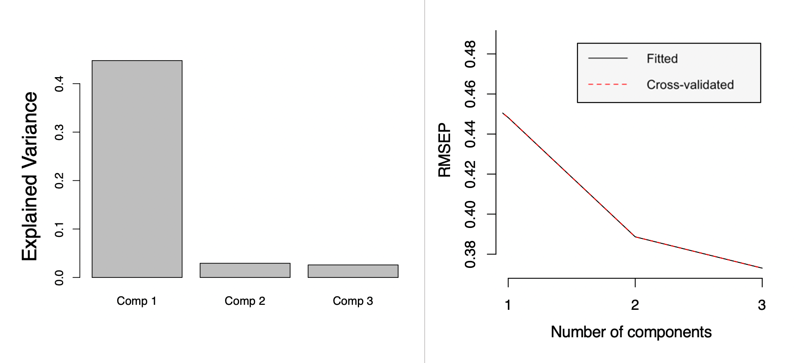


PLS model diagnostics. Optimal number of components (LVs) was estimated at 3 through minimizing root mean squared error prediction (RMSEP).

**Figure S3: LV2 loadings mapped into the MNI brain space.**


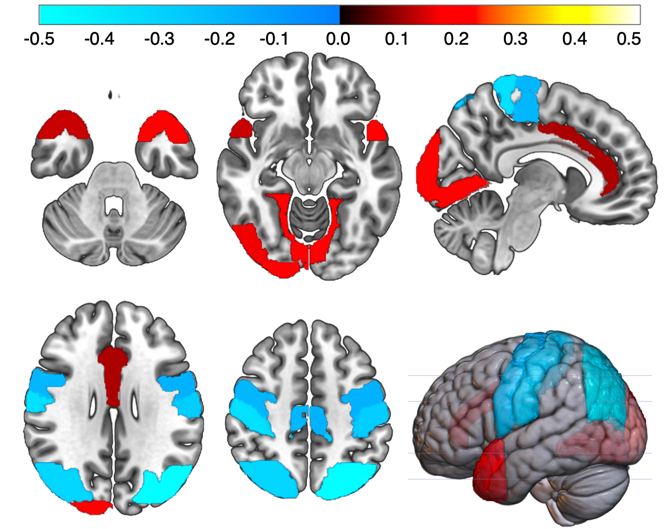


Color bar represents magnitude of loadings. Near zero loadings (<0.1) are not displayed. Loadings of all subcortical volumes and tract FA-values were close to zero. LV2 showed a significant main effect of SSB.

**Figure S4: LV3 loadings mapped into the MNI brain space.**


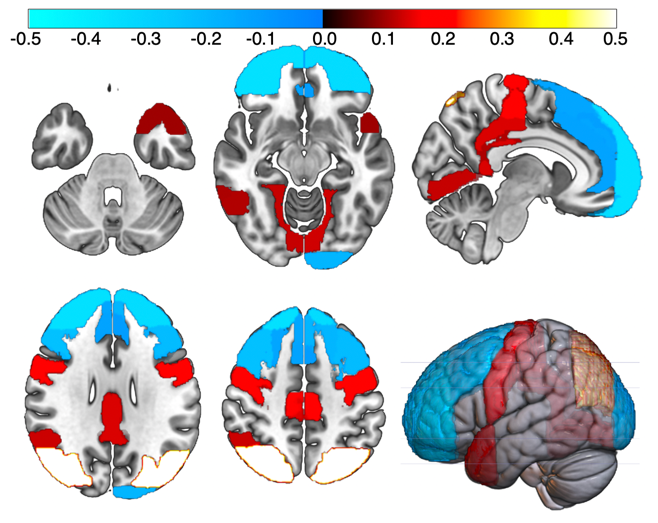


The color bar represents the magnitude of loadings. Near zero loadings (<0.1) are not displayed. Loadings for all subcortical volumes and tract FA-values were close to zero. LV3 did not show a sex-by-SSB interaction or main effect of SSB, only a main effect of sex.

**Independent effects of PS-SSB and SSB on brain volumes.**

In regression models testing for a main effect of PS-SSB on cortical volume, we added SSB as additional covariate. The negative association between PS-SSB and both volume of left lateral inferior occipital cortex (main effect of PS-SSB: p<0.001, t=-3.711, beta=-.026; F(4,18134)=444.449, R^2^=0.089) and volume of the right inferior temporo-occipital cortex (p=0.0019, t=-3.104,beta=-.021; F(4,18134)=763.556, R^2^=0.144) remained when controlling for self-reported SSB. In both models, SSB was non-significant predictor (p=0.892 and p=287).

**Selection of brain regions of interest (hypothesis-driven ROI approach)**

In an hypothesis-driven region of interest (ROI) approach, we tested if cross-sex shifts appear in midline brain structures as recently suggested by ([Manzouri & Savic, 2018b](#_ENREF_14)). Given the limited power in previous studies, we investigated both, regions that previously indicated cross-sex shifts and regions that showed sexual orientation-related differences *per se*. We selected the following cortical brain regions of interest (ROIs) a-priori: Cuneus, lingual, and calcarine cortex (medial occipital/visual cortex), these being midline structures most consistently reported ([Abé, Johansson, Allzen, & Savic, 2014](#_ENREF_1); [Abé et al., 2018](#_ENREF_2); [Manzouri & Savic, 2018b](#_ENREF_14)). We further included the medial temporal cortex (parahippocampal and fusiform) ([Ponseti et al., 2007](#_ENREF_18)) and the orbitofrontal cortex (OFC) ([Abé et al., 2014](#_ENREF_1)), the inferior temporal cortex ([Abé et al., 2014](#_ENREF_1); [Ponseti et al., 2007](#_ENREF_18)) (adjacent to medial temporal cortex), anterior cingulate (ACC) ([Manzouri & Savic, 2018a](#_ENREF_13), [2018b](#_ENREF_14)), and the precuneus ([Manzouri & Savic, 2018a](#_ENREF_13), [2018b](#_ENREF_14)). These eleven ROIs were constructed using parcellations based on the Harvard-Oxford cortical atlas by combining (summing) regional cortical volumes as follows: Calcarine (Supra- and Intracalcarine Cortex), lingual (Lingual Gyrus), cuneus (Cuneal Cortex), PFC/OFC (Frontal Medial, Frontal Orbital, and, since it contained a large portion of OFC, Frontal Pole), precuneus (Precuneous Cortex), inferior temporal cortex (Inferior Temporal Gyrus, anterior and posterior division, and temporooccipital part), fusiform (Temporal Fusiform Cortex, anterior and posterior division, Temporal Occipital Fusiform Cortex, Occipital Fusiform Gyrus), parahippocampus (Parahippocampal Gyrus, anterior and posterior division), and ACC (Cingulate Gyrus, anterior division). Subcortical ROIs included in this analysis were thalamus and hippocampus ([Abé et al., 2014](#_ENREF_1)). As we had no hypothesis regarding regional lateralization patterns, we summed ROIs from left and right hemispheres to bilateral measures.

Note, we also provide results for all 110 individual cortical and subcortical regions separating left and right hemispheres in exploratory whole brain analyses, which was performed for completeness (Data S2).

**Analysis of cross-sex shifts in tract-based FA and volumetric measures**

*Methods*

To test the hypothesis that FA-values and regional brain volumes of nHeM are shifted towards that of HeF, and that of nHeF towards that of HeM, we tested for a sex-by-SSB interaction on brain measures using Multivariate Analysis of Covariance (MANCOVA) followed by individual univariate analyses of covariance (ANCOVAs) with brain measures as dependent variables. Additional main effects included were age, sex, and SSB. Within each modality, significance levels for follow-up ANCOVAs were adjusted for multiple testing using Bonferroni’s Dubey Armitage-Parmar/Sidak’s adjustment of α-level considering the number of tests (number of white matter tracts or number of brain areas) and the inter-correlation between the dependent variables ([Sankoh, Huque, & Dubey, 1997](#_ENREF_21)).

Note, more participants provided structural MRI than DTI data, hence, sample size for volumetric analyzes (9,353 HeM, 10,387 HeF, 455 nHEM, and 368 nHeF) was slightly larger than in FA analyses.

*Results (volumetric ROIs)*

MANCOVA revealed as significant effect of sex-by-SSB interaction on the combined dependent variable (F(11, 20548)=2.41, p=0.005, Wilks' Λ = 0.999). There was a significant main effect of sex (F(11, 20548)=142.30, p<0.001, Wilks' Λ = 0.929), SSB (F(11, 20548)=1.91, p=0.033, Wilks' Λ = 0.999) and age on scan day (F(11, 20548)=530.97, p<0.001, Wilks' Λ = 0.779). Follow-up tests via ANCOVA for each ROI are given in Table S2. Estimated marginal means and descriptives for each group (HeM, nHeM, HeF, nHeF) are shown in Table S3. Quantified effect sizes (Cohen’s *d*) and relevant post-hoc group comparisons revealed the hypothesised cross-sex shift pattern: Males showed larger regional volumes than females. All male vs. female comparisons (regardless of sexual orientation) were significant (p<0.001). HeM also showed larger volumes than nHeM, and HeF showed lower volumes than nHeF (Table S3). Overall, we observed a HeF < nHeF < nHeM < HeM pattern. To visualize the SSB-related cross-sex shifts, effect sizes and the direction of the effects were colour coded and mapped on a cortical surface (Figure 6, main text). Overall, the ROI results remained robust when controlling for potential confounders. See Data S2 for details (and exceptions).

**Table S2: Regional sex-by-SSB interactions (ROI analysis)**

| **Regions** | **F(20558, 1)** | **p-value** |
| --- | --- | --- |
| Calcarine* | 15.126 | <0.001* |
| Prefrontal/orbitofrontal (PFC/OFC)* | 10.621 | 0.001* |
| Precuneus* | 7.586 | 0.006* |
| Inferior temporal* | 7.038 | 0.008* |
| Thalamus* | 5.976 | 0.015* |
| Fusiform | 5.841 | 0.016 |
| Lingual | 4.099 | 0.043 |
| Anterior cingulate (ACC) | 4.037 | 0.045 |
| Cuneus | 2.104 | 0.147 |
| Parahippocampus | 0.311 | 0.577 |
| Hippocampus | 0.082 | 0.775 |

Statistical results and significance of the interaction term for each tested ROI are given. Inter-correlation of all 11 regions in the total sample was r=0.48, leading to an adjusted alpha level = 0.015. Regions that survive this correction are indicated by *. Results for each individual brain region (exploratory whole brain analysis) are provided in Data S2.

**Table S3: Post-hoc pairwise group comparisons (cross-sex shifts within sexes)**

| **Region** | **HeM**  **(mean ± SD)** | **HeF**  **(mean ± SD)** | **nHeM**  **(mean ± SD)** | **nHeF**  **(mean ± SD)** | **HeM vs. HeF**  **(sex diff.)** | **HeM vs. nHeM**  **(male )** | **HeF vs. nHeF (female )** | **Pattern** |
| --- | --- | --- | --- | --- | --- | --- | --- | --- |
| Calcarine* | 6974 ± 1371 | 6219 ± 1199 | 6731 ± 1276 | 6326 ± 1231 | **p < 0.001**  **ES = 0.59**  **(HeM>HeF)** | **p < 0.001**  **ES = 0.18**  **(HeM>nHeM)** | p = 0.108  ES = -0.09 (HeF<=nHeF) | cross sex- shift in M (and F) |
| Pre/Orbitofrontal (PFC/OFC)* | 69127 ± 6650 | 63027 ± 6275 | 68401 ± 6667 | 63691 ± 6290 | **p < 0.001**  **ES = 0.94**  **(HeM>HeF)** | **p = 0.011**  **ES = 0.11 (HeM>nHeM)** | **p = 0.036**  **ES = -0.11 (HeF<nHeF)** | cross sex- shift in M and F |
| Precuneus* | 21675 ± 2744 | 19796 ± 2554 | 21465 ± 2658 | 20086 ± 2781 | **p < 0.001**  **ES = 0.71**  **(HeM>HeF)** | p = 0.086  ES = 0.08 (HeM=>nHeM) | **p = 0.031**  **ES = -0.11 (HeF<nHeF)** | cross sex- shift in F (and M) |
| Inferior temporal* | 19352 ± 2287 | 17145 ± 2050 | 19085 ± 2344 | 17281 ± 2128 | **p < 0.001**  **ES = 1.02 (HeM>HeF)** | **p = 0.009**  **ES = 0.12**  **(HeM>nHeM)** | p = 0.228  ES = -0.07 (HeF=nHeF) | cross sex- shift in M |
| Thalamus* | 16033 ± 1424 | 14763 ± 1259 | 15897 ± 1438 | 14844 ± 1273 | **p < 0.001**  **ES = 0.94 (HeM>HeF)** | **p = 0.023**  **ES = 0.10**  **(HeM>nHeM)** | p = 0.221  ES = -0.06 (HeF=nHeF) | cross sex- shift in M |
| Fusiform | 25027 ± 2541 | 22578 ± 2294 | 24872 ± 2552 | 22812 ± 2344 | **p < 0.001**  **ES = 1.01 (HeM>HeF)** | p = 0.153  ES = 0.06  (HeM=nHeM) | **p = 0.050**  **ES = -0.10**  **(HeF<nHeF)** | cross sex- shift in F |
| Lingual | 14418 ± 1620 | 12698 ± 1401 | 14385 ± 1584 | 12880 ± 1513 | **p < 0.001**  **ES = 1.14 (HeM>HeF)** | p = 0.650  ES = 0.02  (HeM=nHeM) | **p = 0.021**  **ES = -0.12**  **(HeF<nHeF)** | cross sex- shift in F |
| Anterior cingulate (ACC) | 11299 ± 2282 | 10203 ± 1783 | 11317 ± 2207 | 10514 ± 1883 | **p < 0.001**  **ES = 0.54 (HeM>HeF)** | p = 0.857  ES = -0.01  (HeM=nHeM) | **p = 0.004**  **ES = -0.17 (HeF<nHeF)** | cross sex- shift in F |
| Cuneus | 4504 ± 810 | 4104 ± 745 | 4453 ± 768 | 4131 ± 782 | **p < 0.001**  **ES = 0.51**  **(HeM>HeF)** | p = 0.157  ES = 0.06  (HeM=nHeM) | p = 0.503  ES = -0.04  (HeF=nHeF) | - |
| Parahippocampus | 9529 ± 994 | 8588 ± 890 | 9538 ± 953 | 8633 ± 852 | **p < 0.001**  **ES = 1.00**  **(HeM>HeF)** | p = 0.841  ES = -0.01  (HeM=nHeM) | p = 0.351  ES = -0.05  (HeF=nHeF) | - |
| Hippocampus | 7947 ± 925 | 7485 ± 769 | 7961 ± 909 | 7516 ± 703 | **p < 0.001**  **ES = 0.54 (HeM>HeF)** | p = 0.719  ES = -0.02  (HeM=nHeM) | p = 0.478  ES = -0.04  (HeF=nHeF) | - |

Means and standard deviations (SD) of regional cortical volumes for each group and ROI are listed. Post-hoc group comparisons were performed to interpret interactions and to quantify sex differences (HeM vs. HeF) and SSB-related differences in males (HeM vs. nHeM) and females (HeF vs. nHeF). Cohen’s *d* effect sizes (ES) are presented together with the observed directionality pattern (right columns). ES was colour-coded and mapped onto a cortical surface for visualization (Figure 6, main text). Although these comparisons were performed to determine ES, we provide p-values of the corresponding group comparisons for completeness. * indicates regions showing significant sex-by-SSB interactions. Results for each individual brain region (exploratory whole brain analysis) are provided in Data S2.

*Results (FA-values)*

MANCOVA revealed no significant effect of sex-by-SSB interaction on the combined dependent variable (F(27, 18614)=1.45, p=0.063, Wilks' Λ = 0.998). There was a significant main effect of sex (F(27, 18614)=27.65, p<0.001, Wilks' Λ = 0.961), SSB (F(27, 18614)=1.79, p=0.007, Wilks' Λ = 0.997) and age (F(27, 18614)=160.95, p<0.001, Wilks' Λ = 0.811). Follow-up tests on the main effect of SSB via ANCOVAs for each tract are given in Table S4. A significant main effect of SSB on FA was observed in two white matter tracts: left and right cingulate bundle (cingulate gyrus; see Figure S5 and Table S4). Post-hoc analyses indicated that SSB-related differences were driven by females with a HeF > nHeF pattern (Table S5). These differences did not follow a cross-sex shift, as both male groups (HeM and nHeM) had larger FA values. The HeM < nHeM difference in FA of the superior longitudinal fasciculus did not survive correction for multiple comparisons. Corresponding descriptives are listed in Table S5.

**Table S4: Main effects of SSB follow-up ANCOVAs for FA.**

| Tract ID | Tract name | *Follow-up* ANCOVA  combined cohort (p) |
| --- | --- | --- |
| FA@25488 | left acoustic radiation | 0.285 |
| FA@25489 | right acoustic radiation | 0.143 |
| FA@25490 | left anterior thalamic radiation | 0.869 |
| FA@25491 | right anterior thalamic radiation | 0.402 |
| **FA@25492** | **left cingulate gyrus part of cingulum (bundle)** | **0.004*** |
| **FA@25493** | **right cingulate gyrus part of cingulum (bundle)** | **0.005*** |
| FA@25494 | left parahippocampal part of cingulum | 0.388 |
| FA@25495 | right parahippocampal part of cingulum | 0.247 |
| FA@25496 | left corticospinal tract | 0.455 |
| FA@25497 | right corticospinal tract | 0.449 |
| FA@25498 | forceps major | 0.195 |
| FA@25499 | forceps minor | 0.741 |
| FA@25500 | left inferior fronto-occipital fasciculus | 0.858 |
| FA@25501 | right inferior fronto-occipital fasciculus | 0.967 |
| FA@25502 | left inferior longitudinal fasciculus | 0.297 |
| FA@25503 | right inferior longitudinal fasciculus | 0.995 |
| FA@25504 | middle cerebellar peduncle | 0.483 |
| FA@25505 | left medial lemniscus | 0.642 |
| FA@25506 | right medial lemniscus | 0.468 |
| FA@25507 | left posterior thalamic radiation | 0.257 |
| FA@25508 | right posterior thalamic radiation | 0.793 |
| FA@25509 | left superior longitudinal fasciculus | 0.535 |
| FA@25510 | right superior longitudinal fasciculus | 0.175 |
| FA@25511 | left superior thalamic radiation | 0.499 |
| FA@25512 | right superior thalamic radiation | 0.172 |
| FA@25513 | left uncinate fasciculus | 0.161 |
| FA@25514 | right uncinate fasciculus | 0.423 |

Statistical results (p-values) of follow-up ANCOVAS for the significant main effect of SSB on FA values. Inter-correlation of FA among all 27 tracts in the total sample was r=0.42, leading to an adjusted alpha level = 0.007, correcting for 27 follow-up ANCOVA tests. After adjustment, the main effect of SSB on FA values in left and right cingulate bundle were significant. These tracts are displayed in Figure S5.

**Figure S5: White matter tracts for which a main effect of SSB on FA was found.
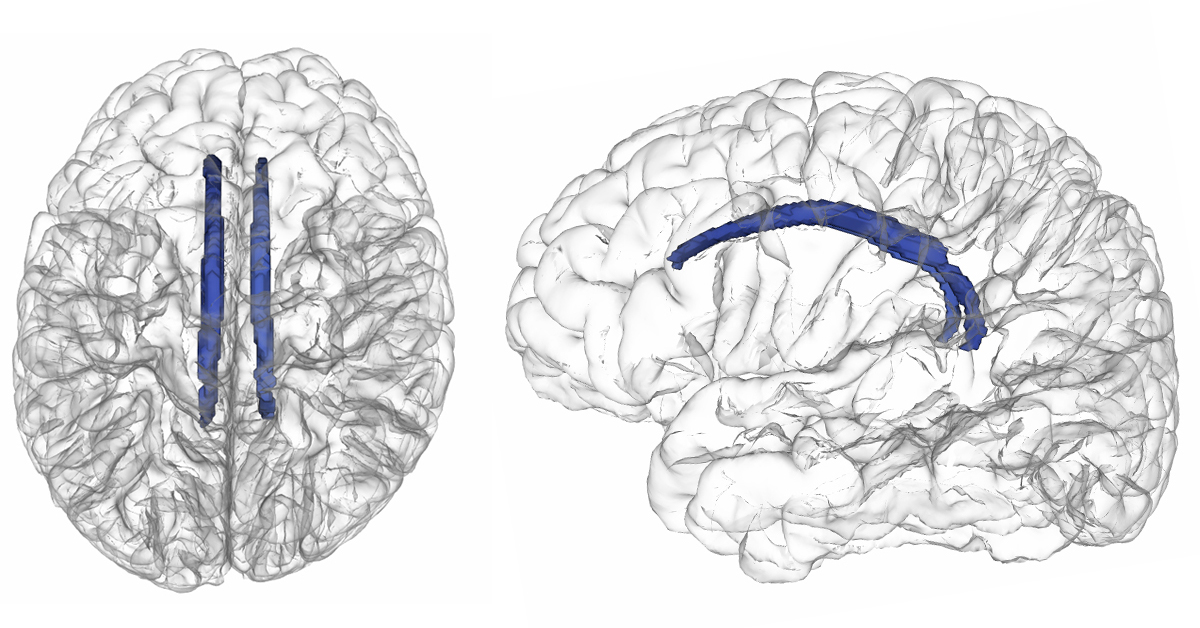
**

**Table S5: FA descriptives.**

| Tract ID | Group | Mean FA | SD | pattern |
| --- | --- | --- | --- | --- |
| FA@25492 | HeM | 0.538 | 0.035 | HeM = nHeM   p = 0.554 |
|  | nHeM | 0.540 | 0.043 |  |
|  | **HeF** | **0.529** | **0.034** | **HeF>nHeF**  **p = 0.001** |
|  | **nHeF** | **0.525** | **0.033** |  |
| FA@25493 | HeM | 0.501 | 0.035 | HeM = nHeM   p = 0.435 |
|  | nHeM | **0.502** | **0.041** |  |
|  | **HeF** | **0.492** | **0.034** | **HeF>nHeF**  **p = 0.002** |
|  | **nHeF** | **0.489** | **0.033** |  |

Means and SD for each group of white matter tracts with a significant SSB main effect. Data S2 contains descriptives for all other FA measures.

**Genome wide association study (GWAS) and generation of polygenic scores for SSB**

*GWAS*

Among 502,543 individuals in UK Biobank, we excluded those without genotype information (N_exclude_=14,248). We then performed a K-means clustering analysis on the first 4 principal components of the genetic data to identify four clusters ([Ganna & Verweij, 2019](#_ENREF_8)). The first cluster was used to identify “White-European” in the analysis, while the other three clusters were excluded for further analyses (N_exclude_=31,406). We then excluded individuals with gender identity disorder, non-consistent/non-valid answers to SSB, and those who were included in the MRI study, yielding a final sample of 393,973 individuals for GWAS. SNPs with low imputation quality (INFO score less than 0.8) or a MAF below 0.01 were excluded in both GWAS and further polygenic scores generation. We ran a GWAS for SSB, adjusting for sex, year of birth, 10 genetic principal components, and batch number using glm in PLINK 2.0 (https://www.cog-genomics.org/plink/2.0/). The Manhattan plot was created in R 4.0.0 (Figure S6).

*Generation of polygenic scores for SSB (PS-SSB)*

The polygenic scores of SSB for individuals included in the MRI study were generated based on the above GWAS summary statistics using the classic polygenic score method ([Choi & Mak, 2020](#_ENREF_6)) in PLINK2.0 and R 4.0.0. We created ten polygenic scores for each individual with P-value threshold of 5e-08, 1e-06, 1e-04, 0.001, 0.01, 0.05, 0.1, 0.2, 0.5, and 1. The percent of variation of the binary phenotypes (SSB) explained by each polygenic score was estimated using Nagelkerke’s R^2^. The best p-value threshold (0.1) was then chosen as PS-SSB for further analysis based on the Nagelkerke’s R^2^ (Table S6).

**Table S6. Comparison of Nagelkerke’s R^2^ for best P-value threshold.**

| P-value threshold | 1 | 0.5 | 0.2 | **0.1** | 0.05 | 0.01 | 0.001 | 1e-04 | 1e-06 | 5e-08 |
| --- | --- | --- | --- | --- | --- | --- | --- | --- | --- | --- |
| Nagelkerke’s R^2^ (%) | 0.188 | 0.200 | 0.190 | **0.217** | 0.130 | 0.096 | 0.052 | 0.140 | 0.054 | 0.012 |

**Figure S6. Manhattan plot for SSB GWAS.**


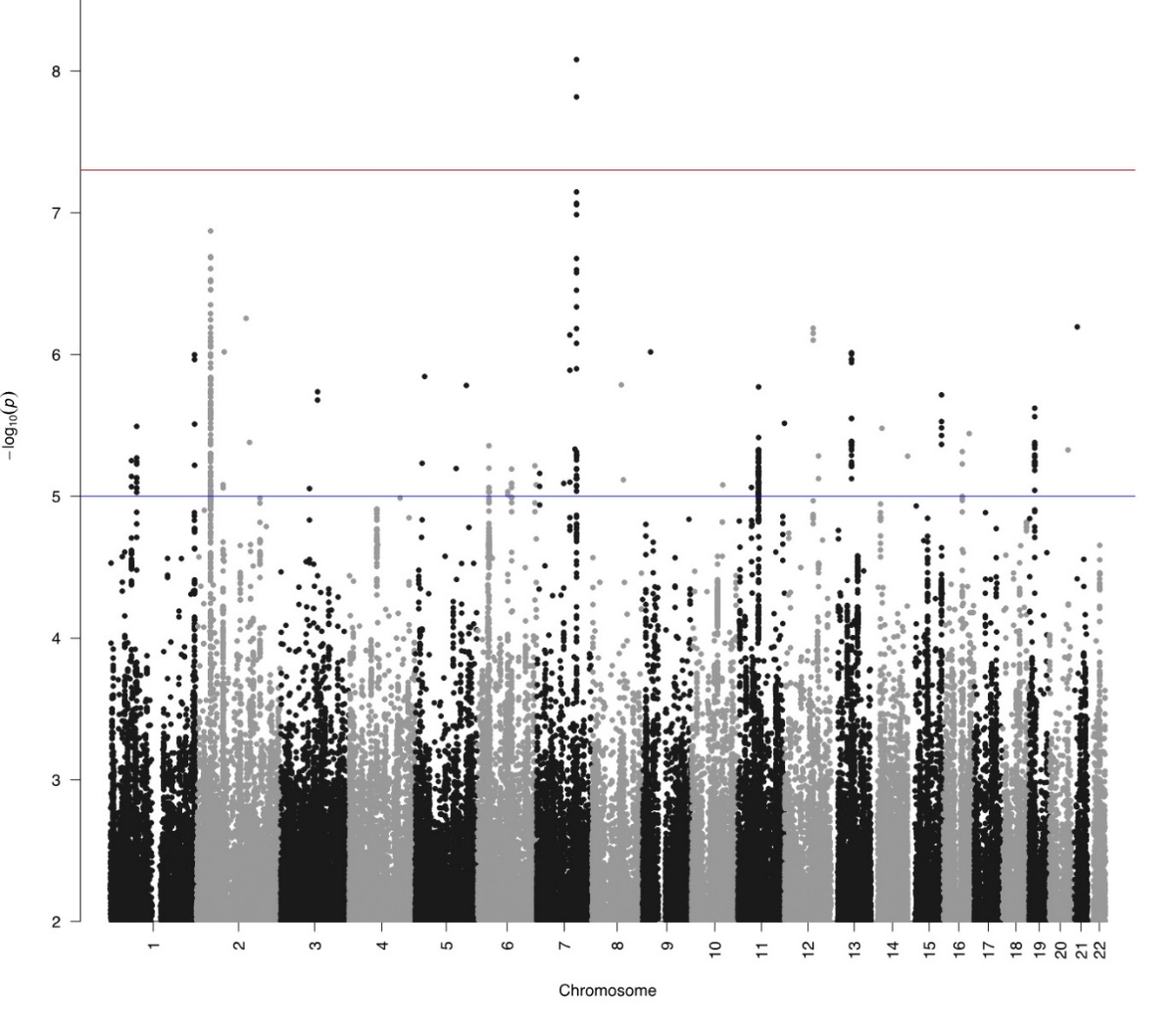


The only SNP reaching genome-wide significance was located on chromosome 7 (rs10261857, OR=1.08, p value=8.3*10^-9^). Note, we excluded individuals with imaging data and our SSB measure was constructed using information from three time points, whereas Ganna et al. considered only baseline measures. However, the here identified SNP is in exact agreement with that found by Ganna et al. (2019).

**Genetic correlations between published SSB and structural brain phenotype GWAS**

We used LDSCORE regression software (v1.0.1; <https://github.com/bulik/ldsc>) to compute genetic correlations between GWAS summary statistics. We reformatted the summary statistics using the munge_sumstats.py Python script, including filtering to HapMap3 SNPs to minimize bias from poor imputation quality. SNPs included in the analyses were filtered to include only those with MAF > 1%. For SSB data in UK Biobank, we also applied a filter to include only imputation INFO > 0.9 (INFO was not available in the brain phenotype summary statistics). We specified the number of samples included for SSB (N_SSB_ = 12,369 and N_No-SBB_ = 337,090) and brain phenotypes (number of subjects differed slightly across IDP grouping: N_T1-FAST_ROIs_ = 8,427, N_T1-FIRST_ = 8,428 and N_FreeSurfer_ = 8,411). We analysed those brain regions making up the ROIs included in the hypothesis-driven ROI analysis (see above). Filtering on mean chi-squared > 1.02 resulted in the inclusion of 40 regions for genetic correlation analyses. The reformatted summary statistics were used for genetic correlation analyses (using the ldsc.py Python script). We used linkage disequilibrium measurements taken from the 1000 Genomes Project as provided by Bulik-Sullivan et al. ([Bulik-Sullivan et al., 2015](#_ENREF_5)), including only European populations. Results are shown in Table S7.

**Table S7. Genetic correlations (r_g_) between SSB and brain phenotype GWAS**

|  |  | **Genetic correlation with** | | |
| --- | --- | --- | --- | --- |
|  |  | **SSB**** | | |
| **Brain phenotype*** | **N** | **r_g_** | **SE** | ***p* - value** |
| **IDP_T1_FAST_ROIs_L_cuneal_cortex** | **8427** | **0.302** | **0.15** | **5.00E-02** |
| IDP_T1_FAST_ROIs_L_temp_fusif_cortex_ant | 8427 | 0.251 | 0.16 | 1.20E-01 |
| IDP_T1_FAST_ROIs_L_precun_cortex | 8427 | 0.17 | 0.12 | 1.70E-01 |
| IDP_T1_FAST_ROIs_R_cuneal_cortex | 8427 | 0.118 | 0.11 | 2.70E-01 |
| IDP_T1_FAST_ROIs_L_parahipp_gyrus_ant | 8427 | 0.128 | 0.13 | 3.10E-01 |
| IDP_T1_FAST_ROIs_L_cing_gyrus_ant | 8427 | -0.135 | 0.15 | 3.60E-01 |
| IDP_T1_FIRST_right_hippocampus_volume | 8428 | -0.137 | 0.15 | 3.60E-01 |
| IDP_T1_FAST_ROIs_L_intracalc_cortex | 8427 | 0.115 | 0.13 | 3.70E-01 |
| IDP_T1_FIRST_left_hippocampus_volume | 8428 | -0.122 | 0.14 | 3.70E-01 |
| IDP_T1_FIRST_left_thalamus_volume | 8428 | -0.11 | 0.12 | 3.80E-01 |
| IDP_T1_FAST_ROIs_R_front_orb_cortex | 8427 | 0.116 | 0.14 | 4.00E-01 |
| IDP_T1_FAST_ROIs_R_lingual_gyrus | 8427 | -0.095 | 0.14 | 5.10E-01 |
| IDP_T1_FAST_ROIs_L_lingual_gyrus | 8427 | 0.084 | 0.13 | 5.30E-01 |
| IDP_T1_FAST_ROIs_L_inf_temp_gyrus_post | 8427 | 0.088 | 0.15 | 5.40E-01 |
| volume_Left-Thalamus-Proper | 8411 | 0.094 | 0.16 | 5.50E-01 |
| IDP_T1_FAST_ROIs_R_inf_temp_gyrus_tempocc | 8427 | 0.109 | 0.2 | 5.80E-01 |
| IDP_T1_FAST_ROIs_R_intracalc_cortex | 8427 | 0.063 | 0.11 | 5.80E-01 |
| IDP_T1_FAST_ROIs_L_inf_temp_gyrus_tempocc | 8427 | 0.067 | 0.13 | 6.10E-01 |
| IDP_T1_FAST_ROIs_R_frontal_pole | 8427 | -0.09 | 0.18 | 6.10E-01 |
| IDP_T1_FAST_ROIs_R_cing_gyrus_ant | 8427 | -0.07 | 0.16 | 6.50E-01 |
| IDP_T1_FAST_ROIs_L_front_orb_cortex | 8427 | -0.061 | 0.14 | 6.60E-01 |
| IDP_T1_FAST_ROIs_L_parahipp_gyrus_post | 8427 | -0.053 | 0.13 | 6.70E-01 |
| IDP_T1_FAST_ROIs_R_parahipp_gyrus_ant | 8427 | 0.042 | 0.12 | 7.40E-01 |
| IDP_T1_FIRST_right_thalamus_volume | 8428 | -0.041 | 0.13 | 7.60E-01 |
| IDP_T1_FAST_ROIs_R_supracalc_cortex | 8427 | 0.035 | 0.12 | 7.70E-01 |
| IDP_T1_FAST_ROIs_R_temp_occ_fusif_cortex | 8427 | -0.039 | 0.15 | 7.90E-01 |
| IDP_T1_FAST_ROIs_L_inf_temp_gyrus_ant | 8427 | 0.038 | 0.15 | 8.00E-01 |
| volume_Right-Hippocampus | 8411 | -0.028 | 0.11 | 8.00E-01 |
| IDP_T1_FAST_ROIs_L_temp_fusif_cortex_post | 8427 | -0.021 | 0.12 | 8.60E-01 |
| IDP_T1_FAST_ROIs_R_occ_fusif_gyrus | 8427 | 0.034 | 0.19 | 8.60E-01 |
| IDP_T1_FAST_ROIs_R_temp_fusif_cortex_ant | 8427 | 0.042 | 0.25 | 8.70E-01 |
| IDP_T1_FAST_ROIs_L_occ_fusif_gyrus | 8427 | 0.023 | 0.16 | 8.80E-01 |
| volume_Left-Hippocampus | 8411 | -0.014 | 0.11 | 8.90E-01 |
| IDP_T1_FAST_ROIs_R_precun_cortex | 8427 | 0.015 | 0.12 | 9.00E-01 |
| volume_Right-Thalamus-Proper | 8411 | 0.02 | 0.15 | 9.00E-01 |
| IDP_T1_FAST_ROIs_R_inf_temp_gyrus_ant | 8427 | 0.02 | 0.18 | 9.10E-01 |
| IDP_T1_FAST_ROIs_R_temp_fusif_cortex_post | 8427 | 0.015 | 0.12 | 9.10E-01 |
| IDP_T1_FAST_ROIs_R_inf_temp_gyrus_post | 8427 | 0.012 | 0.14 | 9.30E-01 |
| IDP_T1_FAST_ROIs_R_parahipp_gyrus_post | 8427 | 0.009 | 0.13 | 9.50E-01 |
| IDP_T1_FAST_ROIs_L_frontal_pole | 8427 | -0.006 | 0.19 | 9.70E-01 |

Genetic correlation (r_g_), number of participants included in corresponding brain phenotype GWAS (N), standard error (SE), and significance (p-value) are listed. The first entry “*IDP_T1_FAST_ROIs_L_cuneal_cortex*” represents volume of left cuneus cortex according to the Harvard-Oxford cortical atlas. The genetic correlation (r_g_=0.30, *p*=0.050) between SSB and volume of cuneus cortex did not survive a correction for multiple testing (p*_threshold_* = 0.001). * Detailed description of the brain phenotype GWASs and cortical labelling can be found in Elliot et al. (2018). ** Description of the SSB GWAS can be found in Ganna et al. (2019).

**Exploratory investigation to test for the role of potential sub-groups in nHeM.**

The bimodal LV1 distribution in nHeM (Figure 4 in main text) indicated the possibility of sub-groups in nHeM. One possible sub-group could contain individuals who have ‘experimented’ with SBB, hence report it, but not necessarily self-identify as nHe. We assumed such individuals to report only few same-sex partners. Since UK Biobank also provided the number of SBB partners, we tested if removing those individuals who report only one or two SSB-partners would change the shape of the LV1 distribution, which we in fact observed (Figure S7). Note, to what extent this approach captured the group we intended is unknown.

**Figure S7.**

**
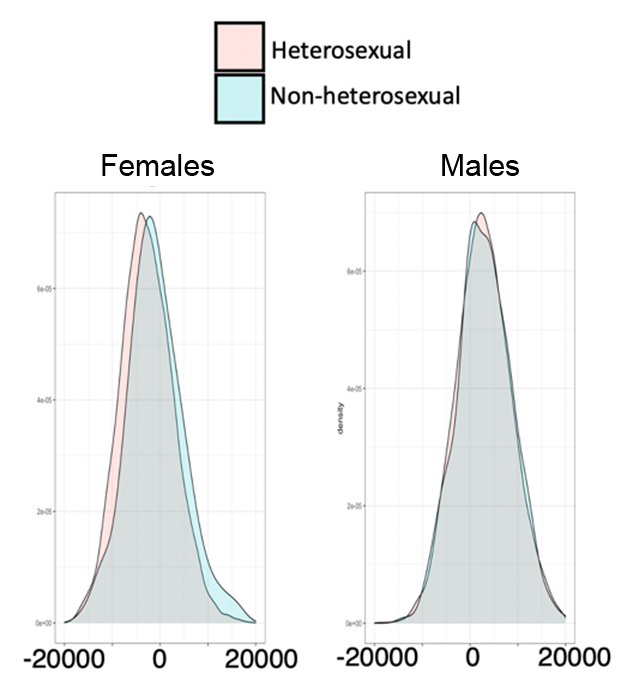
**

LV1 distribution when excluding individuals with 1-2 same-sex partners. The distribution in nHeF did not change, whereas the peak in nHeM was shifted towards smaller values (towards females), indicating the presence of nHeM sub-groups that may show a cross-sex shift in LV1, which could be one potential explanation for the bimodal LV1 distribution in nHeM.

**Adjusting for intracranial volume**

Opinions differ about how and whether markers of brain volume should be corrected for in structural neuroimaging studies. This debate results in part from inconsistent findings, the allometric relationship between brain volume and cerebral substructures, and the difficulty in interpretation of normalized measures ([Hyatt et al., 2020](#_ENREF_9); [Jancke, Liem, & Merillat, 2019](#_ENREF_10); [Luders et al., 2006](#_ENREF_11); [Mankiw et al., 2017](#_ENREF_12); [Mota et al., 2019](#_ENREF_16)). Methods range from not controlling for total brain volume (TBV) or intracranial volume (ICV), controlling for either ICV or TBV, to scaling to ICV and/or TBV and normalizing brain measures to standard templates. Compared to absolute unscaled measures derived from native space (e.g., a participant’s cortical volume of a specific brain area), scaled or normalized measures can be interpreted as relative measures (e.g., percent volume of one’s total volume). However, the decision to apply such corrections and their resulting interpretation depends on the scientific question under examination. For example, if groups of interest differ in ICV/TBV by chance, and ICV/TBV may thus confound results of group comparisons, it can be reasonable to control for ICV/TBV. However, this should only be done if ICV/TBV is not related to the trait of interest and not if ICV/TBV differences are in fact a phenotypic feature of the groups or trait under investigation ([Hyatt et al., 2020](#_ENREF_9)). Otherwise, brain differences that were detected and reported based on corrected data would not necessarily reflect residual “true” differences between groups. This is because true differences may have been occluded or artificial differences arise as a result of such correction ([Luders et al., 2006](#_ENREF_11)). In the present analysis, we strongly argue against using brain phenotypes that are ICV/TBV adjusted because sexes differ in head size and brain volume with females commonly showing smaller ICV/TBV compared with males. This difference may originate from biological factors influencing somatic and neural sexual differentiation (e.g., prenatal androgen or genetic factors also hypothesized to play a role in sexual orientation-related neurobiological and behavioral differences). A potential cross-sex shift in brain structure is hypothesized to entail smaller ICV/TBV in nHeM and larger volumes in nHEF. In essence, by adjusting for ICV/TBV one is effectively correcting for the phenotypic feature under study ([Hyatt et al., 2020](#_ENREF_9)). However, to demonstrate this effect and for the purposes of clarity and comparison, we have also performed analyses when adjusting for ICV, which should - because of aforementioned reasons - be treated with caution.

**Supplementary references**

Abé, C., Johansson, E., Allzen, E., & Savic, I. (2014). Sexual orientation related differences in cortical thickness in male individuals. *PLoS One, 9*(12), e114721. doi:10.1371/journal.pone.0114721

Abé, C., Rahman, Q., Langstrom, N., Ryden, E., Ingvar, M., & Landen, M. (2018). Cortical brain structure and sexual orientation in adult females with bipolar disorder or attention deficit hyperactivity disorder. *Brain Behav, 8*(7), e00998. doi:10.1002/brb3.998

Alfaro-Almagro, F., Jenkinson, M., Bangerter, N. K., Andersson, J. L. R., Griffanti, L., Douaud, G., . . . Smith, S. M. (2018). Image processing and Quality Control for the first 10,000 brain imaging datasets from UK Biobank. *Neuroimage, 166*, 400-424. doi:10.1016/j.neuroimage.2017.10.034

Branstrom, R. (2017). Minority stress factors as mediators of sexual orientation disparities in mental health treatment: a longitudinal population-based study. *J Epidemiol Community Health, 71*(5), 446-452. doi:10.1136/jech-2016-207943

Bulik-Sullivan, B., Finucane, H. K., Anttila, V., Gusev, A., Day, F. R., Loh, P. R., . . . Neale, B. M. (2015). An atlas of genetic correlations across human diseases and traits. *Nat Genet, 47*(11), 1236-1241. doi:10.1038/ng.3406

Choi, S. W., & Mak, T. S. (2020). Tutorial: a guide to performing polygenic risk score analyses. *15*(9), 2759-2772. doi:10.1038/s41596-020-0353-1

Frisell, T., Lichtenstein, P., Rahman, Q., & Langstrom, N. (2010). Psychiatric morbidity associated with same-sex sexual behaviour: influence of minority stress and familial factors. *Psychol Med, 40*(2), 315-324. doi:10.1017/s0033291709005996

Ganna, A., & Verweij, K. J. H. (2019). Large-scale GWAS reveals insights into the genetic architecture of same-sex sexual behavior. *Science, 365*(6456), eaat7693. doi:10.1126/science.aat7693

Hyatt, C. S., Owens, M. M., Crowe, M. L., Carter, N. T., Lynam, D. R., & Miller, J. D. (2020). The quandary of covarying: A brief review and empirical examination of covariate use in structural neuroimaging studies on psychological variables. *Neuroimage, 205*, 116225. doi:10.1016/j.neuroimage.2019.116225

Jancke, L., Liem, F., & Merillat, S. (2019). Scaling of brain compartments to brain size. *Neuroreport, 30*(8), 573-579. doi:10.1097/wnr.0000000000001249

Luders, E., Narr, K. L., Thompson, P. M., Rex, D. E., Woods, R. P., Deluca, H., . . . Toga, A. W. (2006). Gender effects on cortical thickness and the influence of scaling. *Hum Brain Mapp, 27*(4), 314-324. doi:10.1002/hbm.20187

Mankiw, C., Park, M. T. M., Reardon, P. K., Fish, A. M., Clasen, L. S., Greenstein, D., & Giedd, J. N. (2017). Allometric Analysis Detects Brain Size-Independent Effects of Sex and Sex Chromosome Complement on Human Cerebellar Organization. *37*(21), 5221-5231. doi:10.1523/jneurosci.2158-16.2017

Manzouri, A., & Savic, I. (2018a). Cerebral sex dimorphism and sexual orientation. *Hum Brain Mapp, 39*(3), 1175-1186. doi:10.1002/hbm.23908

Manzouri, A., & Savic, I. (2018b). Multimodal MRI suggests that male homosexuality may be linked to cerebral midline structures. *PLoS One, 13*(10), e0203189. doi:10.1371/journal.pone.0203189

Miller, K. L., Alfaro-Almagro, F., Bangerter, N. K., Thomas, D. L., Yacoub, E., & Xu, J. (2016). Multimodal population brain imaging in the UK Biobank prospective epidemiological study. *Nat Neurosci, 19*(11), 1523-1536. doi:10.1038/nn.4393

Mota, B., Dos Santos, S. E., Ventura-Antunes, L., Jardim-Messeder, D., Neves, K., Kazu, R. S., . . . Herculano-Houzel, S. (2019). White matter volume and white/gray matter ratio in mammalian species as a consequence of the universal scaling of cortical folding. *Proc Natl Acad Sci U S A, 116*(30), 15253-15261. doi:10.1073/pnas.1716956116

Plöderl, M., & Tremblay, P. (2015). Mental health of sexual minorities. A systematic review. *International review of psychiatry, 27*(5), 367-385.

Ponseti, J., Siebner, H. R., Kloppel, S., Wolff, S., Granert, O., Jansen, O., . . . Bosinski, H. A. (2007). Homosexual women have less grey matter in perirhinal cortex than heterosexual women. *PLoS One, 2*(8), e762. doi:10.1371/journal.pone.0000762

Sandfort, T., Graaf, R., Have, M., Ransome, Y., & Schnabel, P. (2014). Same-Sex Sexuality and Psychiatric Disorders in the Second Netherlands Mental Health Survey and Incidence Study (NEMESIS-2). *LGBT Health, 1*. doi:10.1089/lgbt.2014.0031

Sandfort, T. G., de Graaf, R., Bijl, R. V., & Schnabel, P. (2001). Same-sex sexual behavior and psychiatric disorders: findings from the Netherlands Mental Health Survey and Incidence Study (NEMESIS). *Arch Gen Psychiatry, 58*(1), 85-91.

Sankoh, A. J., Huque, M. F., & Dubey, S. D. (1997). Some comments on frequently used multiple endpoint adjustment methods in clinical trials. *Stat Med, 16*(22), 2529-2542.
