# Supplementary material for: Cross‐sex shifts in two brain imaging phenotypes and their relation to polygenic scores for same‐sex sexual behavior: A study of 18,645 individuals from the UK Biobank
Source: Hum Brain Mapp. 2021 Feb 26;42(7):2292–304. doi: 10.1002/hbm.25370 (PMC8046142; doi:10.1002/hbm.25370)
Supplement: Supplementary file 2 — Appendix S2: Supporting information [file HBM-42-2292-s001.docx]

Supplementary Data S1

PS-SSB regression

|  | **p-values** |
| --- | --- |
|  | sign. at p<0.05 |
|  | sign. after adjustment |
|  | post hoc/exploratory |

| **(Volumetric)**  **Region@UKBB_ID** | **Main effect of PS-SSB in combined group** | **Main effect of PS-SSB in males (exploratory)** | **Main effect of PS-SSB in females (exploratory)** | **Sex * PS-SSB interaction (combined group)** | **SSB*sex*PS-SSB interaction** | **sex*PS-SSB interaction in He (post-hoc 3-way)** | **sex*PS-SSB interaction in nHe (post-hoc 3-way)** | **in HeM (post hoc)** | **in HeF (post hoc)** | **in nHeM (post hoc)** | **in nHeF (post hoc)** |
| --- | --- | --- | --- | --- | --- | --- | --- | --- | --- | --- | --- |
| R_mid_temp_toc@25807 | 0.37 | 0.656 | 0.406 | 0.834 | 0.0003 | 0.342 | 0.001 | 0.93 | 0.179 | 0.03 | 0.018 |
| Right_InferiorTemporal_toc@25813 | 0.0017 | 0.018 | 0.04 | 0.652 | 0.041 | 0.978 | 0.045 | 0.059 | 0.025 | 0.035 | 0.432 |
| R_Putamen@25016 | 0.436 | 0.339 | 0.93 | 0.495 | 0.079 | 0.769 | 0.067 | 0.602 | 0.872 | 0.027 | 0.557 |
| L_Putamen@25015 | 0.485 | 0.122 | 0.5 | 0.104 | 0.079 | 0.223 | 0.043 | 0.297 | 0.515 | 0.009 | 0.687 |
| L_postcentral@25814 | 0.559 | 0.589 | 0.179 | 0.189 | 0.089 | 0.101 | 0.143 | 0.498 | 0.093 | 0.616 | 0.131 |
| R_mid_front_g@25789 | 0.071 | 0.344 | 0.11 | 0.709 | 0.091 | 0.929 | 0.092 | 0.28 | 0.184 | 0.522 | 0.098 |
| Left_TemporalOccipitalFusiform@25858 | 0.637 | 0.662 | 0.819 | 0.855 | 0.11 | 0.887 | 0.096 | 0.988 | 0.813 | 0.027 | 0.849 |
| Right_TemporalOccipitalFusiform@25859 | 0.872 | 0.305 | 0.393 | 0.18 | 0.113 | 0.1 | 0.232 | 0.259 | 0.233 | 0.807 | 0.152 |
| L_sup_temp_ant@25798 | 0.737 | 0.297 | 0.116 | 0.067 | 0.15 | 0.03 | 0.323 | 0.173 | 0.083 | 0.254 | 0.742 |
| R_parietal_operculum@25867 | 0.754 | 0.572 | 0.278 | 0.257 | 0.165 | 0.175 | 0.236 | 0.525 | 0.18 | 0.786 | 0.125 |
| L_suppl_motor_juxta@25832 | 0.708 | 0.484 | 0.879 | 0.536 | 0.194 | 0.726 | 0.183 | 0.621 | 0.976 | 0.352 | 0.335 |
| Right_TemporalFusiform_post@25857 | 0.357 | 0.593 | 0.442 | 0.922 | 0.209 | 0.817 | 0.224 | 0.298 | 0.391 | 0.03 | 0.706 |
| Left_InferiorTemporal_toc@25812 | 0.006 | 0.007 | 0.267 | 0.192 | 0.217 | 0.374 | 0.159 | 0.031 | 0.263 | 0.015 | 0.834 |
| L_temp_pole@25796 | 0.655 | 0.098 | 0.266 | 0.047 | 0.224 | 0.026 | 0.463 | 0.079 | 0.181 | 0.813 | 0.416 |
| R_mid_temp_ant@25803 | 0.5 | 0.187 | 0.703 | 0.214 | 0.238 | 0.345 | 0.171 | 0.377 | 0.674 | 0.033 | 0.931 |
| L_supra_marginal_ant@25818 | 0.131 | 0.162 | 0.447 | 0.611 | 0.246 | 0.449 | 0.294 | 0.13 | 0.587 | 0.863 | 0.219 |
| Right_Parahippocampal_ant@25849 | 0.81 | 0.518 | 0.741 | 0.477 | 0.274 | 0.638 | 0.214 | 0.66 | 0.84 | 0.38 | 0.364 |
| L_insula@25784 | 0.417 | 0.863 | 0.177 | 0.306 | 0.284 | 0.429 | 0.199 | 0.944 | 0.199 | 0.254 | 0.466 |
| L_parietal_operculum@25866 | 0.324 | 0.761 | 0.268 | 0.623 | 0.291 | 0.514 | 0.345 | 0.711 | 0.15 | 0.804 | 0.114 |
| R_sup_temp_ant@25799 | 0.284 | 0.93 | 0.103 | 0.251 | 0.292 | 0.32 | 0.208 | 0.85 | 0.197 | 0.775 | 0.048 |
| L_sup_front_g@25786 | 0.321 | 0.77 | 0.263 | 0.595 | 0.302 | 0.437 | 0.409 | 0.976 | 0.23 | 0.297 | 0.865 |
| L_sup_temp_post@25800 | 0.664 | 0.261 | 0.071 | 0.041 | 0.321 | 0.027 | 0.591 | 0.256 | 0.04 | 0.754 | 0.3 |
| RIght_Intracalcarine@25829 | 0.94 | 0.842 | 0.739 | 0.713 | 0.324 | 0.847 | 0.271 | 0.972 | 0.729 | 0.129 | 0.882 |
| L_mid_temp_ant@25802 | 0.173 | 0.048 | 0.917 | 0.117 | 0.327 | 0.197 | 0.222 | 0.144 | 0.79 | 0.009 | 0.494 |
| Right_TemporalFusiform_ant@25855 | 0.582 | 0.759 | 0.252 | 0.327 | 0.327 | 0.47 | 0.258 | 0.976 | 0.277 | 0.236 | 0.625 |
| L_angular_g@25822 | 0.513 | 0.613 | 0.689 | 0.922 | 0.334 | 0.89 | 0.352 | 0.781 | 0.62 | 0.391 | 0.543 |
| R_front_operculum@25863 | 0.519 | 0.676 | 0.628 | 0.992 | 0.348 | 0.882 | 0.324 | 0.64 | 0.763 | 0.845 | 0.279 |
| Left_Cuneus@25844 | 0.552 | 0.885 | 0.493 | 0.726 | 0.376 | 0.647 | 0.41 | 0.737 | 0.288 | 0.38 | 0.038 |
| L_mid_temp_toc@25806 | 0.013 | 0.064 | 0.104 | 0.786 | 0.4 | 0.908 | 0.421 | 0.073 | 0.071 | 0.591 | 0.498 |
| L_Accumbens@25023 | 0.651 | 0.428 | 0.839 | 0.464 | 0.409 | 0.623 | 0.324 | 0.646 | 0.815 | 0.074 | 0.816 |
| L_pars_operc@25792 | 0.333 | 0.543 | 0.043 | 0.072 | 0.427 | 0.051 | 0.693 | 0.463 | 0.034 | 0.584 | 0.865 |
| L_lat_occip_inf@25826 | 0.0002 | 0.009 | 0.008 | 0.87 | 0.437 | 0.968 | 0.433 | 0.015 | 0.006 | 0.373 | 0.8 |
| R_lat_occip_inf@25827 | 0.794 | 0.787 | 0.932 | 0.882 | 0.438 | 0.771 | 0.45 | 0.611 | 0.888 | 0.237 | 0.928 |
| R_angular_g@25823 | 0.091 | 0.975 | 0.016 | 0.097 | 0.45 | 0.066 | 0.695 | 0.787 | 0.015 | 0.339 | 0.76 |
| L_Caudate@25013 | 0.854 | 0.372 | 0.5 | 0.263 | 0.455 | 0.315 | 0.322 | 0.406 | 0.566 | 0.525 | 0.406 |
| R_sup_parietal@25817 | 0.944 | 0.334 | 0.385 | 0.193 | 0.462 | 0.291 | 0.314 | 0.595 | 0.324 | 0.033 | 0.579 |
| R_Accumbens@25024 | 0.832 | 0.291 | 0.144 | 0.08 | 0.466 | 0.084 | 0.735 | 0.448 | 0.075 | 0.188 | 0.088 |
| Right_Parahippocampal_post@25851 | 0.057 | 0.043 | 0.508 | 0.287 | 0.476 | 0.225 | 0.632 | 0.031 | 0.544 | 0.707 | 0.776 |
| L_mid_front_g@25788 | 0.482 | 0.999 | 0.325 | 0.506 | 0.476 | 0.624 | 0.417 | 0.774 | 0.298 | 0.226 | 0.929 |
| Right_InferiorTemporal_post@25811 | 0.452 | 0.825 | 0.385 | 0.686 | 0.478 | 0.546 | 0.557 | 0.823 | 0.503 | 0.049 | 0.295 |
| Right_FrontalOrbital@25847 | 0.012 | 0.149 | 0.037 | 0.769 | 0.479 | 0.668 | 0.536 | 0.142 | 0.019 | 0.961 | 0.343 |
| Left_InferiorTemporal_ant@25808 | 0.05 | 0.118 | 0.235 | 0.684 | 0.486 | 0.812 | 0.444 | 0.188 | 0.246 | 0.184 | 0.851 |
| R_supra_marginal_ant@25819 | 0.571 | 0.906 | 0.506 | 0.711 | 0.486 | 0.828 | 0.441 | 0.734 | 0.499 | 0.263 | 0.975 |
| Left_TemporalFusiform_ant@25854 | 0.108 | 0.759 | 0.043 | 0.272 | 0.487 | 0.365 | 0.358 | 0.609 | 0.047 | 0.44 | 0.553 |
| Right_Precuneus@25843 | 0.869 | 0.472 | 0.328 | 0.234 | 0.495 | 0.311 | 0.375 | 0.716 | 0.265 | 0.091 | 0.717 |
| R_supra_marginal_post@25821 | 0.273 | 0.994 | 0.131 | 0.305 | 0.504 | 0.268 | 0.646 | 0.783 | 0.054 | 0.18 | 0.061 |
| R_sup_front_g@25787 | 0.564 | 0.938 | 0.367 | 0.508 | 0.559 | 0.434 | 0.704 | 0.863 | 0.327 | 0.662 | 0.894 |
| Left_Supracalcarine@25874 | 0.388 | 0.477 | 0.625 | 0.821 | 0.572 | 0.771 | 0.617 | 0.425 | 0.623 | 0.616 | 0.921 |
| Left_Subcallosal@25834 | 0.409 | 0.565 | 0.066 | 0.102 | 0.573 | 0.139 | 0.371 | 0.74 | 0.058 | 0.183 | 0.986 |
| R_lat_occip_sup@25825 | 0.177 | 0.051 | 0.953 | 0.135 | 0.582 | 0.112 | 0.844 | 0.053 | 0.827 | 0.701 | 0.559 |
| R_temp_pole@25797 | 0.613 | 0.265 | 0.057 | 0.036 | 0.584 | 0.029 | 0.946 | 0.267 | 0.042 | 0.84 | 0.756 |
| L_lat_occip_sup@25824 | 0.965 | 0.663 | 0.624 | 0.513 | 0.588 | 0.602 | 0.475 | 0.895 | 0.529 | 0.099 | 0.644 |
| Left_Paracingulate@25836 | 0.064 | 0.406 | 0.069 | 0.57 | 0.597 | 0.49 | 0.683 | 0.448 | 0.053 | 0.738 | 0.825 |
| Right_Thalamus_vol@25012 | 0.147 | 0.649 | 0.1 | 0.46 | 0.605 | 0.408 | 0.75 | 0.58 | 0.055 | 0.519 | 0.338 |
| R_Caudate@25014 | 0.491 | 0.305 | 0.933 | 0.405 | 0.621 | 0.448 | 0.499 | 0.373 | 0.901 | 0.313 | 0.917 |
| Left_FrontalOrbital@25846 | 0.024 | 0.226 | 0.047 | 0.689 | 0.633 | 0.786 | 0.59 | 0.133 | 0.033 | 0.212 | 0.625 |
| L_Precentral@25794 | 0.597 | 0.954 | 0.489 | 0.674 | 0.637 | 0.775 | 0.565 | 0.724 | 0.409 | 0.162 | 0.6 |
| Left_Precuneus@25842 | 0.796 | 0.362 | 0.582 | 0.292 | 0.657 | 0.33 | 0.537 | 0.446 | 0.555 | 0.366 | 0.992 |
| Right_OccipitalFusiform@25861 | 0.989 | 0.222 | 0.23 | 0.086 | 0.67 | 0.079 | 0.994 | 0.285 | 0.152 | 0.41 | 0.407 |
| R_suppl_motor_juxta@25833 | 0.51 | 0.547 | 0.732 | 0.832 | 0.675 | 0.886 | 0.69 | 0.537 | 0.637 | 0.953 | 0.579 |
| L_front_operculum@25862 | 0.564 | 0.694 | 0.681 | 0.982 | 0.676 | 0.872 | 0.71 | 0.951 | 0.869 | 0.03 | 0.176 |
| R_planum_polare@25869 | 0.333 | 0.876 | 0.131 | 0.255 | 0.681 | 0.219 | 0.888 | 0.81 | 0.12 | 0.736 | 0.926 |
| Right_Supracalcarine@25875 | 0.782 | 0.697 | 0.411 | 0.404 | 0.682 | 0.402 | 0.815 | 0.652 | 0.447 | 0.546 | 0.774 |
| L_supra_marginal_post@25820 | 0.814 | 0.866 | 0.862 | 0.995 | 0.685 | 0.909 | 0.708 | 0.799 | 0.908 | 0.853 | 0.7 |
| L_Pars_Triangularis@25790 | 0.859 | 0.942 | 0.851 | 0.941 | 0.706 | 0.966 | 0.691 | 0.741 | 0.67 | 0.299 | 0.224 |
| Left_FrontalPole@25782 | 0.612 | 0.436 | 0.129 | 0.11 | 0.71 | 0.088 | 0.984 | 0.384 | 0.114 | 0.977 | 0.974 |
| Left_Thalamus_vol@25011 | 0.575 | 0.768 | 0.249 | 0.329 | 0.713 | 0.32 | 0.889 | 0.975 | 0.132 | 0.154 | 0.139 |
| R_insula@25785 | 0.661 | 0.334 | 0.099 | 0.069 | 0.726 | 0.086 | 0.466 | 0.417 | 0.093 | 0.383 | 0.857 |
| Right_Hippocampus_vol@25020 | 0.374 | 0.5 | 0.583 | 0.857 | 0.727 | 0.967 | 0.646 | 0.678 | 0.664 | 0.223 | 0.63 |
| Right_FrontalPole@25783 | 0.405 | 0.911 | 0.287 | 0.531 | 0.74 | 0.474 | 0.88 | 0.9 | 0.225 | 0.791 | 0.616 |
| Left_FrontalMedial@25830 | 0.156 | 0.942 | 0.037 | 0.148 | 0.743 | 0.178 | 0.51 | 0.994 | 0.043 | 0.794 | 0.569 |
| Right_Paracingulate@25837 | 0.394 | 0.676 | 0.422 | 0.832 | 0.751 | 0.865 | 0.735 | 0.606 | 0.388 | 0.58 | 0.933 |
| R_Amygdala@25022 | 0.147 | 0.04 | 0.885 | 0.088 | 0.761 | 0.066 | 0.929 | 0.012 | 0.826 | 0.129 | 0.084 |
| Left_Intracalcarine@25828 | 0.7 | 0.903 | 0.667 | 0.853 | 0.768 | 0.76 | 0.863 | 0.931 | 0.564 | 0.824 | 0.651 |
| R_sup_temp_post@25801 | 0.255 | 0.242 | 0.66 | 0.566 | 0.781 | 0.632 | 0.718 | 0.295 | 0.649 | 0.563 | 0.955 |
| L_planum_polare@25868 | 0.493 | 0.718 | 0.18 | 0.244 | 0.782 | 0.214 | 0.985 | 0.722 | 0.144 | 0.857 | 0.853 |
| Left_Lingual@25852 | 0.714 | 0.738 | 0.356 | 0.395 | 0.782 | 0.46 | 0.644 | 0.983 | 0.255 | 0.186 | 0.524 |
| Left_InferiorTemporal_post@25810 | 0.676 | 0.708 | 0.819 | 0.891 | 0.783 | 0.951 | 0.787 | 0.857 | 0.897 | 0.36 | 0.558 |
| Right_InferiorTemporal_ant@25809 | 0.408 | 0.486 | 0.645 | 0.825 | 0.785 | 0.845 | 0.806 | 0.681 | 0.864 | 0.21 | 0.152 |
| L_occip_pole@25876 | 0.815 | 0.422 | 0.627 | 0.357 | 0.785 | 0.412 | 0.665 | 0.593 | 0.522 | 0.128 | 0.566 |
| R_central_opercular_cortex@25865 | 0.235 | 0.967 | 0.102 | 0.284 | 0.79 | 0.305 | 0.631 | 0.962 | 0.114 | 0.927 | 0.585 |
| L_planum_temporale@25872 | 0.895 | 0.611 | 0.441 | 0.375 | 0.799 | 0.401 | 0.675 | 0.669 | 0.428 | 0.597 | 0.999 |
| R_occip_pole@25877 | 0.526 | 0.956 | 0.319 | 0.489 | 0.81 | 0.469 | 0.925 | 0.997 | 0.265 | 0.708 | 0.623 |
| Right_Cuneus@25845 | 0.48 | 0.611 | 0.635 | 0.939 | 0.815 | 0.91 | 0.851 | 0.551 | 0.602 | 0.665 | 0.805 |
| L_Pallidum@25017 | 0.931 | 0.349 | 0.277 | 0.155 | 0.815 | 0.19 | 0.562 | 0.464 | 0.245 | 0.176 | 0.682 |
| Left_Parahippocampal_ant@25848 | 0.808 | 0.554 | 0.778 | 0.524 | 0.816 | 0.578 | 0.698 | 0.605 | 0.809 | 0.825 | 0.729 |
| R_Pars_Triangularis@25791 | 0.872 | 0.397 | 0.282 | 0.176 | 0.816 | 0.207 | 0.602 | 0.502 | 0.259 | 0.387 | 0.902 |
| Left_Cingulate_ant@25838 | 0.223 | 0.425 | 0.337 | 0.955 | 0.823 | 0.968 | 0.791 | 0.507 | 0.407 | 0.467 | 0.701 |
| L_central_opercular_cortex@25864 | 0.389 | 0.939 | 0.259 | 0.479 | 0.825 | 0.458 | 0.95 | 0.848 | 0.191 | 0.527 | 0.472 |
| Right_Cingulate_ant@25839 | 0.145 | 0.215 | 0.423 | 0.598 | 0.831 | 0.652 | 0.741 | 0.308 | 0.516 | 0.276 | 0.508 |
| R_Pallidum@25018 | 0.906 | 0.445 | 0.327 | 0.222 | 0.831 | 0.223 | 0.944 | 0.511 | 0.264 | 0.363 | 0.475 |
| Left_Parahippocampal_post@25850 | 0.525 | 0.23 | 0.747 | 0.265 | 0.839 | 0.327 | 0.689 | 0.391 | 0.612 | 0.101 | 0.395 |
| Right_Subcallosal@25835 | 0.664 | 0.461 | 0.153 | 0.135 | 0.85 | 0.146 | 0.63 | 0.507 | 0.145 | 0.544 | 0.932 |
| L_sup_parietal@25816 | 0.249 | 0.506 | 0.342 | 0.87 | 0.859 | 0.836 | 0.924 | 0.453 | 0.267 | 0.705 | 0.573 |
| Right_FrontalMedial@25831 | 0.583 | 0.419 | 0.115 | 0.097 | 0.877 | 0.095 | 0.595 | 0.365 | 0.14 | 0.85 | 0.432 |
| L_mid_temp_post@25804 | 0.999 | 0.618 | 0.611 | 0.475 | 0.892 | 0.474 | 0.974 | 0.744 | 0.48 | 0.333 | 0.435 |
| R_mid_temp_post@25805 | 0.729 | 0.535 | 0.898 | 0.585 | 0.894 | 0.586 | 0.991 | 0.682 | 0.721 | 0.222 | 0.261 |
| R_Cingulate_post@25841 | 0.372 | 0.309 | 0.832 | 0.504 | 0.9 | 0.547 | 0.775 | 0.473 | 0.967 | 0.116 | 0.237 |
| L_Heschl_h1h2@25870 | 0.367 | 0.757 | 0.099 | 0.19 | 0.908 | 0.188 | 0.886 | 0.765 | 0.094 | 0.856 | 0.979 |
| L_Amygdala@25021 | 0.594 | 0.545 | 0.903 | 0.689 | 0.908 | 0.676 | 0.951 | 0.485 | 0.825 | 0.586 | 0.648 |
| Left_TemporalFusiform_post@25856 | 0.816 | 0.63 | 0.41 | 0.363 | 0.915 | 0.417 | 0.762 | 0.866 | 0.303 | 0.161 | 0.351 |
| R_planum_temporale@25873 | 0.832 | 0.926 | 0.687 | 0.739 | 0.917 | 0.737 | 0.976 | 0.91 | 0.705 | 0.831 | 0.9 |
| R_Precentral@25795 | 0.391 | 0.566 | 0.073 | 0.102 | 0.922 | 0.106 | 0.779 | 0.677 | 0.051 | 0.374 | 0.68 |
| R_postcentral@25815 | 0.188 | 0.191 | 0.58 | 0.554 | 0.94 | 0.53 | 0.949 | 0.116 | 0.426 | 0.283 | 0.281 |
| Left_OccipitalFusiform@25860 | 0.705 | 0.872 | 0.488 | 0.558 | 0.954 | 0.579 | 0.864 | 0.974 | 0.388 | 0.392 | 0.549 |
| Left_Hippocampus_vol@25019 | 0.833 | 0.687 | 0.918 | 0.702 | 0.956 | 0.645 | 0.982 | 0.498 | 0.896 | 0.222 | 0.234 |
| L_Cingulate_post@25840 | 0.928 | 0.527 | 0.567 | 0.39 | 0.964 | 0.396 | 0.802 | 0.611 | 0.481 | 0.433 | 0.642 |
| R_Heschl_h1h2@25871 | 0.418 | 0.986 | 0.243 | 0.456 | 0.985 | 0.466 | 0.872 | 0.973 | 0.244 | 0.967 | 0.79 |
| Right_Lingual@25853 | 0.262 | 0.118 | 0.974 | 0.227 | 0.99 | 0.242 | 0.796 | 0.169 | 0.85 | 0.272 | 0.605 |
| R_pars_operc@25793 | 0.959 | 0.906 | 0.84 | 0.824 | 0.994 | 0.82 | 0.964 | 0.833 | 0.909 | 0.723 | 0.673 |

| **(FA-values)**  **FA@UKBB_ID** | **Tract name** | **Main effect of PS-SSB in combined group** | **Main effect of PS-SSB in males (exploratory)** | **Main effect of PS-SSB in females (exploratory)** | **Sex * PS-SSB interaction (combined group)** | **SSB*sex*PS-SSB interaction** | **sex*PS-SSB interaction in He (post-hoc 3-way)** | **sex*PS-SSB interaction in nHe (post-hoc 3-way)** | **in HeM (post hoc)** | **in HeF (post hoc)** | **in nHeM (post hoc)** | **in nHeF (post hoc)** |
| --- | --- | --- | --- | --- | --- | --- | --- | --- | --- | --- | --- | --- |
| FA@25496 | left corticospinal tract | 0.303 | 0.391 | 0.53 | 0.831 | 0.005 | 0.353 | 0.019 | 0.089 | 0.629 | 0.018 | 0.303 |
| FA@25497 | right corticospinal tract | 0.316 | 0.489 | 0.446 | 0.994 | 0.017 | 0.564 | 0.044 | 0.189 | 0.567 | 0.069 | 0.223 |
| FA@25501 | right inferior fronto-occipital fasciculus | 0.996 | 0.865 | 0.859 | 0.807 | 0.018 | 0.448 | 0.044 | 0.567 | 0.62 | 0.23 | 0.043 |
| FA@25502 | left inferior longitudinal fasciculus | 0.725 | 0.074 | 0.164 | 0.023 | 0.028 | 0.006 | 0.153 | 0.022 | 0.121 | 0.228 | 0.392 |
| FA@25500 | left inferior fronto-occipital fasciculus | 0.902 | 0.558 | 0.441 | 0.34 | 0.052 | 0.162 | 0.136 | 0.293 | 0.356 | 0.195 | 0.413 |
| FA@25511 | left superior thalamic radiation | 0.084 | 0.079 | 0.494 | 0.374 | 0.183 | 0.559 | 0.124 | 0.149 | 0.421 | 0.143 | 0.543 |
| FA@25506 | right medial lemniscus | 0.771 | 0.116 | 0.253 | 0.053 | 0.285 | 0.089 | 0.112 | 0.123 | 0.42 | 0.952 | 0.044 |
| FA@25498 | forceps major | 0.355 | 0.603 | 0.052 | 0.098 | 0.289 | 0.148 | 0.141 | 0.585 | 0.11 | 0.918 | 0.046 |
| FA@25504 | middle cerebellar peduncle | 0.074 | 0.481 | 0.058 | 0.513 | 0.301 | 0.41 | 0.334 | 0.531 | 0.037 | 0.601 | 0.423 |
| FA@25490 | left anterior thalamic radiation | 0.719 | 0.388 | 0.171 | 0.118 | 0.308 | 0.095 | 0.434 | 0.404 | 0.12 | 0.943 | 0.204 |
| FA@25509 | left superior longitudinal fasciculus | 0.594 | 0.054 | 0.265 | 0.03 | 0.356 | 0.022 | 0.638 | 0.048 | 0.223 | 0.885 | 0.543 |
| FA@25513 | left uncinate fasciculus | 0.933 | 0.09 | 0.076 | 0.014 | 0.392 | 0.01 | 0.741 | 0.067 | 0.068 | 0.624 | 0.994 |
| FA@25514 | right uncinate fasciculus | 0.207 | 0.137 | 0.768 | 0.365 | 0.413 | 0.304 | 0.507 | 0.14 | 0.913 | 0.88 | 0.296 |
| FA@25507 | left posterior thalamic radiation | 0.343 | 0.442 | 0.03 | 0.044 | 0.525 | 0.068 | 0.24 | 0.534 | 0.04 | 0.365 | 0.437 |
| FA@25488 | left acoustic radiation | 0.303 | 0.072 | 0.696 | 0.105 | 0.557 | 0.088 | 0.787 | 0.061 | 0.672 | 0.76 | 0.886 |
| FA@25510 | right superior longitudinal fasciculus | 0.575 | 0.046 | 0.242 | 0.023 | 0.559 | 0.024 | 0.894 | 0.067 | 0.181 | 0.478 | 0.328 |
| FA@25508 | right posterior thalamic radiation | 0.607 | 0.147 | 0.426 | 0.105 | 0.578 | 0.15 | 0.299 | 0.197 | 0.479 | 0.346 | 0.587 |
| FA@25512 | right superior thalamic radiation | 0.052 | 0.058 | 0.393 | 0.379 | 0.61 | 0.481 | 0.444 | 0.112 | 0.433 | 0.159 | 0.554 |
| FA@25505 | left medial lemniscus | 0.444 | 0.045 | 0.391 | 0.04 | 0.627 | 0.028 | 0.996 | 0.023 | 0.441 | 0.442 | 0.459 |
| FA@25491 | right anterior thalamic radiation | 0.831 | 0.513 | 0.725 | 0.468 | 0.652 | 0.466 | 0.735 | 0.618 | 0.599 | 0.525 | 0.24 |
| FA@25503 | right inferior longitudinal fasciculus | 0.429 | 0.108 | 0.611 | 0.123 | 0.708 | 0.125 | 0.973 | 0.148 | 0.499 | 0.331 | 0.33 |
| FA@25493 | right cingulate gyrus part of cingulum (bundle) | 0.143 | 0.813 | 0.065 | 0.289 | 0.776 | 0.294 | 0.903 | 0.791 | 0.062 | 0.872 | 0.715 |
| FA@25495 | right parahippocampal part of cingulum | 0.59 | 0.195 | 0.583 | 0.183 | 0.796 | 0.21 | 0.585 | 0.223 | 0.6 | 0.527 | 0.898 |
| FA@25494 | left parahippocampal part of cingulum | 0.292 | 0.763 | 0.219 | 0.564 | 0.871 | 0.595 | 0.768 | 0.747 | 0.231 | 0.866 | 0.777 |
| FA@25489 | right acoustic radiation | 0.914 | 0.386 | 0.303 | 0.181 | 0.88 | 0.225 | 0.647 | 0.58 | 0.233 | 0.134 | 0.394 |
| FA@25492 | left cingulate gyrus part of cingulum (bundle) | 0.344 | 0.911 | 0.223 | 0.456 | 0.882 | 0.466 | 0.968 | 0.936 | 0.247 | 0.961 | 0.981 |
| FA@25499 | forceps minor | 0.415 | 0.497 | 0.067 | 0.082 | 0.993 | 0.096 | 0.736 | 0.544 | 0.072 | 0.748 | 0.887 |

| **(LVs)** | Main effect of PS-SSB in combined group | Main effect of PS-SSB in males (exploratory) | Main effect of PS-SSB in females (exploratory) | Sex*PS-SSB interaction (combined group) | SSB*sex*PS-SSB interaction | sex*PS-SSB  interaction in He (explorative) | sex*PS-SSB interaction in nHe (explorative) | in HeM (expl.) | in HeF (expl.) | in nHeM (expl.) | in nHeF (expl.) |
| --- | --- | --- | --- | --- | --- | --- | --- | --- | --- | --- | --- |
| LV1 | 0.467 | 0.634 | 0.126 | 0.169 | 0.839 | 0.157 | 0.904 | 0.721 | 0.084 | 0.431 | 0.565 |
| LV2 | 0.561 | 0.557 | 0.818 | 0.776 | 0.737 | 0.73 | 0.776 | 0.496 | 0.805 | 0.674 | 0.966 |
| LV3 | 0.114 | 0.138 | 0.464 | 0.539 | 0.905 | 0.58 | 0.997 | 0.217 | 0.584 | 0.249 | 0.349 |

**Adjustments (Dubey Armitage-Parmar/Sidak)**

| Modality | # of tests | intercorrelation between all measures tested | p-threshold for significance after correction |
| --- | --- | --- | --- |
| **Volumetric** | **110** | **0.321** | **0.0021** |
| **FA** | **27** | **0.420** | **0.007** |
| **LV** | **3** | **0.002** | **0.017** |
