# Supplementary material for: Cross‐sex shifts in two brain imaging phenotypes and their relation to polygenic scores for same‐sex sexual behavior: A study of 18,645 individuals from the UK Biobank
Source: Hum Brain Mapp. 2021 Feb 26;42(7):2292–304. doi: 10.1002/hbm.25370 (PMC8046142; doi:10.1002/hbm.25370)
Supplement: Supplementary file 3 — Appendix S3: Supporting information [file HBM-42-2292-s003.docx]

Supplementary Data S2

- Results from univariate analyses
- Descriptives for all measures and groups investigated

| **(Volumetric, whole brain)**  **Brain_Region@UKBiobank_index** | **Group** | **Mean** | **SE** | **SD** | **Sex-by-SSB interaction (p-value)** | **HeM vs. HeF (p)** | **HeM vs. nHeM (p)** | **HeF vs. nHeF (p)** | **HeM vs. HeF (Cohen's d )** | **HeM vs. nHeM (Cohen's d )** | **HeF vs. nHeF (Cohen's d )** |
| --- | --- | --- | --- | --- | --- | --- | --- | --- | --- | --- | --- |
| Right_Supracalcarine@25875 | HeM | 768.505 | 1.623 | 156.961806 | **< 0.001*** | < 0.001 | 0.001 | 0.054 | 0.54 | 0.15 | -0.10 |
|  | HeF | 683.434 | 1.537 | 156.6458643 |  |  |  |  |  |  |  |
|  | nHeM | 744.407 | 7.342 | 156.6102124 |  |  |  |  |  |  |  |
|  | nHeF | 699.456 | 8.178 | 156.8812408 |  |  |  |  |  |  |  |
| Left_Intracalcarine@25828 | HeM | 2782.279 | 6.197 | 599.3175056 | **< 0.001*** | < 0.001 | < 0.001 | 0.141 | 0.52 | 0.19 | -0.08 |
|  | HeF | 2470.815 | 5.869 | 598.1487167 |  |  |  |  |  |  |  |
|  | nHeM | 2671.016 | 28.035 | 598.0069877 |  |  |  |  |  |  |  |
|  | nHeF | 2517.611 | 31.227 | 599.0377239 |  |  |  |  |  |  |  |
| Left_FrontalPole@25782 | HeM | 24375.885 | 24.903 | 2408.391777 | **0.001*** | < 0.001 | 0.011 | 0.037 | 0.90 | 0.12 | -0.11 |
|  | HeF | 22207.002 | 23.584 | 2403.601863 |  |  |  |  |  |  |  |
|  | nHeM | 24081.21 | 112.658 | 2403.077269 |  |  |  |  |  |  |  |
|  | nHeF | 22472.506 | 125.486 | 2407.238858 |  |  |  |  |  |  |  |
| Left_InferiorTemporal_toc@25812 | HeM | 3212.234 | 6.025 | 582.6832292 | **0.001*** | < 0.001 | 0.014 | 0.024 | 0.61 | 0.12 | -0.12 |
|  | HeF | 2857.484 | 5.706 | 581.5363056 |  |  |  |  |  |  |  |
|  | nHeM | 3143.372 | 27.255 | 581.3690191 |  |  |  |  |  |  |  |
|  | nHeF | 2926.994 | 30.358 | 582.3674135 |  |  |  |  |  |  |  |
| R_lat_occip_inf@25827 | HeM | 8264.096 | 12.866 | 1244.282561 | **0.002*** | < 0.001 | 0.016 | 0.057 | 0.78 | 0.12 | -0.10 |
|  | HeF | 7294.515 | 12.184 | 1241.752252 |  |  |  |  |  |  |  |
|  | nHeM | 8120.591 | 58.202 | 1241.49109 |  |  |  |  |  |  |  |
|  | nHeF | 7420.024 | 64.829 | 1243.635847 |  |  |  |  |  |  |  |
| RIght_Intracalcarine@25829 | HeM | 2883.67 | 5.921 | 572.6252947 | **0.002*** | < 0.001 | < 0.001 | 0.283 | 0.52 | 0.17 | -0.06 |
|  | HeF | 2587.19 | 5.608 | 571.5484756 |  |  |  |  |  |  |  |
|  | nHeM | 2786.607 | 26.787 | 571.3862379 |  |  |  |  |  |  |  |
|  | nHeF | 2619.757 | 29.837 | 572.3729006 |  |  |  |  |  |  |  |
| R_Caudate@25014 | HeM | 3719.909 | 4.315 | 417.3075741 | 0.003 | < 0.001 | 0.021 | 0.061 | 0.70 | 0.11 | -0.10 |
|  | HeF | 3426.56 | 4.086 | 416.4313608 |  |  |  |  |  |  |  |
|  | nHeM | 3673.88 | 19.519 | 416.3544995 |  |  |  |  |  |  |  |
|  | nHeF | 3467.986 | 21.741 | 417.0646926 |  |  |  |  |  |  |  |
| L_sup_temp_ant@25798 | HeM | 1554.421 | 2.943 | 284.6202064 | 0.004 | < 0.001 | 0.127 | 0.013 | 0.56 | 0.07 | -0.13 |
|  | HeF | 1393.907 | 2.787 | 284.041655 |  |  |  |  |  |  |  |
|  | nHeM | 1533.595 | 13.312 | 283.9546646 |  |  |  |  |  |  |  |
|  | nHeF | 1431.3 | 14.828 | 284.4503593 |  |  |  |  |  |  |  |
| Left_Supracalcarine@25874 | HeM | 539.784 | 1.175 | 113.6353186 | 0.004 | < 0.001 | 0.048 | 0.042 | 0.55 | 0.10 | -0.11 |
|  | HeF | 477.338 | 1.113 | 113.4332121 |  |  |  |  |  |  |  |
|  | nHeM | 528.996 | 5.316 | 113.3941554 |  |  |  |  |  |  |  |
|  | nHeF | 489.603 | 5.921 | 113.5844738 |  |  |  |  |  |  |  |
| Left_Paracingulate@25836 | HeM | 6006.759 | 8.1 | 783.3583663 | 0.006 | < 0.001 | 0.217 | 0.011 | 0.63 | 0.06 | -0.14 |
|  | HeF | 5510.623 | 7.671 | 781.8024887 |  |  |  |  |  |  |  |
|  | nHeM | 5960.385 | 36.642 | 781.6005723 |  |  |  |  |  |  |  |
|  | nHeF | 5616.48 | 40.815 | 782.9674545 |  |  |  |  |  |  |  |
| Right_Precuneus@25843 | HeM | 11069.411 | 14.208 | 1374.068601 | 0.006 | < 0.001 | 0.154 | 0.016 | 0.70 | 0.07 | -0.13 |
|  | HeF | 10115.34 | 13.456 | 1371.390208 |  |  |  |  |  |  |  |
|  | nHeM | 10975.581 | 64.275 | 1371.032607 |  |  |  |  |  |  |  |
|  | nHeF | 10290.675 | 71.594 | 1373.411048 |  |  |  |  |  |  |  |
| Right_FrontalPole@25783 | HeM | 27521.246 | 27.899 | 2698.137662 | 0.006 | < 0.001 | 0.047 | 0.059 | 0.91 | 0.10 | -0.10 |
|  | HeF | 25069.296 | 26.421 | 2692.73935 |  |  |  |  |  |  |  |
|  | nHeM | 27263.736 | 126.209 | 2692.129977 |  |  |  |  |  |  |  |
|  | nHeF | 25339.351 | 140.58 | 2696.791982 |  |  |  |  |  |  |  |
| L_central_opercular_cortex@25864 | HeM | 3961.058 | 5.507 | 532.5869782 | 0.007 | < 0.001 | 0.027 | 0.107 | 0.67 | 0.11 | -0.09 |
|  | HeF | 3606.506 | 5.216 | 531.5971556 |  |  |  |  |  |  |  |
|  | nHeM | 3904.615 | 24.914 | 531.4337825 |  |  |  |  |  |  |  |
|  | nHeF | 3651.973 | 27.751 | 532.3564824 |  |  |  |  |  |  |  |
| Left_Cingulate_ant@25838 | HeM | 5362.372 | 10.628 | 1027.843545 | 0.009 | < 0.001 | 0.723 | 0.002 | 0.49 | 0.02 | -0.17 |
|  | HeF | 4854.32 | 10.065 | 1025.790907 |  |  |  |  |  |  |  |
|  | nHeM | 5344.9 | 48.077 | 1025.517459 |  |  |  |  |  |  |  |
|  | nHeF | 5026.999 | 53.552 | 1027.305479 |  |  |  |  |  |  |  |
| Right_FrontalOrbital@25847 | HeM | 6317.683 | 7.015 | 678.4270296 | 0.009 | < 0.001 | 0.041 | 0.1 | 0.80 | 0.10 | -0.09 |
|  | HeF | 5772.815 | 6.643 | 677.0321903 |  |  |  |  |  |  |  |
|  | nHeM | 6251.273 | 31.734 | 676.9093543 |  |  |  |  |  |  |  |
|  | nHeF | 5831.87 | 35.347 | 678.0730274 |  |  |  |  |  |  |  |
| Left_TemporalOccipitalFusiform@25858 | HeM | 2776.95 | 4.657 | 450.3827051 | 0.009 | < 0.001 | 0.041 | 0.097 | 0.63 | 0.10 | -0.09 |
|  | HeF | 2494.134 | 4.411 | 449.5542664 |  |  |  |  |  |  |  |
|  | nHeM | 2732.909 | 21.068 | 449.3957987 |  |  |  |  |  |  |  |
|  | nHeF | 2533.711 | 23.468 | 450.1942968 |  |  |  |  |  |  |  |
| L_planum_polare@25868 | HeM | 1423.463 | 2.084 | 201.5455352 | 0.01 | < 0.001 | 0.379 | 0.007 | 0.41 | 0.04 | -0.14 |
|  | HeF | 1340.644 | 1.974 | 201.1834327 |  |  |  |  |  |  |  |
|  | nHeM | 1414.969 | 9.428 | 201.1061131 |  |  |  |  |  |  |  |
|  | nHeF | 1369.3 | 10.501 | 201.4441073 |  |  |  |  |  |  |  |
| L_temp_pole@25796 | HeM | 10073.229 | 11.806 | 1141.768997 | 0.011 | < 0.001 | 0.179 | 0.029 | 0.92 | 0.06 | -0.12 |
|  | HeF | 9019.125 | 11.181 | 1139.529869 |  |  |  |  |  |  |  |
|  | nHeM | 9999.66 | 53.41 | 1139.274236 |  |  |  |  |  |  |  |
|  | nHeF | 9151.373 | 59.492 | 1141.254436 |  |  |  |  |  |  |  |
| R_mid_temp_ant@25803 | HeM | 1712.341 | 3.139 | 303.5755447 | 0.012 | < 0.001 | 0.015 | 0.241 | 0.57 | 0.12 | -0.06 |
|  | HeF | 1540.894 | 2.973 | 302.9981487 |  |  |  |  |  |  |  |
|  | nHeM | 1676.926 | 14.2 | 302.8963519 |  |  |  |  |  |  |  |
|  | nHeF | 1559.771 | 15.817 | 303.4226688 |  |  |  |  |  |  |  |
| L_lat_occip_sup@25824 | HeM | 16791.96 | 22.435 | 2169.709253 | 0.012 | < 0.001 | 0.078 | 0.071 | 0.39 | 0.08 | -0.10 |
|  | HeF | 15942.896 | 21.247 | 2165.422693 |  |  |  |  |  |  |  |
|  | nHeM | 16608.88 | 101.49 | 2164.855687 |  |  |  |  |  |  |  |
|  | nHeF | 16150.736 | 113.047 | 2168.617465 |  |  |  |  |  |  |  |
| Left_Precuneus@25842 | HeM | 10605.481 | 13.302 | 1286.448517 | 0.012 | < 0.001 | 0.06 | 0.091 | 0.72 | 0.09 | -0.09 |
|  | HeF | 9680.673 | 12.597 | 1283.843821 |  |  |  |  |  |  |  |
|  | nHeM | 10489.637 | 60.175 | 1283.576618 |  |  |  |  |  |  |  |
|  | nHeF | 9795.791 | 67.027 | 1285.800798 |  |  |  |  |  |  |  |
| R_temp_pole@25797 | HeM | 9967.02 | 11.629 | 1124.651166 | 0.013 | < 0.001 | 0.068 | 0.092 | 0.83 | 0.09 | -0.09 |
|  | HeF | 9033.394 | 11.013 | 1122.407875 |  |  |  |  |  |  |  |
|  | nHeM | 9868.683 | 52.606 | 1122.12433 |  |  |  |  |  |  |  |
|  | nHeF | 9133.769 | 58.597 | 1124.085359 |  |  |  |  |  |  |  |
| L_lat_occip_inf@25826 | HeM | 7556.213 | 11.83 | 1144.090058 | 0.013 | < 0.001 | 0.029 | 0.173 | 0.54 | 0.10 | -0.07 |
|  | HeF | 6941.738 | 11.203 | 1141.772035 |  |  |  |  |  |  |  |
|  | nHeM | 7436.273 | 53.515 | 1141.513963 |  |  |  |  |  |  |  |
|  | nHeF | 7024.264 | 59.608 | 1143.479702 |  |  |  |  |  |  |  |
| Right_Thalamus_vol@25012 | HeM | 7921.055 | 6.42 | 620.8840384 | 0.014 | < 0.001 | 0.032 | 0.167 | 1.03 | 0.10 | -0.07 |
|  | HeF | 7282.876 | 6.08 | 619.6531262 |  |  |  |  |  |  |  |
|  | nHeM | 7857.35 | 29.043 | 619.5083626 |  |  |  |  |  |  |  |
|  | nHeF | 7328.308 | 32.35 | 620.5805991 |  |  |  |  |  |  |  |
| L_postcentral@25814 | HeM | 11552.361 | 14.655 | 1417.298377 | 0.018 | < 0.001 | 0.485 | 0.01 | 0.34 | 0.03 | -0.14 |
|  | HeF | 11077.685 | 13.879 | 1414.500944 |  |  |  |  |  |  |  |
|  | nHeM | 11504.954 | 66.298 | 1414.184672 |  |  |  |  |  |  |  |
|  | nHeF | 11269.977 | 73.847 | 1416.631082 |  |  |  |  |  |  |  |
| R_mid_temp_post@25805 | HeM | 5718.828 | 8.518 | 823.7835264 | 0.019 | < 0.001 | 0.016 | 0.328 | 0.47 | 0.12 | -0.05 |
|  | HeF | 5334.868 | 8.067 | 822.1614752 |  |  |  |  |  |  |  |
|  | nHeM | 5623.456 | 38.532 | 821.9156501 |  |  |  |  |  |  |  |
|  | nHeF | 5377.543 | 42.92 | 823.3483559 |  |  |  |  |  |  |  |
| Left_Thalamus_vol@25011 | HeM | 8111.784 | 6.689 | 646.8992731 | 0.02 | < 0.001 | 0.02 | 0.305 | 0.98 | 0.11 | -0.05 |
|  | HeF | 7480.345 | 6.334 | 645.5399509 |  |  |  |  |  |  |  |
|  | nHeM | 8039.707 | 30.258 | 645.4251983 |  |  |  |  |  |  |  |
|  | nHeF | 7515.472 | 33.704 | 646.5548226 |  |  |  |  |  |  |  |
| L_Caudate@25013 | HeM | 3532.721 | 4.089 | 395.4509086 | 0.023 | < 0.001 | 0.05 | 0.202 | 0.70 | 0.09 | -0.07 |
|  | HeF | 3254.589 | 3.873 | 394.723118 |  |  |  |  |  |  |  |
|  | nHeM | 3495.485 | 18.499 | 394.5971559 |  |  |  |  |  |  |  |
|  | nHeF | 3281.316 | 20.606 | 395.2916175 |  |  |  |  |  |  |  |
| L_mid_temp_post@25804 | HeM | 5579.572 | 8.578 | 829.586181 | 0.024 | < 0.001 | 0.204 | 0.058 | 0.57 | 0.06 | -0.10 |
|  | HeF | 5103.451 | 8.124 | 827.9707232 |  |  |  |  |  |  |  |
|  | nHeM | 5529.065 | 38.806 | 827.7602699 |  |  |  |  |  |  |  |
|  | nHeF | 5186.809 | 43.224 | 829.1800871 |  |  |  |  |  |  |  |
| Right_Paracingulate@25837 | HeM | 5912.277 | 8.111 | 784.4221863 | 0.025 | < 0.001 | 0.183 | 0.07 | 0.52 | 0.06 | -0.10 |
|  | HeF | 5501.367 | 7.681 | 782.821655 |  |  |  |  |  |  |  |
|  | nHeM | 5862.16 | 36.693 | 782.6884395 |  |  |  |  |  |  |  |
|  | nHeF | 5576.718 | 40.871 | 784.0417208 |  |  |  |  |  |  |  |
| R_mid_front_g@25789 | HeM | 10022.937 | 16.864 | 1630.932776 | 0.027 | < 0.001 | 0.17 | 0.082 | 0.54 | 0.07 | -0.09 |
|  | HeF | 9137.123 | 15.971 | 1627.710539 |  |  |  |  |  |  |  |
|  | nHeM | 9915.586 | 76.291 | 1627.342647 |  |  |  |  |  |  |  |
|  | nHeF | 9287.406 | 84.978 | 1630.160685 |  |  |  |  |  |  |  |
| L_Cingulate_post@25840 | HeM | 5580.445 | 7.084 | 685.1000823 | 0.028 | < 0.001 | 0.275 | 0.048 | 1.03 | 0.05 | -0.10 |
|  | HeF | 4874.95 | 6.709 | 683.7586881 |  |  |  |  |  |  |  |
|  | nHeM | 5544.604 | 32.047 | 683.5858725 |  |  |  |  |  |  |  |
|  | nHeF | 4946.675 | 35.696 | 684.7680082 |  |  |  |  |  |  |  |
| R_Cingulate_post@25841 | HeM | 5815.897 | 7.308 | 706.763326 | 0.029 | < 0.001 | 0.407 | 0.028 | 0.99 | 0.04 | -0.12 |
|  | HeF | 5119.63 | 6.921 | 705.3650142 |  |  |  |  |  |  |  |
|  | nHeM | 5787.798 | 33.061 | 705.2152317 |  |  |  |  |  |  |  |
|  | nHeF | 5201.739 | 36.826 | 706.4451667 |  |  |  |  |  |  |  |
| L_mid_temp_ant@25802 | HeM | 1972.167 | 3.824 | 369.8225176 | 0.03 | < 0.001 | 0.016 | 0.448 | 0.69 | 0.12 | -0.04 |
|  | HeF | 1716.157 | 3.622 | 369.1420433 |  |  |  |  |  |  |  |
|  | nHeM | 1929.644 | 17.301 | 369.0429426 |  |  |  |  |  |  |  |
|  | nHeF | 1731.035 | 19.271 | 369.6818771 |  |  |  |  |  |  |  |
| R_lat_occip_sup@25825 | HeM | 16295.465 | 22.31 | 2157.620389 | 0.031 | < 0.001 | 0.19 | 0.085 | 0.57 | 0.06 | -0.09 |
|  | HeF | 15069.48 | 21.128 | 2153.294614 |  |  |  |  |  |  |  |
|  | nHeM | 16159.773 | 100.926 | 2152.825156 |  |  |  |  |  |  |  |
|  | nHeF | 15266.503 | 112.418 | 2156.551153 |  |  |  |  |  |  |  |
| Left_FrontalOrbital@25846 | HeM | 6966.156 | 7.739 | 748.445728 | 0.032 | < 0.001 | 0.058 | 0.243 | 0.86 | 0.09 | -0.06 |
|  | HeF | 6326.71 | 7.329 | 746.9470003 |  |  |  |  |  |  |  |
|  | nHeM | 6898.243 | 35.009 | 746.7674918 |  |  |  |  |  |  |  |
|  | nHeF | 6373.032 | 38.995 | 748.053801 |  |  |  |  |  |  |  |
| Right_TemporalOccipitalFusiform@25859 | HeM | 3567.506 | 5.735 | 554.6370655 | 0.033 | < 0.001 | 0.422 | 0.032 | 0.75 | 0.04 | -0.11 |
|  | HeF | 3154.293 | 5.431 | 553.5092316 |  |  |  |  |  |  |  |
|  | nHeM | 3546.134 | 25.943 | 553.3831026 |  |  |  |  |  |  |  |
|  | nHeF | 3217.296 | 28.898 | 554.3597574 |  |  |  |  |  |  |  |
| Right_OccipitalFusiform@25861 | HeM | 3886.683 | 5.847 | 565.4686874 | 0.043 | < 0.001 | 0.474 | 0.038 | 0.54 | 0.03 | -0.11 |
|  | HeF | 3580.528 | 5.538 | 564.4143113 |  |  |  |  |  |  |  |
|  | nHeM | 3867.283 | 26.452 | 564.2404437 |  |  |  |  |  |  |  |
|  | nHeF | 3642.815 | 29.464 | 565.21752 |  |  |  |  |  |  |  |
| Right_Subcallosal@25835 | HeM | 2888.135 | 3.527 | 341.0993775 | 0.045 | < 0.001 | 0.179 | 0.137 | 0.92 | 0.06 | -0.08 |
|  | HeF | 2575.31 | 3.341 | 340.5034695 |  |  |  |  |  |  |  |
|  | nHeM | 2866.159 | 15.958 | 340.3957735 |  |  |  |  |  |  |  |
|  | nHeF | 2602.171 | 17.775 | 340.9836213 |  |  |  |  |  |  |  |
| R_Precentral@25795 | HeM | 14218.98 | 16.474 | 1593.215522 | 0.046 | < 0.001 | 0.28 | 0.087 | 0.62 | 0.05 | -0.09 |
|  | HeF | 13235.584 | 15.601 | 1590.001385 |  |  |  |  |  |  |  |
|  | nHeM | 14136.429 | 74.523 | 1589.629918 |  |  |  |  |  |  |  |
|  | nHeF | 13380.215 | 83.009 | 1592.388716 |  |  |  |  |  |  |  |
| R_supra_marginal_post@25821 | HeM | 5702.429 | 10.324 | 998.4434288 | 0.046 | < 0.001 | 0.505 | 0.037 | 0.43 | 0.03 | -0.11 |
|  | HeF | 5271.482 | 9.777 | 996.4389169 |  |  |  |  |  |  |  |
|  | nHeM | 5670.492 | 46.702 | 996.1877061 |  |  |  |  |  |  |  |
|  | nHeF | 5381.543 | 52.02 | 997.9166234 |  |  |  |  |  |  |  |
| Right_Lingual@25853 | HeM | 7444.13 | 8.485 | 820.5920664 | 0.046 | < 0.001 | 0.26 | 0.096 | 1.06 | 0.05 | -0.09 |
|  | HeF | 6576.021 | 8.036 | 819.0020596 |  |  |  |  |  |  |  |
|  | nHeM | 7399.857 | 38.384 | 818.7587022 |  |  |  |  |  |  |  |
|  | nHeF | 6648.419 | 42.755 | 820.1831071 |  |  |  |  |  |  |  |
| Right_FrontalMedial@25831 | HeM | 1970.123 | 3.23 | 312.3762374 | 0.046 | < 0.001 | 0.051 | 0.355 | 0.46 | 0.09 | -0.05 |
|  | HeF | 1828.054 | 3.059 | 311.7629791 |  |  |  |  |  |  |  |
|  | nHeM | 1940.935 | 14.613 | 311.705943 |  |  |  |  |  |  |  |
|  | nHeF | 1843.375 | 16.277 | 312.2469988 |  |  |  |  |  |  |  |
| R_insula@25785 | HeM | 6568.794 | 6.269 | 606.2806911 | 0.047 | < 0.001 | 0.37 | 0.063 | 0.91 | 0.04 | -0.10 |
|  | HeF | 6015.061 | 5.937 | 605.0790477 |  |  |  |  |  |  |  |
|  | nHeM | 6542.738 | 28.36 | 604.9394747 |  |  |  |  |  |  |  |
|  | nHeF | 6074.866 | 31.589 | 605.982088 |  |  |  |  |  |  |  |
| L_insula@25784 | HeM | 6573.088 | 6.351 | 614.2109857 | 0.05 | < 0.001 | 0.805 | 0.016 | 0.88 | 0.01 | -0.13 |
|  | HeF | 6033.751 | 6.015 | 613.0285451 |  |  |  |  |  |  |  |
|  | nHeM | 6565.829 | 28.73 | 612.8318444 |  |  |  |  |  |  |  |
|  | nHeF | 6112.37 | 32.002 | 613.9048016 |  |  |  |  |  |  |  |
| L_sup_parietal@25816 | HeM | 5293.673 | 9.386 | 907.7285958 | 0.054 | < 0.001 | 0.347 | 0.081 | 0.33 | 0.05 | -0.09 |
|  | HeF | 4992.906 | 8.889 | 905.9369472 |  |  |  |  |  |  |  |
|  | nHeM | 5252.746 | 42.46 | 905.7027537 |  |  |  |  |  |  |  |
|  | nHeF | 5076.808 | 47.295 | 907.2754076 |  |  |  |  |  |  |  |
| L_planum_temporale@25872 | HeM | 2251.823 | 4.551 | 440.1313488 | 0.054 | < 0.001 | 0.062 | 0.366 | 0.87 | 0.09 | -0.05 |
|  | HeF | 1870.731 | 4.31 | 439.2606865 |  |  |  |  |  |  |  |
|  | nHeM | 2212.394 | 20.586 | 439.1143874 |  |  |  |  |  |  |  |
|  | nHeF | 1891.805 | 22.93 | 439.8736673 |  |  |  |  |  |  |  |
| L_Putamen@25015 | HeM | 5063.408 | 5.366 | 518.9507399 | 0.064 | < 0.001 | 0.171 | 0.209 | 0.98 | 0.07 | -0.07 |
|  | HeF | 4555.811 | 5.082 | 517.9403269 |  |  |  |  |  |  |  |
|  | nHeM | 5029.368 | 24.274 | 517.7821159 |  |  |  |  |  |  |  |
|  | nHeF | 4590.315 | 27.038 | 518.6787709 |  |  |  |  |  |  |  |
| Left_OccipitalFusiform@25860 | HeM | 4109.508 | 5.915 | 572.0450292 | 0.066 | < 0.001 | 0.622 | 0.043 | 0.62 | 0.02 | -0.11 |
|  | HeF | 3756.265 | 5.601 | 570.8350592 |  |  |  |  |  |  |  |
|  | nHeM | 4095.994 | 26.756 | 570.7249853 |  |  |  |  |  |  |  |
|  | nHeF | 3817.602 | 29.803 | 571.7206676 |  |  |  |  |  |  |  |
| R_postcentral@25815 | HeM | 10694.001 | 14.192 | 1372.521226 | 0.069 | < 0.001 | 0.303 | 0.13 | 0.25 | 0.05 | -0.08 |
|  | HeF | 10347.448 | 13.44 | 1369.759542 |  |  |  |  |  |  |  |
|  | nHeM | 10626.149 | 64.201 | 1369.454133 |  |  |  |  |  |  |  |
|  | nHeF | 10457.626 | 71.511 | 1371.818832 |  |  |  |  |  |  |  |
| Left_Subcallosal@25834 | HeM | 3113.796 | 3.957 | 382.6850686 | 0.075 | < 0.001 | 0.111 | 0.342 | 0.86 | 0.08 | -0.05 |
|  | HeF | 2783.736 | 3.747 | 381.8816223 |  |  |  |  |  |  |  |
|  | nHeM | 3084.55 | 17.899 | 381.7987185 |  |  |  |  |  |  |  |
|  | nHeF | 2803.002 | 19.937 | 382.4579723 |  |  |  |  |  |  |  |
| L_occip_pole@25876 | HeM | 8961.941 | 13.59 | 1314.301259 | 0.079 | < 0.001 | 0.036 | 0.644 | 0.60 | 0.10 | -0.02 |
|  | HeF | 8168.629 | 12.871 | 1311.768978 |  |  |  |  |  |  |  |
|  | nHeM | 8829.622 | 61.481 | 1311.43455 |  |  |  |  |  |  |  |
|  | nHeF | 8200.801 | 68.482 | 1313.712538 |  |  |  |  |  |  |  |
| Left_Lingual@25852 | HeM | 6973.49 | 8.11 | 784.3254754 | 0.083 | < 0.001 | 0.747 | 0.009 | 1.09 | -0.02 | -0.14 |
|  | HeF | 6122.331 | 7.681 | 782.821655 |  |  |  |  |  |  |  |
|  | nHeM | 6985.608 | 36.69 | 782.6244473 |  |  |  |  |  |  |  |
|  | nHeF | 6231.31 | 40.868 | 783.9841708 |  |  |  |  |  |  |  |
| L_Precentral@25794 | HeM | 14587.685 | 16.764 | 1621.261685 | 0.084 | < 0.001 | 0.163 | 0.286 | 0.62 | 0.07 | -0.06 |
|  | HeF | 13583.487 | 15.876 | 1618.028459 |  |  |  |  |  |  |  |
|  | nHeM | 14479.326 | 75.835 | 1617.615834 |  |  |  |  |  |  |  |
|  | nHeF | 13675.029 | 84.471 | 1620.434738 |  |  |  |  |  |  |  |
| Right_InferiorTemporal_toc@25813 | HeM | 4166.278 | 7.326 | 708.5041224 | 0.095 | < 0.001 | 0.031 | 0.773 | 0.81 | 0.10 | -0.02 |
|  | HeF | 3590.844 | 6.938 | 707.097597 |  |  |  |  |  |  |  |
|  | nHeM | 4092.835 | 33.14 | 706.9003593 |  |  |  |  |  |  |  |
|  | nHeF | 3601.658 | 36.914 | 708.1332994 |  |  |  |  |  |  |  |
| R_Putamen@25016 | HeM | 5122.045 | 5.256 | 508.3125399 | 0.107 | < 0.001 | 0.354 | 0.184 | 1.03 | 0.04 | -0.07 |
|  | HeF | 4597.138 | 4.978 | 507.3409971 |  |  |  |  |  |  |  |
|  | nHeM | 5099.45 | 23.779 | 507.2234051 |  |  |  |  |  |  |  |
|  | nHeF | 4632.93 | 26.487 | 508.1087582 |  |  |  |  |  |  |  |
| R_central_opercular_cortex@25865 | HeM | 3909.584 | 5.78 | 558.9890564 | 0.114 | < 0.001 | 0.469 | 0.141 | 0.58 | 0.03 | -0.08 |
|  | HeF | 3583.974 | 5.474 | 557.8916468 |  |  |  |  |  |  |  |
|  | nHeM | 3890.197 | 26.146 | 557.7132406 |  |  |  |  |  |  |  |
|  | nHeF | 3627.569 | 29.123 | 558.6760058 |  |  |  |  |  |  |  |
| L_supra_marginal_ant@25818 | HeM | 3329.992 | 6.646 | 642.740704 | 0.117 | < 0.001 | 0.047 | 0.758 | 0.38 | 0.10 | -0.02 |
|  | HeF | 3089.083 | 6.294 | 641.4632856 |  |  |  |  |  |  |  |
|  | nHeM | 3268.692 | 30.065 | 641.3083676 |  |  |  |  |  |  |  |
|  | nHeF | 3099.583 | 33.489 | 642.4304075 |  |  |  |  |  |  |  |
| Right_Cuneus@25845 | HeM | 2462.17 | 4.654 | 450.0925724 | 0.125 | < 0.001 | 0.118 | 0.516 | 0.55 | 0.08 | -0.03 |
|  | HeF | 2215.896 | 4.407 | 449.1465999 |  |  |  |  |  |  |  |
|  | nHeM | 2428.404 | 21.053 | 449.0758378 |  |  |  |  |  |  |  |
|  | nHeF | 2231.39 | 23.45 | 449.8489969 |  |  |  |  |  |  |  |
| R_sup_temp_ant@25799 | HeM | 1548.351 | 2.998 | 289.9393064 | 0.139 | < 0.001 | 0.139 | 0.515 | 0.49 | 0.07 | -0.03 |
|  | HeF | 1405.658 | 2.839 | 289.3413199 |  |  |  |  |  |  |  |
|  | nHeM | 1527.774 | 13.561 | 289.2660161 |  |  |  |  |  |  |  |
|  | nHeF | 1415.657 | 15.106 | 289.783324 |  |  |  |  |  |  |  |
| L_parietal_operculum@25866 | HeM | 2225.17 | 4.431 | 428.5260396 | 0.141 | < 0.001 | 0.286 | 0.31 | 0.72 | 0.05 | -0.05 |
|  | HeF | 1915.099 | 4.197 | 427.744107 |  |  |  |  |  |  |  |
|  | nHeM | 2203.261 | 20.046 | 427.5957937 |  |  |  |  |  |  |  |
|  | nHeF | 1938.135 | 22.329 | 428.3444883 |  |  |  |  |  |  |  |
| L_sup_temp_post@25800 | HeM | 2723.902 | 4.818 | 465.9531616 | 0.15 | < 0.001 | 0.139 | 0.551 | 0.54 | 0.07 | -0.03 |
|  | HeF | 2471.887 | 4.563 | 465.0455945 |  |  |  |  |  |  |  |
|  | nHeM | 2690.837 | 21.795 | 464.9032387 |  |  |  |  |  |  |  |
|  | nHeF | 2486.615 | 24.277 | 465.7136076 |  |  |  |  |  |  |  |
| L_mid_front_g@25788 | HeM | 10548.794 | 17.534 | 1695.729086 | 0.164 | < 0.001 | 0.569 | 0.175 | 0.53 | 0.03 | -0.07 |
|  | HeF | 9654.518 | 16.605 | 1692.325684 |  |  |  |  |  |  |  |
|  | nHeM | 10502.509 | 79.318 | 1691.910763 |  |  |  |  |  |  |  |
|  | nHeF | 9776.457 | 88.35 | 1694.84686 |  |  |  |  |  |  |  |
| Left_InferiorTemporal_post@25810 | HeM | 4416.062 | 7.678 | 742.5463625 | 0.177 | < 0.001 | 0.113 | 0.703 | 0.67 | 0.08 | -0.02 |
|  | HeF | 3916.478 | 7.271 | 741.0358356 |  |  |  |  |  |  |  |
|  | nHeM | 4359.575 | 34.732 | 740.8588799 |  |  |  |  |  |  |  |
|  | nHeF | 3931.483 | 38.687 | 742.1453366 |  |  |  |  |  |  |  |
| R_Accumbens@25024 | HeM | 412.154 | 1.049 | 101.449744 | 0.185 | < 0.001 | 0.725 | 0.035 | 0.30 | 0.02 | 0.11 |
|  | HeF | 381.916 | 0.993 | 101.2032162 |  |  |  |  |  |  |  |
|  | nHeM | 410.44 | 4.745 | 101.2143091 |  |  |  |  |  |  |  |
|  | nHeF | 370.613 | 5.285 | 101.3838784 |  |  |  |  |  |  |  |
| R_angular_g@25823 | HeM | 5794.778 | 11.179 | 1081.131256 | 0.198 | < 0.001 | 0.853 | 0.118 | 0.48 | 0.01 | -0.08 |
|  | HeF | 5277.908 | 10.587 | 1078.991389 |  |  |  |  |  |  |  |
|  | nHeM | 5785.172 | 50.573 | 1078.758958 |  |  |  |  |  |  |  |
|  | nHeF | 5367.561 | 56.332 | 1080.635125 |  |  |  |  |  |  |  |
| L_Amygdala@25021 | HeM | 1345.105 | 2.46 | 237.9088372 | 0.204 | < 0.001 | 0.175 | 0.63 | 0.58 | 0.07 | -0.03 |
|  | HeF | 1206.674 | 2.329 | 237.3638373 |  |  |  |  |  |  |  |
|  | nHeM | 1329.627 | 11.127 | 237.3470217 |  |  |  |  |  |  |  |
|  | nHeF | 1212.739 | 12.394 | 237.7581436 |  |  |  |  |  |  |  |
| Left_InferiorTemporal_ant@25808 | HeM | 1537.835 | 3.101 | 299.9005301 | 0.205 | < 0.001 | 0.222 | 0.548 | 0.60 | 0.06 | -0.03 |
|  | HeF | 1359.164 | 2.937 | 299.3291499 |  |  |  |  |  |  |  |
|  | nHeM | 1520.277 | 14.03 | 299.270128 |  |  |  |  |  |  |  |
|  | nHeF | 1368.704 | 15.628 | 299.7970202 |  |  |  |  |  |  |  |
| Right_Cingulate_ant@25839 | HeM | 5936.803 | 11.75 | 1136.353186 | 0.206 | < 0.001 | 0.519 | 0.022 | 0.52 | -0.03 | -0.12 |
|  | HeF | 5348.927 | 11.128 | 1134.128288 |  |  |  |  |  |  |  |
|  | nHeM | 5971.96 | 53.154 | 1133.81357 |  |  |  |  |  |  |  |
|  | nHeF | 5486.633 | 59.207 | 1135.787188 |  |  |  |  |  |  |  |
| Right_InferiorTemporal_post@25811 | HeM | 4426.111 | 7.443 | 719.8192988 | 0.206 | < 0.001 | 0.209 | 0.573 | 0.60 | 0.06 | -0.03 |
|  | HeF | 3993.934 | 7.049 | 718.4103432 |  |  |  |  |  |  |  |
|  | nHeM | 4382.705 | 33.672 | 718.2483071 |  |  |  |  |  |  |  |
|  | nHeF | 4015.407 | 37.506 | 719.4898285 |  |  |  |  |  |  |  |
| L_Pars_Triangularis@25790 | HeM | 2535.16 | 5.865 | 567.2094837 | 0.22 | < 0.001 | 0.44 | 0.341 | 0.30 | 0.04 | -0.05 |
|  | HeF | 2367.243 | 5.554 | 566.0449774 |  |  |  |  |  |  |  |
|  | nHeM | 2514.183 | 26.531 | 565.9255713 |  |  |  |  |  |  |  |
|  | nHeF | 2395.872 | 29.552 | 566.9056527 |  |  |  |  |  |  |  |
| L_sup_front_g@25786 | HeM | 11505.433 | 17.38 | 1680.835606 | 0.228 | < 0.001 | 0.481 | 0.325 | 0.52 | 0.03 | -0.05 |
|  | HeF | 10635.227 | 16.46 | 1677.547773 |  |  |  |  |  |  |  |
|  | nHeM | 11448.616 | 78.626 | 1677.149899 |  |  |  |  |  |  |  |
|  | nHeF | 10722.936 | 87.579 | 1680.056516 |  |  |  |  |  |  |  |
| L_Heschl_h1h2@25870 | HeM | 1269.018 | 2.563 | 247.8700608 | 0.245 | < 0.001 | 0.118 | 0.88 | 0.59 | 0.07 | -0.01 |
|  | HeF | 1123.193 | 2.427 | 247.3516673 |  |  |  |  |  |  |  |
|  | nHeM | 1250.456 | 11.594 | 247.3084721 |  |  |  |  |  |  |  |
|  | nHeF | 1125.174 | 12.914 | 247.7334732 |  |  |  |  |  |  |  |
| L_angular_g@25822 | HeM | 4228.811 | 7.917 | 765.6602698 | 0.256 | < 0.001 | 0.066 | 0.895 | 0.37 | 0.09 | 0.01 |
|  | HeF | 3947.601 | 7.498 | 764.1709112 |  |  |  |  |  |  |  |
|  | nHeM | 4161.408 | 35.815 | 763.9600594 |  |  |  |  |  |  |  |
|  | nHeF | 3942.231 | 39.893 | 765.2804278 |  |  |  |  |  |  |  |
| R_Heschl_h1h2@25871 | HeM | 1080.522 | 1.937 | 187.3290315 | 0.262 | < 0.001 | 0.743 | 0.071 | 0.57 | -0.02 | -0.10 |
|  | HeF | 974.424 | 1.834 | 186.9151042 |  |  |  |  |  |  |  |
|  | nHeM | 1083.465 | 8.763 | 186.9211783 |  |  |  |  |  |  |  |
|  | nHeF | 992.342 | 9.76 | 187.2292627 |  |  |  |  |  |  |  |
| R_pars_operc@25793 | HeM | 2555.165 | 5.291 | 511.6974217 | 0.278 | < 0.001 | 0.404 | 0.481 | 0.30 | 0.04 | -0.04 |
|  | HeF | 2400.916 | 5.011 | 510.7042459 |  |  |  |  |  |  |  |
|  | nHeM | 2534.682 | 23.937 | 510.5936603 |  |  |  |  |  |  |  |
|  | nHeF | 2420.034 | 26.663 | 511.4850236 |  |  |  |  |  |  |  |
| R_Pars_Triangularis@25791 | HeM | 2347.764 | 5.053 | 488.6802253 | 0.282 | < 0.001 | 0.367 | 0.528 | 0.35 | 0.04 | -0.03 |
|  | HeF | 2179.128 | 4.785 | 487.671087 |  |  |  |  |  |  |  |
|  | nHeM | 2326.635 | 22.858 | 487.5778037 |  |  |  |  |  |  |  |
|  | nHeF | 2195.473 | 25.461 | 488.4266657 |  |  |  |  |  |  |  |
| Left_Cuneus@25844 | HeM | 2042.063 | 3.984 | 385.2962631 | 0.292 | < 0.001 | 0.342 | 0.578 | 0.40 | 0.05 | -0.03 |
|  | HeF | 1888.005 | 3.773 | 384.5314548 |  |  |  |  |  |  |  |
|  | nHeM | 2024.49 | 18.024 | 384.4650596 |  |  |  |  |  |  |  |
|  | nHeF | 1899.367 | 20.076 | 385.1244546 |  |  |  |  |  |  |  |
| R_front_operculum@25863 | HeM | 1400.358 | 2.401 | 232.2028935 | 0.297 | < 0.001 | 0.393 | 0.528 | 0.49 | 0.04 | -0.03 |
|  | HeF | 1287.638 | 2.274 | 231.7584225 |  |  |  |  |  |  |  |
|  | nHeM | 1390.848 | 10.861 | 231.6730478 |  |  |  |  |  |  |  |
|  | nHeF | 1295.403 | 12.098 | 232.0798791 |  |  |  |  |  |  |  |
| Right_Parahippocampal_ant@25849 | HeM | 3213.727 | 4.324 | 418.1779723 | 0.298 | < 0.001 | 0.831 | 0.227 | 0.94 | 0.01 | -0.06 |
|  | HeF | 2820.226 | 4.095 | 417.3486105 |  |  |  |  |  |  |  |
|  | nHeM | 3209.439 | 19.561 | 417.2503901 |  |  |  |  |  |  |  |
|  | nHeF | 2846.994 | 21.789 | 417.9854922 |  |  |  |  |  |  |  |
| R_planum_polare@25869 | HeM | 1508.095 | 2.144 | 207.3481898 | 0.318 | < 0.001 | 0.834 | 0.248 | 0.45 | 0.01 | -0.06 |
|  | HeF | 1415.509 | 2.03 | 206.8907642 |  |  |  |  |  |  |  |
|  | nHeM | 1506.01 | 9.698 | 206.8654099 |  |  |  |  |  |  |  |
|  | nHeF | 1428.193 | 10.802 | 207.2182885 |  |  |  |  |  |  |  |
| R_sup_front_g@25787 | HeM | 10041.912 | 15.934 | 1540.991631 | 0.337 | < 0.001 | 0.985 | 0.19 | 0.54 | 0.00 | -0.07 |
|  | HeF | 9205.131 | 15.09 | 1537.921986 |  |  |  |  |  |  |  |
|  | nHeM | 10043.3 | 72.081 | 1537.540278 |  |  |  |  |  |  |  |
|  | nHeF | 9312.041 | 80.289 | 1540.210069 |  |  |  |  |  |  |  |
| Right_TemporalFusiform_post@25857 | HeM | 3424.581 | 4.498 | 435.0056706 | 0.338 | < 0.001 | 0.198 | 0.902 | 0.77 | 0.06 | -0.01 |
|  | HeF | 3091.312 | 4.26 | 434.1648549 |  |  |  |  |  |  |  |
|  | nHeM | 3397.72 | 20.348 | 434.0376738 |  |  |  |  |  |  |  |
|  | nHeF | 3094.138 | 22.665 | 434.7900859 |  |  |  |  |  |  |  |
| R_supra_marginal_ant@25819 | HeM | 3228.149 | 6.3 | 609.2787293 | 0.394 | < 0.001 | 0.579 | 0.519 | 0.27 | 0.03 | -0.03 |
|  | HeF | 3066.483 | 5.967 | 608.1365467 |  |  |  |  |  |  |  |
|  | nHeM | 3211.957 | 28.502 | 607.9684382 |  |  |  |  |  |  |  |
|  | nHeF | 3087.313 | 31.748 | 609.0322368 |  |  |  |  |  |  |  |
| Right_InferiorTemporal_ant@25809 | HeM | 1593.744 | 2.827 | 273.4017409 | 0.394 | < 0.001 | 0.587 | 0.511 | 0.61 | 0.03 | -0.03 |
|  | HeF | 1427.384 | 2.678 | 272.9327421 |  |  |  |  |  |  |  |
|  | nHeM | 1586.629 | 12.791 | 272.8413547 |  |  |  |  |  |  |  |
|  | nHeF | 1436.9 | 14.247 | 273.3048469 |  |  |  |  |  |  |  |
| R_occip_pole@25877 | HeM | 8736.919 | 12.893 | 1246.893755 | 0.396 | < 0.001 | 0.28 | 0.868 | 0.85 | 0.05 | -0.01 |
|  | HeF | 7676.6 | 12.21 | 1244.402084 |  |  |  |  |  |  |  |
|  | nHeM | 8672.371 | 58.325 | 1244.114769 |  |  |  |  |  |  |  |
|  | nHeF | 7687.571 | 64.966 | 1246.263963 |  |  |  |  |  |  |  |
| Left_FrontalMedial@25830 | HeM | 1976.277 | 3.195 | 308.9913556 | 0.402 | < 0.001 | 0.466 | 0.64 | 0.50 | 0.03 | -0.02 |
|  | HeF | 1823.105 | 3.025 | 308.2978136 |  |  |  |  |  |  |  |
|  | nHeM | 1965.476 | 14.452 | 308.2716956 |  |  |  |  |  |  |  |
|  | nHeF | 1830.757 | 16.098 | 308.8131834 |  |  |  |  |  |  |  |
| Right_Parahippocampal_post@25851 | HeM | 1427.32 | 1.769 | 171.0815988 | 0.407 | < 0.001 | 0.552 | 0.564 | 0.52 | 0.03 | -0.03 |
|  | HeF | 1338.925 | 1.676 | 170.8122762 |  |  |  |  |  |  |  |
|  | nHeM | 1422.438 | 8.005 | 170.7524857 |  |  |  |  |  |  |  |
|  | nHeF | 1344.156 | 8.916 | 171.0385354 |  |  |  |  |  |  |  |
| L_supra_marginal_post@25820 | HeM | 4860.793 | 8.556 | 827.458541 | 0.435 | < 0.001 | 0.165 | 0.836 | 0.49 | 0.07 | 0.01 |
|  | HeF | 4459.725 | 8.103 | 825.8304739 |  |  |  |  |  |  |  |
|  | nHeM | 4805.644 | 38.705 | 825.6058662 |  |  |  |  |  |  |  |
|  | nHeF | 4450.644 | 43.112 | 827.0315545 |  |  |  |  |  |  |  |
| Left_TemporalFusiform_ant@25854 | HeM | 1595.728 | 2.417 | 233.7502681 | 0.451 | < 0.001 | 0.669 | 0.531 | 0.70 | 0.02 | -0.03 |
|  | HeF | 1431.18 | 2.289 | 233.287172 |  |  |  |  |  |  |  |
|  | nHeM | 1590.934 | 10.932 | 233.1875295 |  |  |  |  |  |  |  |
|  | nHeF | 1438.936 | 12.177 | 233.5953618 |  |  |  |  |  |  |  |
| R_mid_temp_toc@25807 | HeM | 5039.964 | 9.254 | 894.9627557 | 0.464 | < 0.001 | 0.353 | 0.884 | 0.68 | 0.04 | -0.01 |
|  | HeF | 4433.966 | 8.764 | 893.1973681 |  |  |  |  |  |  |  |
|  | nHeM | 5000.126 | 41.865 | 893.0109699 |  |  |  |  |  |  |  |
|  | nHeF | 4440.901 | 46.633 | 894.5760457 |  |  |  |  |  |  |  |
| R_sup_temp_post@25801 | HeM | 3265.292 | 5.262 | 508.8928053 | 0.497 | < 0.001 | 0.773 | 0.514 | 0.53 | 0.01 | -0.03 |
|  | HeF | 2993.971 | 4.983 | 507.8505802 |  |  |  |  |  |  |  |
|  | nHeM | 3258.262 | 23.804 | 507.7566733 |  |  |  |  |  |  |  |
|  | nHeF | 3011.558 | 26.514 | 508.626708 |  |  |  |  |  |  |  |
| L_mid_temp_toc@25806 | HeM | 3627.182 | 7.834 | 757.6332644 | 0.526 | < 0.001 | 0.528 | 0.154 | 0.34 | -0.03 | -0.08 |
|  | HeF | 3373.351 | 7.419 | 756.1194972 |  |  |  |  |  |  |  |
|  | nHeM | 3650.088 | 35.439 | 755.9397053 |  |  |  |  |  |  |  |
|  | nHeF | 3430.544 | 39.474 | 757.2426142 |  |  |  |  |  |  |  |
| R_Pallidum@25018 | HeM | 1886.418 | 2.351 | 227.367348 | 0.553 | < 0.001 | 0.026 | 0.222 | 0.69 | 0.11 | 0.06 |
|  | HeF | 1730.626 | 2.226 | 226.8664242 |  |  |  |  |  |  |  |
|  | nHeM | 1862.083 | 10.634 | 226.8309723 |  |  |  |  |  |  |  |
|  | nHeF | 1715.912 | 11.845 | 227.2264976 |  |  |  |  |  |  |  |
| Right_TemporalFusiform_ant@25855 | HeM | 1470.606 | 2.206 | 213.3442662 | 0.554 | < 0.001 | 0.615 | 0.733 | 0.82 | 0.02 | -0.02 |
|  | HeF | 1294.818 | 2.089 | 212.9038455 |  |  |  |  |  |  |  |
|  | nHeM | 1465.456 | 9.979 | 212.8593448 |  |  |  |  |  |  |  |
|  | nHeF | 1298.666 | 11.115 | 213.2226695 |  |  |  |  |  |  |  |
| L_pars_operc@25792 | HeM | 2742.577 | 5.346 | 517.0165217 | 0.556 | < 0.001 | 0.548 | 0.181 | 0.33 | -0.03 | -0.07 |
|  | HeF | 2572.141 | 5.063 | 516.0039108 |  |  |  |  |  |  |  |
|  | nHeM | 2757.475 | 24.184 | 515.8623503 |  |  |  |  |  |  |  |
|  | nHeF | 2608.756 | 26.937 | 516.741255 |  |  |  |  |  |  |  |
| L_Accumbens@25023 | HeM | 526.332 | 1.146 | 110.8307022 | 0.559 | < 0.001 | 0.005 | 0.077 | 0.40 | 0.14 | 0.09 |
|  | HeF | 482.305 | 1.086 | 110.681463 |  |  |  |  |  |  |  |
|  | nHeM | 511.32 | 5.186 | 110.6211606 |  |  |  |  |  |  |  |
|  | nHeF | 471.918 | 5.777 | 110.8220748 |  |  |  |  |  |  |  |
| R_Amygdala@25022 | HeM | 1311.294 | 2.744 | 265.3747354 | 0.607 | < 0.001 | 0.055 | 0.015 | 0.53 | -0.09 | -0.13 |
|  | HeF | 1170.112 | 2.598 | 264.7794115 |  |  |  |  |  |  |  |
|  | nHeM | 1335.741 | 12.412 | 264.7570084 |  |  |  |  |  |  |  |
|  | nHeF | 1204.284 | 13.826 | 265.2286666 |  |  |  |  |  |  |  |
| Left_Hippocampus_vol@25019 | HeM | 3913.129 | 4.686 | 453.1873215 | 0.644 | < 0.001 | 0.884 | 0.624 | 0.48 | 0.01 | -0.03 |
|  | HeF | 3694.724 | 4.437 | 452.2040988 |  |  |  |  |  |  |  |
|  | nHeM | 3909.961 | 21.197 | 452.1474628 |  |  |  |  |  |  |  |
|  | nHeF | 3706.478 | 23.611 | 452.9375124 |  |  |  |  |  |  |  |
| R_planum_temporale@25873 | HeM | 1724.025 | 3.01 | 291.0998373 | 0.649 | < 0.001 | 0.454 | 0.949 | 0.78 | -0.04 | 0.00 |
|  | HeF | 1498.519 | 2.851 | 290.5643195 |  |  |  |  |  |  |  |
|  | nHeM | 1734.475 | 13.618 | 290.4818676 |  |  |  |  |  |  |  |
|  | nHeF | 1499.509 | 15.168 | 290.9726902 |  |  |  |  |  |  |  |
| L_Pallidum@25017 | HeM | 1842.296 | 2.361 | 228.3344571 | 0.665 | < 0.001 | 0.215 | 0.59 | 0.69 | 0.06 | 0.03 |
|  | HeF | 1684.537 | 2.236 | 227.8855905 |  |  |  |  |  |  |  |
|  | nHeM | 1828.722 | 10.682 | 227.8548473 |  |  |  |  |  |  |  |
|  | nHeF | 1678.023 | 11.898 | 228.2432139 |  |  |  |  |  |  |  |
| R_sup_parietal@25817 | HeM | 4796.26 | 9.044 | 874.6534648 | 0.677 | < 0.001 | 0.613 | 0.918 | 0.32 | 0.02 | -0.01 |
|  | HeF | 4517.996 | 8.565 | 872.9159582 |  |  |  |  |  |  |  |
|  | nHeM | 4775.066 | 40.912 | 872.6827852 |  |  |  |  |  |  |  |
|  | nHeF | 4522.795 | 45.571 | 874.2033534 |  |  |  |  |  |  |  |
| R_suppl_motor_juxta@25833 | HeM | 2895.058 | 5.651 | 546.5133491 | 0.681 | < 0.001 | 0.446 | 0.892 | 0.40 | 0.04 | 0.01 |
|  | HeF | 2679.369 | 5.351 | 545.355901 |  |  |  |  |  |  |  |
|  | nHeM | 2875.106 | 25.562 | 545.2560949 |  |  |  |  |  |  |  |
|  | nHeF | 2675.444 | 28.473 | 546.2068439 |  |  |  |  |  |  |  |
| Left_TemporalFusiform_post@25856 | HeM | 4195.215 | 5.33 | 515.4691472 | 0.719 | < 0.001 | 0.43 | 0.818 | 0.81 | 0.04 | 0.01 |
|  | HeF | 3775.593 | 5.048 | 514.4751614 |  |  |  |  |  |  |  |
|  | nHeM | 4175.724 | 24.114 | 514.3691993 |  |  |  |  |  |  |  |
|  | nHeF | 3769.305 | 26.86 | 515.2641389 |  |  |  |  |  |  |  |
| R_parietal_operculum@25867 | HeM | 2196.367 | 4.137 | 400.0930323 | 0.803 | < 0.001 | 0.516 | 0.355 | 0.73 | 0.03 | 0.05 |
|  | HeF | 1903.398 | 3.918 | 399.3093665 |  |  |  |  |  |  |  |
|  | nHeM | 2183.897 | 18.716 | 399.2259241 |  |  |  |  |  |  |  |
|  | nHeF | 1883.8 | 20.847 | 399.9147991 |  |  |  |  |  |  |  |
| L_suppl_motor_juxta@25832 | HeM | 2992.658 | 5.674 | 548.7377 | 0.895 | < 0.001 | 0.979 | 0.841 | 0.41 | 0.00 | 0.01 |
|  | HeF | 2768.503 | 5.374 | 547.6999836 |  |  |  |  |  |  |  |
|  | nHeM | 2991.97 | 25.67 | 547.5598136 |  |  |  |  |  |  |  |
|  | nHeF | 2762.667 | 28.593 | 548.508843 |  |  |  |  |  |  |  |
| Left_Parahippocampal_ant@25848 | HeM | 3120.834 | 4.066 | 393.2265577 | 0.9 | < 0.001 | 0.387 | 0.54 | 0.86 | -0.04 | -0.03 |
|  | HeF | 2781.497 | 3.851 | 392.4809521 |  |  |  |  |  |  |  |
|  | nHeM | 3137.128 | 18.393 | 392.3360986 |  |  |  |  |  |  |  |
|  | nHeF | 2794.265 | 20.488 | 393.027985 |  |  |  |  |  |  |  |
| Left_Parahippocampal_post@25850 | HeM | 1767.112 | 2.115 | 204.5435734 | 0.916 | < 0.001 | 0.868 | 0.993 | 0.59 | -0.01 | 0.00 |
|  | HeF | 1647.279 | 2.003 | 204.1390151 |  |  |  |  |  |  |  |
|  | nHeM | 1768.746 | 9.566 | 204.0497537 |  |  |  |  |  |  |  |
|  | nHeF | 1647.368 | 10.655 | 204.3983395 |  |  |  |  |  |  |  |
| Right_Hippocampus_vol@25020 | HeM | 4033.569 | 4.793 | 463.5353888 | 0.96 | < 0.001 | 0.439 | 0.442 | 0.53 | -0.04 | -0.04 |
|  | HeF | 3790.451 | 4.539 | 462.5995954 |  |  |  |  |  |  |  |
|  | nHeM | 4050.776 | 21.682 | 462.4928663 |  |  |  |  |  |  |  |
|  | nHeF | 3809.322 | 24.151 | 463.2965085 |  |  |  |  |  |  |  |
| L_front_operculum@25862 | HeM | 1570.963 | 2.5 | 241.7772735 | 0.997 | < 0.001 | 0.459 | 0.499 | 0.49 | -0.04 | -0.04 |
|  | HeF | 1452.382 | 2.367 | 241.2366694 |  |  |  |  |  |  |  |
|  | nHeM | 1579.555 | 11.309 | 241.2292143 |  |  |  |  |  |  |  |
|  | nHeF | 1461.047 | 12.597 | 241.6523588 |  |  |  |  |  |  |  |

*significant after adjusting for multiple comparisons across the whole brain

| **(FA-values)**  **FA@UKBiobank_index** | **Group** | **Mean** | **SE** | **SD** | **Sex-by-SSB interaction (p-value)*** | **HeM vs. HeF (p)** | **HeM vs. nHeM (p)** | **HeF vs. nHeF (p)** | **HeM vs. HeF (Cohen's d )** | **HeM vs. nHeM (Cohen's d )** | **HeF vs. nHeF (Cohen's d )** |
| --- | --- | --- | --- | --- | --- | --- | --- | --- | --- | --- | --- |
| FA@25488 | HeM | 0.423 | 0.000266889 | 0.02450736 | 0.706 | <0.001 | 0.286 | 0.636 | 0.12 | -0.08 | -0.04 |
|  | HeF | 0.42 | 0.000251117 | 0.024460442 |  |  |  |  |  |  |  |
|  | nHeM | 0.425 | 0.001233733 | 0.024457802 |  |  |  |  |  |  |  |
|  | nHeF | 0.421 | 0.001344612 | 0.024499999 |  |  |  |  |  |  |  |
| FA@25489 | HeM | 0.417 | 0.000252699 | 0.023204345 | 0.82 | <0.001 | 0.362 | 0.249 | 0.43 | -0.04 | -0.09 |
|  | HeF | 0.407 | 0.000237766 | 0.023159921 |  |  |  |  |  |  |  |
|  | nHeM | 0.418 | 0.001168137 | 0.023157422 |  |  |  |  |  |  |  |
|  | nHeF | 0.409 | 0.001273121 | 0.023197375 |  |  |  |  |  |  |  |
| FA@25490 | HeM | 0.401 | 0.00021254 | 0.019516654 | 0.153 | <0.001 | 0.241 | 0.39 | 0.21 | -0.05 | 0.05 |
|  | HeF | 0.397 | 0.00019998 | 0.01947929 |  |  |  |  |  |  |  |
|  | nHeM | 0.402 | 0.000982494 | 0.019477188 |  |  |  |  |  |  |  |
|  | nHeF | 0.396 | 0.001070794 | 0.019510792 |  |  |  |  |  |  |  |
| FA@25491 | HeM | 0.394 | 0.000209848 | 0.019269441 | 0.19 | <0.001 | 0.114 | 0.749 | 0.26 | -0.05 | 0.00 |
|  | HeF | 0.389 | 0.000197447 | 0.019232551 |  |  |  |  |  |  |  |
|  | nHeM | 0.395 | 0.000970049 | 0.019230475 |  |  |  |  |  |  |  |
|  | nHeF | 0.389 | 0.00105723 | 0.019263653 |  |  |  |  |  |  |  |
| FA@25492 | HeM | 0.539 | 0.000374711 | 0.034408144 | 0.034 | <0.001 | 0.554 | 0.001 | 0.29 | 0.03 | 0.20 |
|  | HeF | 0.529 | 0.000352567 | 0.034342271 |  |  |  |  |  |  |  |
|  | nHeM | 0.538 | 0.001732151 | 0.034338565 |  |  |  |  |  |  |  |
|  | nHeF | 0.522 | 0.001887825 | 0.034397809 |  |  |  |  |  |  |  |
| FA@25493 | HeM | 0.501 | 0.000373313 | 0.034279778 | 0.081 | <0.001 | 0.435 | 0.002 | 0.26 | 0.03 | 0.18 |
|  | HeF | 0.492 | 0.000351252 | 0.03421415 |  |  |  |  |  |  |  |
|  | nHeM | 0.5 | 0.001725689 | 0.034210458 |  |  |  |  |  |  |  |
|  | nHeF | 0.486 | 0.001880782 | 0.034269481 |  |  |  |  |  |  |  |
| FA@25494 | HeM | 0.316 | 0.000338576 | 0.031090098 | 0.778 | <0.001 | 0.399 | 0.691 | 0.32 | 0.03 | 0.03 |
|  | HeF | 0.306 | 0.000318568 | 0.031030577 |  |  |  |  |  |  |  |
|  | nHeM | 0.315 | 0.001565117 | 0.031027228 |  |  |  |  |  |  |  |
|  | nHeF | 0.305 | 0.001705778 | 0.031080759 |  |  |  |  |  |  |  |
| FA@25495 | HeM | 0.316 | 0.000357855 | 0.032860341 | 0.879 | <0.001 | 0.334 | 0.492 | 0.40 | 0.06 | 0.03 |
|  | HeF | 0.303 | 0.000336707 | 0.032797431 |  |  |  |  |  |  |  |
|  | nHeM | 0.314 | 0.001654233 | 0.032793892 |  |  |  |  |  |  |  |
|  | nHeF | 0.302 | 0.001802904 | 0.03285047 |  |  |  |  |  |  |  |
| FA@25496 | HeM | 0.549 | 0.000282 | 0.025894869 | 0.077 | <0.001 | 0.064 | 0.488 | 0.31 | 0.08 | -0.04 |
|  | HeF | 0.541 | 0.000265335 | 0.025845295 |  |  |  |  |  |  |  |
|  | nHeM | 0.547 | 0.001303582 | 0.025842505 |  |  |  |  |  |  |  |
|  | nHeF | 0.542 | 0.001420739 | 0.025887091 |  |  |  |  |  |  |  |
| FA@25497 | HeM | 0.544 | 0.000286841 | 0.026339421 | 0.126 | <0.001 | 0.571 | 0.12 | 0.42 | 0.04 | -0.08 |
|  | HeF | 0.533 | 0.00026989 | 0.026288995 |  |  |  |  |  |  |  |
|  | nHeM | 0.543 | 0.001325961 | 0.026286158 |  |  |  |  |  |  |  |
|  | nHeF | 0.535 | 0.001445129 | 0.02633151 |  |  |  |  |  |  |  |
| FA@25498 | HeM | 0.582 | 0.000320925 | 0.029469243 | 0.775 | 0.784 | 0.456 | 0.281 | 0.00 | 0.03 | 0.07 |
|  | HeF | 0.582 | 0.00030196 | 0.029412825 |  |  |  |  |  |  |  |
|  | nHeM | 0.581 | 0.001483521 | 0.029409651 |  |  |  |  |  |  |  |
|  | nHeF | 0.58 | 0.001616849 | 0.029460391 |  |  |  |  |  |  |  |
| FA@25499 | HeM | 0.465 | 0.000234639 | 0.021545987 | 0.111 | <0.001 | 0.353 | 0.19 | 0.14 | -0.05 | 0.09 |
|  | HeF | 0.462 | 0.000220773 | 0.021504738 |  |  |  |  |  |  |  |
|  | nHeM | 0.466 | 0.001084653 | 0.021502417 |  |  |  |  |  |  |  |
|  | nHeF | 0.46 | 0.001182134 | 0.021539515 |  |  |  |  |  |  |  |
| FA@25500 | HeM | 0.476 | 0.000258492 | 0.023736288 | 0.491 | 0.003 | 0.523 | 0.73 | -0.04 | 0.04 | 0.00 |
|  | HeF | 0.477 | 0.000243217 | 0.023690846 |  |  |  |  |  |  |  |
|  | nHeM | 0.475 | 0.001194916 | 0.02368829 |  |  |  |  |  |  |  |
|  | nHeF | 0.477 | 0.001302307 | 0.023729159 |  |  |  |  |  |  |  |
| FA@25501 | HeM | 0.464 | 0.000244977 | 0.022495268 | 0.477 | 0.368 | 0.58 | 0.649 | 0.00 | 0.00 | -0.04 |
|  | HeF | 0.464 | 0.0002305 | 0.022452202 |  |  |  |  |  |  |  |
|  | nHeM | 0.464 | 0.001132442 | 0.022449779 |  |  |  |  |  |  |  |
|  | nHeF | 0.465 | 0.001234217 | 0.022488511 |  |  |  |  |  |  |  |
| FA@25502 | HeM | 0.459 | 0.000233791 | 0.021468077 | 0.562 | <0.001 | 0.232 | 0.751 | -0.14 | 0.05 | 0.00 |
|  | HeF | 0.462 | 0.000219975 | 0.021426977 |  |  |  |  |  |  |  |
|  | nHeM | 0.458 | 0.001080731 | 0.021424665 |  |  |  |  |  |  |  |
|  | nHeF | 0.462 | 0.00117786 | 0.021461629 |  |  |  |  |  |  |  |
| FA@25503 | HeM | 0.45 | 0.000215446 | 0.019783513 | 0.767 | 0.849 | 0.831 | 0.837 | -0.05 | -0.05 | 0.05 |
|  | HeF | 0.451 | 0.000202714 | 0.019745638 |  |  |  |  |  |  |  |
|  | nHeM | 0.451 | 0.000995928 | 0.019743507 |  |  |  |  |  |  |  |
|  | nHeF | 0.45 | 0.001085435 | 0.01977757 |  |  |  |  |  |  |  |
| FA@25504 | HeM | 0.486 | 0.000336591 | 0.030907776 | 0.954 | <0.001 | 0.635 | 0.605 | 0.32 | 0.03 | 0.03 |
|  | HeF | 0.476 | 0.0003167 | 0.030848604 |  |  |  |  |  |  |  |
|  | nHeM | 0.485 | 0.001555938 | 0.030845275 |  |  |  |  |  |  |  |
|  | nHeF | 0.475 | 0.001695775 | 0.030898492 |  |  |  |  |  |  |  |
| FA@25505 | HeM | 0.424 | 0.000264737 | 0.024309696 | 0.097 | <0.001 | 0.118 | 0.419 | 0.37 | 0.08 | -0.04 |
|  | HeF | 0.415 | 0.000249092 | 0.024263156 |  |  |  |  |  |  |  |
|  | nHeM | 0.422 | 0.001223782 | 0.024260538 |  |  |  |  |  |  |  |
|  | nHeF | 0.416 | 0.001333767 | 0.024302394 |  |  |  |  |  |  |  |
| FA@25506 | HeM | 0.426 | 0.000270558 | 0.024844206 | 0.524 | <0.001 | 0.315 | 0.95 | 0.32 | -0.04 | 0.00 |
|  | HeF | 0.418 | 0.000254569 | 0.024796642 |  |  |  |  |  |  |  |
|  | nHeM | 0.427 | 0.00125069 | 0.024793966 |  |  |  |  |  |  |  |
|  | nHeF | 0.418 | 0.001363093 | 0.024836743 |  |  |  |  |  |  |  |
| FA@25507 | HeM | 0.456 | 0.000246836 | 0.022665931 | 0.735 | <0.001 | 0.557 | 0.316 | -0.13 | 0.04 | 0.04 |
|  | HeF | 0.459 | 0.000232249 | 0.022622538 |  |  |  |  |  |  |  |
|  | nHeM | 0.455 | 0.001141033 | 0.022620096 |  |  |  |  |  |  |  |
|  | nHeF | 0.458 | 0.001243581 | 0.022659122 |  |  |  |  |  |  |  |
| FA@25508 | HeM | 0.454 | 0.000245404 | 0.022534427 | 0.434 | 0.186 | 0.703 | 0.478 | 0.04 | 0.00 | 0.04 |
|  | HeF | 0.453 | 0.000230902 | 0.022491285 |  |  |  |  |  |  |  |
|  | nHeM | 0.454 | 0.001134413 | 0.022488858 |  |  |  |  |  |  |  |
|  | nHeF | 0.452 | 0.001236366 | 0.022527658 |  |  |  |  |  |  |  |
| FA@25509 | HeM | 0.442 | 0.000230603 | 0.021175308 | 0.251 | 0.095 | 0.193 | 0.721 | -0.05 | -0.09 | 0.05 |
|  | HeF | 0.443 | 0.000216975 | 0.021134769 |  |  |  |  |  |  |  |
|  | nHeM | 0.444 | 0.001065993 | 0.021132488 |  |  |  |  |  |  |  |
|  | nHeF | 0.442 | 0.001161797 | 0.021168948 |  |  |  |  |  |  |  |
| FA@25510 | HeM | 0.426 | 0.000224665 | 0.020630038 | 0.205 | <0.001 | 0.053 | 0.948 | 0.10 | -0.10 | 0.00 |
|  | HeF | 0.424 | 0.000211388 | 0.020590543 |  |  |  |  |  |  |  |
|  | nHeM | 0.428 | 0.001038543 | 0.020588321 |  |  |  |  |  |  |  |
|  | nHeF | 0.424 | 0.00113188 | 0.020623841 |  |  |  |  |  |  |  |
| FA@25511 | HeM | 0.426 | 0.000207514 | 0.019055147 | 0.966 | <0.001 | 0.596 | 0.666 | 0.32 | 0.00 | 0.00 |
|  | HeF | 0.42 | 0.000195251 | 0.019018666 |  |  |  |  |  |  |  |
|  | nHeM | 0.426 | 0.000959261 | 0.019016614 |  |  |  |  |  |  |  |
|  | nHeF | 0.42 | 0.001045473 | 0.019049423 |  |  |  |  |  |  |  |
| FA@25512 | HeM | 0.424 | 0.000213861 | 0.019637984 | 0.698 | <0.001 | 0.196 | 0.504 | 0.31 | -0.10 | -0.05 |
|  | HeF | 0.418 | 0.000201223 | 0.019600388 |  |  |  |  |  |  |  |
|  | nHeM | 0.426 | 0.000988602 | 0.019598273 |  |  |  |  |  |  |  |
|  | nHeF | 0.419 | 0.001077451 | 0.019632085 |  |  |  |  |  |  |  |
| FA@25513 | HeM | 0.392 | 0.000259267 | 0.023807412 | 0.982 | <0.001 | 0.293 | 0.346 | 0.25 | -0.04 | -0.04 |
|  | HeF | 0.386 | 0.000243945 | 0.023761834 |  |  |  |  |  |  |  |
|  | nHeM | 0.393 | 0.001198497 | 0.023759269 |  |  |  |  |  |  |  |
|  | nHeF | 0.387 | 0.001306209 | 0.023800261 |  |  |  |  |  |  |  |
| FA@25514 | HeM | 0.391 | 0.00023413 | 0.021499214 | 0.696 | <0.001 | 0.38 | 0.779 | 0.23 | -0.05 | 0.00 |
|  | HeF | 0.386 | 0.000220294 | 0.021458054 |  |  |  |  |  |  |  |
|  | nHeM | 0.392 | 0.001082299 | 0.021455739 |  |  |  |  |  |  |  |
|  | nHeF | 0.386 | 0.001179568 | 0.021492756 |  |  |  |  |  |  |  |

*Note: For FA-values, the overall MANCOVA was not significant for sex-by-SSB (see main text).

| **Sensitivity tests**  **(ROI analysis)** | | control variable --> | Ethnicity | Fluid intelligence score | Smoker current | Smoker previous | BMI | Number of brothers | Number of sisters | Number of older siblings | Birth weight | Victim of physical violence | Victim of sexual abuse | ICV* | Right-handed participants only |
| --- | --- | --- | --- | --- | --- | --- | --- | --- | --- | --- | --- | --- | --- | --- | --- |
| **ROI** | sex-by-SSB interaction main model (p-values) for reference |  | p | p | p | p | p | p | p | p | p | p | p | p | p |
| Calcarine* | 0.000 |  | 0.000 | 0.001 | 0.000 | 0.000 | 0.000 | 0.000 | 0.000 | 0.001 | 0.000 | 0.000 | 0.000 | 0.007 | 0.001 |
| PFC/OFC* | 0.001 |  | 0.001 | 0.004 | 0.001 | 0.001 | 0.002 | 0.002 | 0.002 | 0.003 | 0.104 | 0.003 | 0.002 | 0.236 | 0.002 |
| Precuneus* | 0.006 |  | 0.004 | 0.009 | 0.008 | 0.006 | 0.012 | 0.009 | 0.008 | 0.034 | 0.131 | 0.058 | 0.050 | 0.432 | 0.029 |
| Inferior temporal* | 0.008 |  | 0.006 | 0.010 | 0.007 | 0.005 | 0.010 | 0.007 | 0.006 | 0.021 | 0.013 | 0.060 | 0.058 | 0.341 | 0.007 |
| Thalamus* | 0.015 |  | 0.012 | 0.065 | 0.023 | 0.014 | 0.025 | 0.018 | 0.017 | 0.110 | 0.122 | 0.008 | 0.006 | 0.785 | 0.069 |
| Fusiform | 0.016 |  | 0.014 | 0.044 | 0.022 | 0.015 | 0.031 | 0.023 | 0.021 | 0.012 | 0.330 | 0.049 | 0.054 | 0.790 | 0.012 |
| Lingual | 0.043 |  | 0.041 | 0.213 | 0.053 | 0.048 | 0.041 | 0.052 | 0.047 | 0.128 | 0.391 | 0.305 | 0.286 | 0.943 | 0.082 |
| ACC | 0.045 |  | 0.038 | 0.066 | 0.041 | 0.041 | 0.063 | 0.058 | 0.061 | 0.130 | 0.297 | 0.034 | 0.034 | 0.584 | 0.105 |
| Cuneus | 0.147 |  | 0.129 | 0.325 | 0.172 | 0.143 | 0.262 | 0.243 | 0.241 | 0.419 | 0.199 | 0.514 | 0.429 | 0.950 | 0.223 |
| Parahippocampal | 0.577 |  | 0.569 | 0.843 | 0.690 | 0.621 | 0.692 | 0.633 | 0.633 | 0.735 | 0.821 | 0.392 | 0.361 | 0.213 | 0.481 |
| Hippocampus | 0.775 |  | 0.756 | 0.991 | 0.905 | 0.836 | 0.863 | 0.780 | 0.780 | 0.951 | 0.809 | 0.319 | 0.289 | 0.300 | 0.955 |

| **Sensitivity tests**  **(ROI analysis)** | | | control variable --> | | Bipolar disorder | | Manic episode | | MDD (single episode) | | MDD | | Substance-use disorder | | Alcohol-use disorder | | Phobia/Anxiety disorder | | Panic disorder | | OCD | | Stress-related disorder | | Psychotic disorder incl. schizophrenia | | Personality disorder | | Autism | | Eating disorder | | All psychiatric diagnoses as covariates | Having any psychiatric diagnosis | Excluding individuals with any psychiatric diagnosis |
| --- | --- | --- | --- | --- | --- | --- | --- | --- | --- | --- | --- | --- | --- | --- | --- | --- | --- | --- | --- | --- | --- | --- | --- | --- | --- | --- | --- | --- | --- | --- | --- | --- | --- | --- | --- |
| **ROI** | sex-by-SSB interaction main model (p-values) for reference |  | | p | | p | | p | | p | | p | | p | | p | | p | | p | | p | | p | | p | | p | | p | | p | | p | p |
| Calcarine* | 0.000 |  | | 0.000 | | 0.000 | | 0.000 | | 0.000 | | 0.000 | | 0.000 | | 0.000 | | 0.000 | | 0.000 | | 0.000 | | 0.000 | | 0.000 | | 0.000 | | 0.000 | | 0.000 | | 0.000 | 0.001 |
| PFC/OFC* | 0.001 |  | | 0.001 | | 0.001 | | 0.001 | | 0.001 | | 0.002 | | 0.002 | | 0.001 | | 0.001 | | 0.001 | | 0.001 | | 0.001 | | 0.001 | | 0.001 | | 0.001 | | 0.002 | | 0.001 | 0.003 |
| Precuneus* | 0.006 |  | | 0.006 | | 0.006 | | 0.006 | | 0.006 | | 0.008 | | 0.007 | | 0.006 | | 0.006 | | 0.006 | | 0.006 | | 0.006 | | 0.006 | | 0.006 | | 0.006 | | 0.007 | | 0.007 | 0.008 |
| Inferior temporal* | 0.008 |  | | 0.008 | | 0.008 | | 0.008 | | 0.008 | | 0.010 | | 0.009 | | 0.008 | | 0.008 | | 0.008 | | 0.007 | | 0.008 | | 0.008 | | 0.008 | | 0.008 | | 0.009 | | 0.009 | 0.018 |
| Thalamus* | 0.015 |  | | 0.015 | | 0.015 | | 0.014 | | 0.014 | | 0.023 | | 0.020 | | 0.014 | | 0.015 | | 0.015 | | 0.014 | | 0.014 | | 0.016 | | 0.015 | | 0.015 | | 0.022 | | 0.017 | 0.031 |
| Fusiform | 0.016 |  | | 0.016 | | 0.016 | | 0.016 | | 0.015 | | 0.022 | | 0.021 | | 0.015 | | 0.016 | | 0.016 | | 0.015 | | 0.016 | | 0.016 | | 0.016 | | 0.016 | | 0.021 | | 0.018 | 0.017 |
| Lingual | 0.043 |  | | 0.043 | | 0.043 | | 0.043 | | 0.041 | | 0.051 | | 0.049 | | 0.041 | | 0.044 | | 0.043 | | 0.042 | | 0.043 | | 0.042 | | 0.043 | | 0.043 | | 0.046 | | 0.046 | 0.082 |
| ACC | 0.045 |  | | 0.043 | | 0.044 | | 0.045 | | 0.045 | | 0.042 | | 0.041 | | 0.045 | | 0.044 | | 0.045 | | 0.046 | | 0.045 | | 0.045 | | 0.045 | | 0.044 | | 0.042 | | 0.043 | 0.101 |
| Cuneus | 0.147 |  | | 0.146 | | 0.147 | | 0.147 | | 0.145 | | 0.175 | | 0.167 | | 0.147 | | 0.148 | | 0.147 | | 0.142 | | 0.147 | | 0.150 | | 0.147 | | 0.147 | | 0.175 | | 0.154 | 0.324 |
| Parahippocampal | 0.577 |  | | 0.585 | | 0.577 | | 0.576 | | 0.569 | | 0.652 | | 0.624 | | 0.564 | | 0.578 | | 0.577 | | 0.561 | | 0.579 | | 0.584 | | 0.577 | | 0.578 | | 0.622 | | 0.601 | 0.665 |
| Hippocampus | 0.775 |  | | 0.785 | | 0.775 | | 0.774 | | 0.767 | | 0.905 | | 0.867 | | 0.763 | | 0.779 | | 0.775 | | 0.763 | | 0.771 | | 0.788 | | 0.775 | | 0.774 | | 0.900 | | 0.812 | 0.985 |

Overall, the ROI results remained robust when controlling for potential confounders. Notably, the calcarine finding also remained when adjusting for ICV (*note, however, that we argue against ICV correction when investigating cross-sex shifts). The somewhat lower significance in some cases may be explained by a) reduced sample size, as not all participants provided the respective information, and b) the testing variable being associated with the outcome of interest (Hyatt et al., 2020), e.g., a person could become victim of physical violence because of their sexual orientation.
